# Synthesis and biological evaluation of potent benzoselenophene and heteroaromatic analogues of (*S*)-1-(chloromethyl)-8-methoxy-2,3-dihydro-1*H*-benzo[*e*]indol-5-ol (*seco*-MCBI)†

**DOI:** 10.1039/c9ra04749b

**Published:** 2019-09-16

**Authors:** Amol B. Mhetre, Eppakayala Sreedhar, Rashmi Dubey, Ganesh A. Sable, Hangeun Lee, Heekyoung Yang, Kyoungmin Lee, Do-Hyun Nam, Dongyeol Lim

**Affiliations:** Department of Chemistry, Sejong University Seoul 143-747 Republic of Korea dylim@sejong.ac.kr; Department of Neurosurgery, Samsung Medical Center, Sungkyunkwan University School of Medicine Seoul Republic of Korea

## Abstract

A diverse series of compounds (18a–x) were synthesized from (*S*)-1-(chloromethyl)-8-methoxy-2,3-dihydro-1*H*-benzo[*e*]indol-5-ol (*seco*-MCBI) and benzoselenophene or heteroaromatic acids. These new compounds were evaluated for their cytotoxicity against the human gastric NCI-N87 and human ovarian SK-OV3 cancer cell lines. The incorporation of a methoxy substituent at the C-7 position of the *seco*-CBI unit enhances the cytotoxicity through its additional van der Waals interaction and gave a much higher potency than the corresponding *seco*-CBI-based analogues. Similarly, the *seco*-MCBI-benzoselenophene conjugates (18h–x) exhibited substitution effects on biological activity, and the *N*-butyramido and *N*-methylthiopropanamido analogues are highly potent, possessing >77- and >24-fold better activity than *seco*-MCBI-TMI for the SK-OV3 and NCI-N87 cell lines, respectively.

## Abstract 2

Incorporation of a methoxy substituent at the C-7 position of CBI and *N*-amido substitution at the C-5 position of benzoselenophene significantly enhances the anticancer activity of duocarmycin analogs.

## Graphical abstract

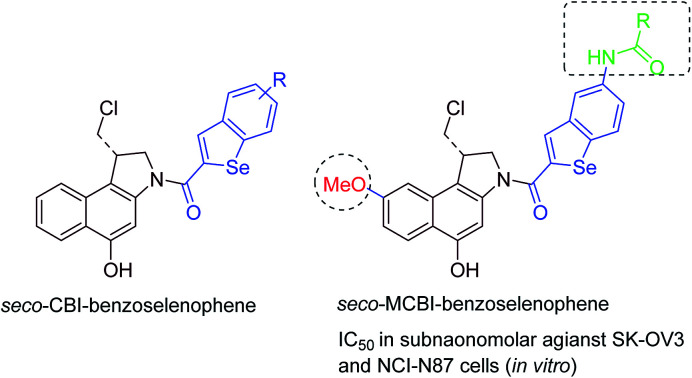

## Introduction

Cancer is considered one of the major causes of death worldwide.^1^ Tremendous resources are being invested worldwide to develop preventive, diagnostic, and therapeutic strategies for cancer.^2^ In recent years, antibody-drug conjugates (ADCs) have become an effective class of therapeutic agents for cancer therapy.^3^ The concept was introduced over 30 years ago to improve the therapeutic index of anticancer drugs.^4^ ADCs have potential target selectivity towards tumour cells compared to conventional chemotherapy;^5^ however, their use is complicated in practice because cell-surface antigens are often limited and the process of internalization is inefficient. Assuming all steps involved in the mechanism of ADC have an efficiency of 50%, only 1–2% of the administered drug will reach tumour cells. This makes the choice of a cytotoxin particularly important because it is required to be highly efficacious at very low concentrations.^6^

**Fig. 1:**
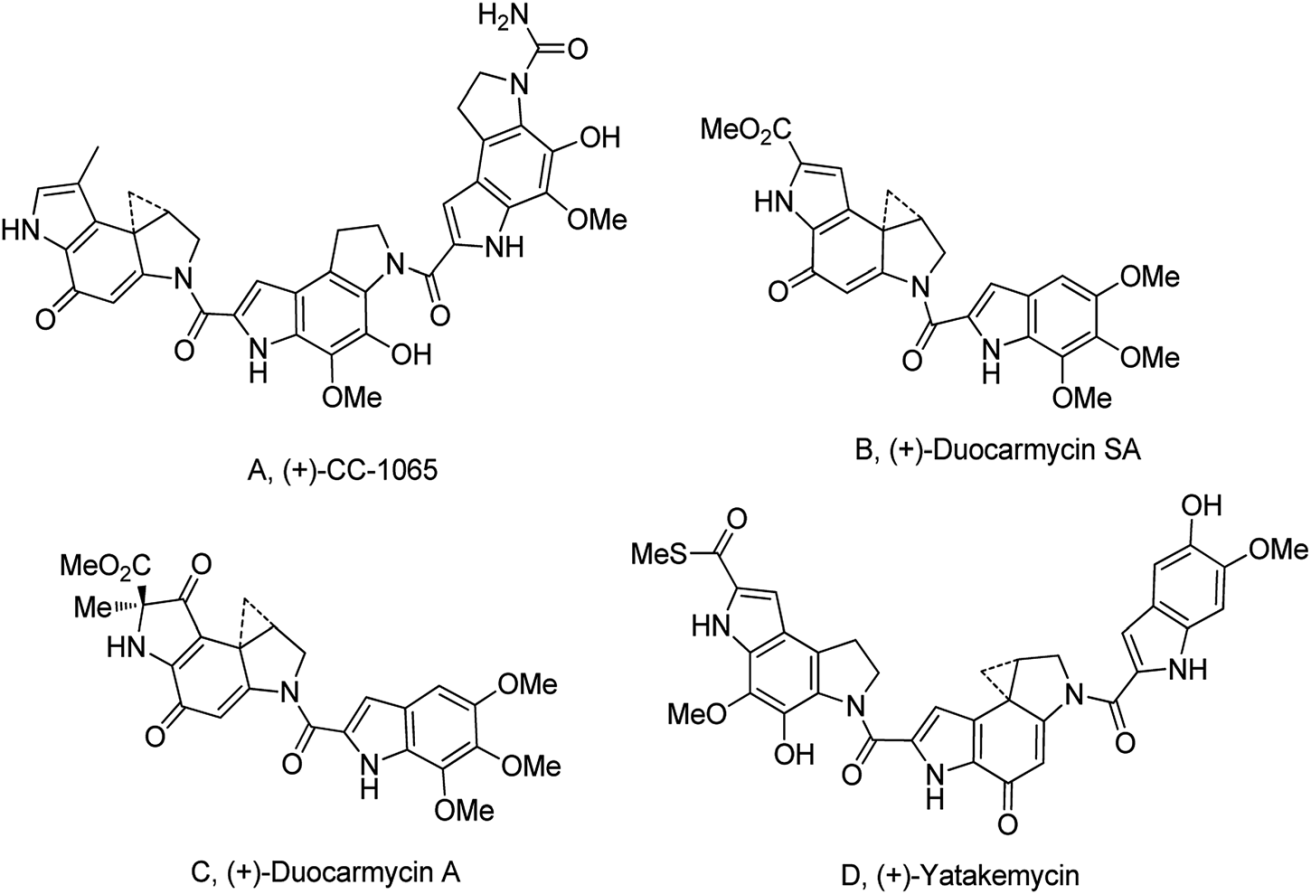
Natural product (DNA alkylating agents).^21^

**Fig. 2:**
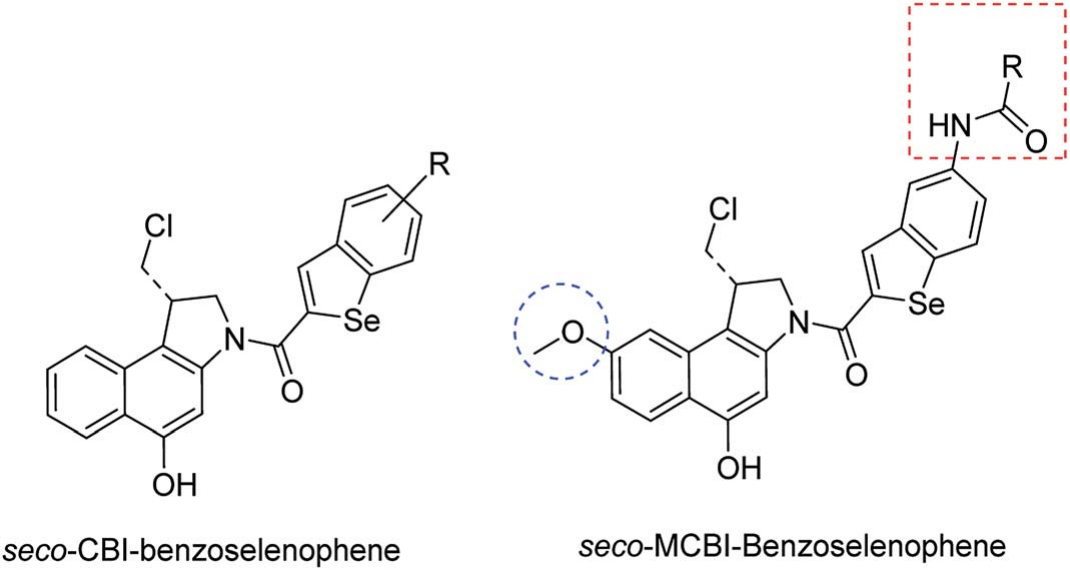
Structural modification of *seco*-CBI benzoselenophene.

The cyclopropylpyrrolo[*e*]indolone (CPI)-containing alkaloids, *i.e.*, CC-1065, and the duocarmycin class of compounds^7–11^ (Fig. 1) attracted our interest due to their higher antitumour activity. They are effective at picomolar concentrations against L1210 cell assay. These natural products have biological properties and therapeutic efficacy that are determined by their capacity for characteristic duplex DNA alkylation and DNA binding affinity.^12–16^ The study of natural products and their synthetic derivatives has defined the fundamental features that control the selectivity and efficiency of DNA alkylation.^17–20^ In our earlier study, we synthesized and evaluated the *in vitro* cytotoxicity of benzoselenophene analogues of *seco*-CBI.^21^ It has been demonstrated that the benzoselenophene was a good substitute as a DNA binding unit for the indole moiety of duocarmycin analogues, which helps to improve the biological activity through increased curvature and hydrophobicity. To further enhance the biological activity, we used (*S*)-1-(chloromethyl)-8-methoxy-2,3-dihydro-1*H*-benzo[*e*]indol-5-ol (*i.e.*, *seco*-MCBI) as a DNA alkylating agent in this study. The magnitude of the electronic effect of C-7 methoxy substituent of *seco*-CBI affects the reactivity of DNA alkylation and the solvolysis rate, providing additional noncovalent interactions.^22^ These features encourage us to prepare *seco*-MCBI-benzoselenophene conjugates (Fig. 2). Similarly, the substituents attached to the DNA binding unit not only provide DNA binding affinity and selectivity, but they also affect the rate and efficiency of DNA alkylation and biological activity.^23^ To evaluate the substituent effect on activity, we designed and synthesized differently substituted benzoselenophene analogues. We also aimed to develop a hydrophilic drug that does not compromise the cytotoxicity but that will instead help to improve the aqueous solubility for preparing non-aggregated ADCs. It can be achieved by attaching a hydrophilic substituent at the benzoselenophene unit or by introducing a hetero atom at a selected position in the side chain, which maintains both binding affinity and aqueous solubility. In view of the above-mentioned observation and our goal of preparing highly potent candidates, we describe the syntheses and anticancer activities of benzoselenophene and heteroaromatic conjugates of *seco*-MCBI.

## Results and discussion

### Chemistry

**Scheme 1:**
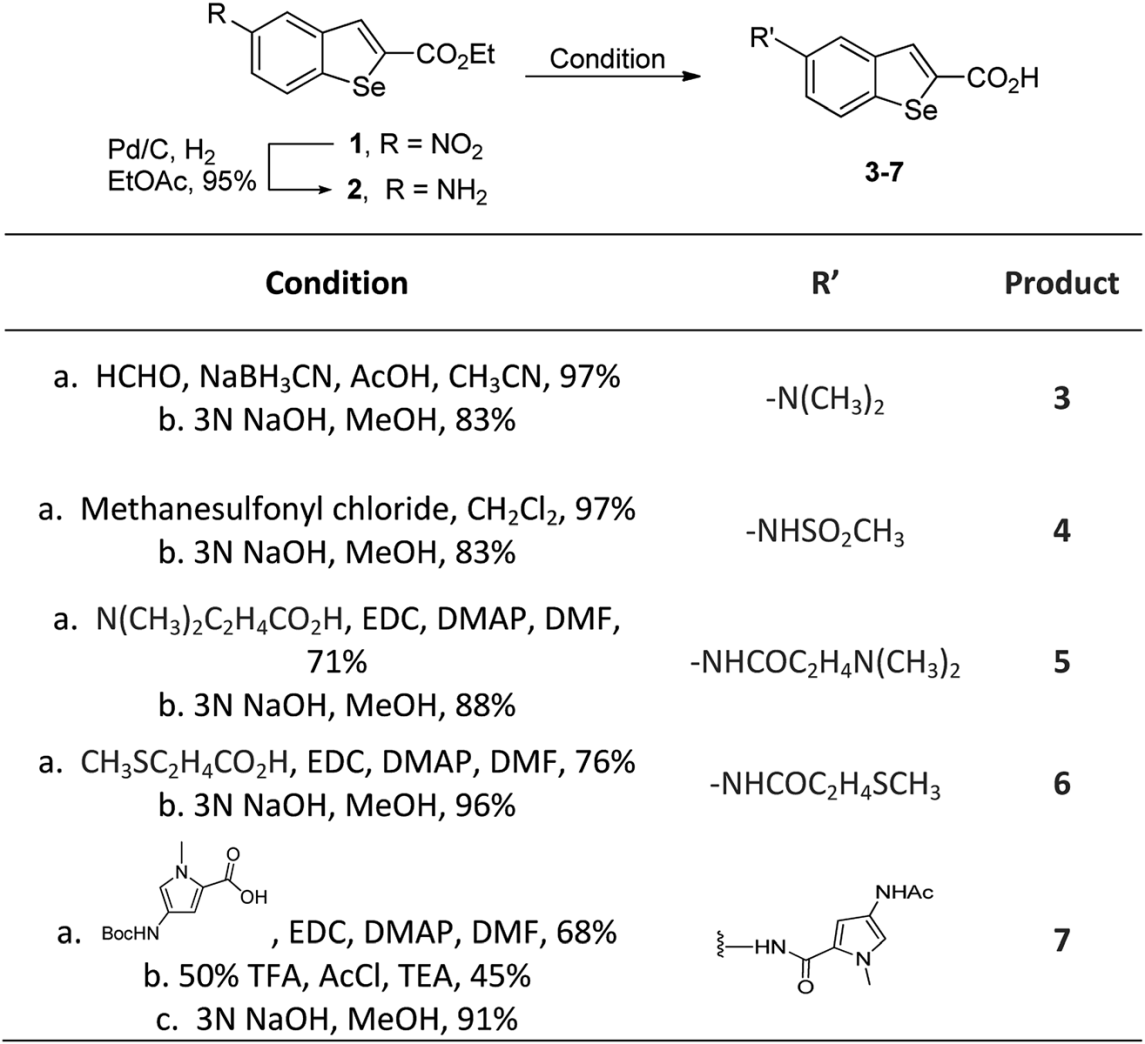
Synthesis of *N*-substituted benzoselenophene analogues.

In our previous work, we synthesized various selenophene-fused aromatic compounds with the aim of using them as DNA binding units.^21,24^ Because the substitution at the C-5 position of the DNA binding unit in duocarmycin analogues is more effective for enhancing the cytotoxicity against cancer cell lines, we mostly focused on the synthesis of C-5-substituted benzoselenophene analogues.^25,26^ The nitro analogue 1 is the key substrate to prepare a diverse series of *N*-substituted amido analogues. First, palladium-catalysed reduction of nitro group was performed to acquire amine 2. Then, the resultant amine 2 was converted into *N*-substituted amino and various amido analogues, which hydrolysed to provide their corresponding acids (3–7) with good overall yields. Other required benzoselenophene carboxylic acids were prepared by our previously reported method (Scheme 1).^21,24^

**Scheme 2:**
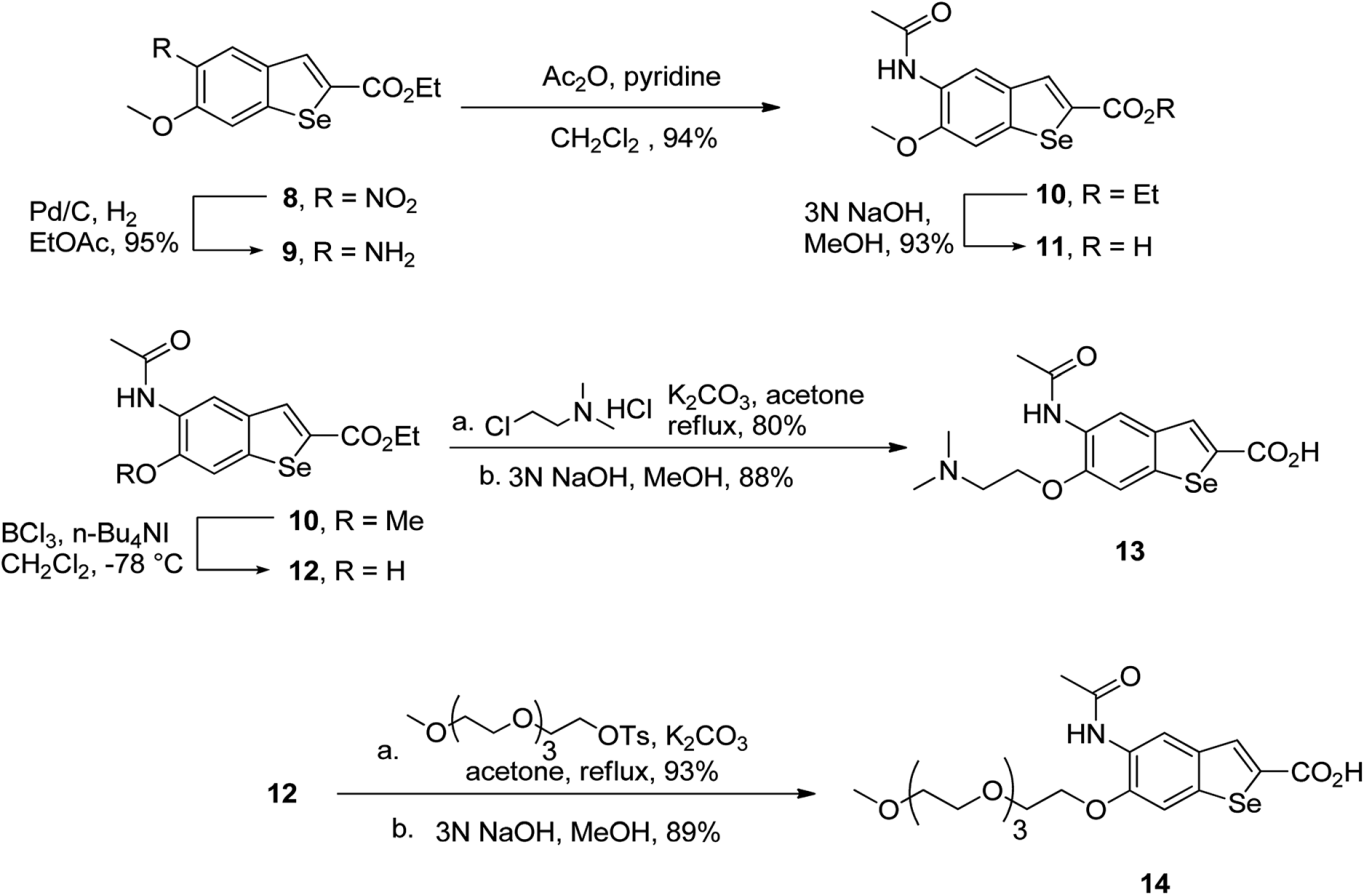
Synthesis of 6-alkoxy 5-acetamidobenzoselenophene carboxylic acids.

To prepare hydrophilic benzoselenophene analogues, the 6-methoxy-substituted nitro compound 8 was used as a starting material. It was converted to the corresponding amine 9 in a palladium-catalysed nitro reduction. Then, the 6-methoxy substituted acetamido analogue 10 was prepared by treating 9 with acetic anhydride in pyridine. The resultant intermediate 10 was hydrolysed to acquire acid 11. To prepare other 6-alkoxy-substituted acetamido analogues, demethylation of 10 was accomplished by BCl_3_ and tetra(*n*-butyl)ammonium iodide, yielding the free phenol 12. The resultant analogue 12 was treated with 2-chloro-*N*,*N*-dimethylethanamine under basic conditions to obtain an *N*,*N*-dimethylethoxy derivative of benzoselenophene ester, which was hydrolysed to generate carboxylic acid 13. Similarly, pegylation of analogue 12 was performed by treatment with the tosyl protected Peg_5_-OH under basic conditions, and then the resultant ester intermediate was hydrolysed to give 14 (Scheme 2).

**Scheme 3:**
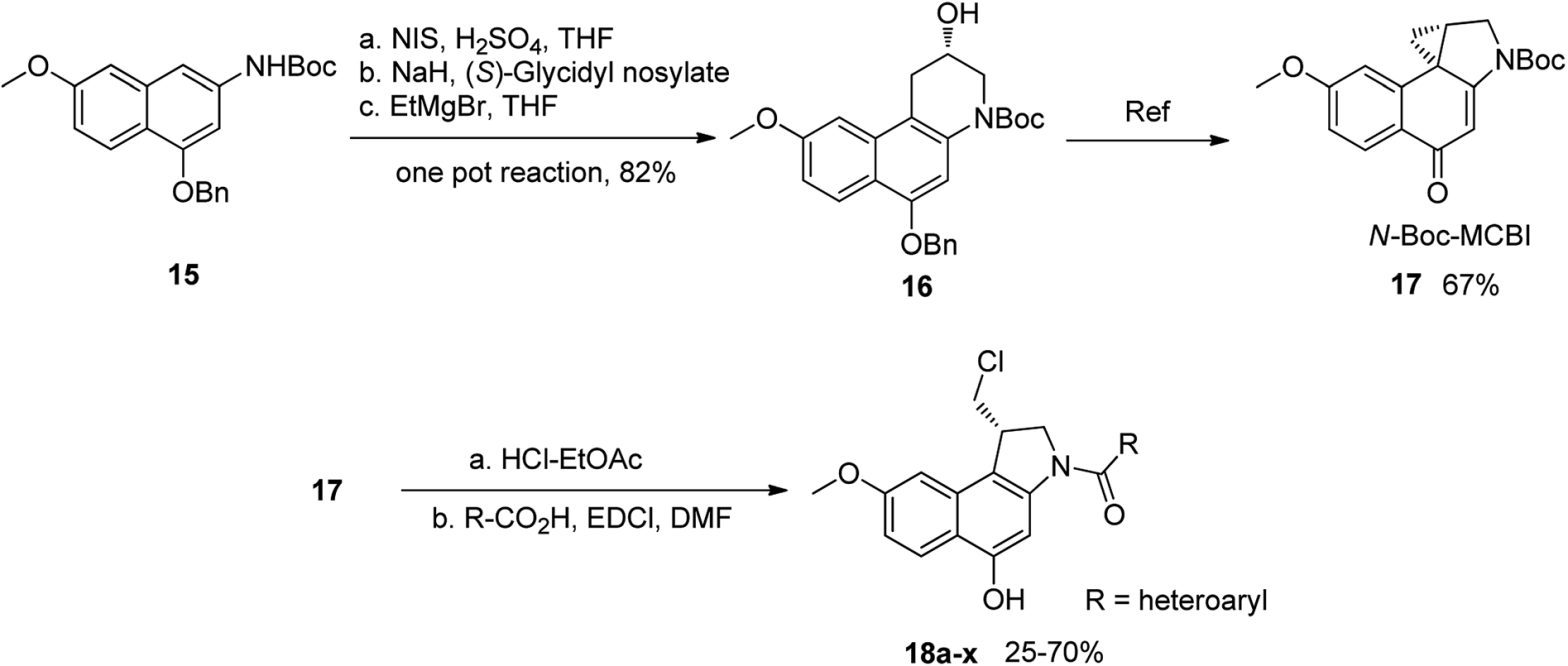
Synthesis of *N*-Boc-MCBI and 18a–x.

To prepare *seco*-MCBI, we first synthesized intermediate 15 from 3-methoxy benzaldehyde according to a previously reported procedure.^22^ Initially, we used a known method to make intermediate 16 from 15 by following three different previously described reactions.^27^ The overall yield was low because of first step, in which the iodo intermediate was found to be unstable and degraded during purification. To overcome this problem, we performed one-pot synthesis of 16 from 15 through a sequence of iodination, alkylation and cyclization, which provided the desired product with an excellent yield (82%). After obtaining intermediate 16, we followed the reported procedure to prepare *N*-Boc-MCBI 17 by mesylation, debenzylation and cyclization reactions.^28–30^ Next, the heteroaryl-*seco*-MCBI conjugate, 18a–w, were synthesized by *N*-Boc deprotection of 17 in 4 N HCl in ethyl acetate and by coupling with different carboxylic acids using EDCI as a coupling reagent (Scheme 3).

### Biological activity

Different derivatives of *seco*-MCBI-benzoselenophene, *seco*-MCBI-heteroaromatic analogues (18a–x) along with *seco*-MCBI-TMI,^22^*seco*-CBI-TMI^31^ and various *seco*-CBI-heteroaromatic analogues (19a–d) were examined for their *in vitro* activity against the human gastric NCI-N87 and human ovarian SK-OV3 cancer cell lines. The cells were seeded in 384-well plates at 500 cells per well and were then treated with compounds in five-fold serial dilution. After 3 days of incubation at 37 °C, the cell viability was checked using an adenosine triphosphate monitoring system based on firefly luciferase. The 5,6,7-trimethoxyindole (TMI) derivatives of *seco*-CBI and *seco*-MCBI were considered highly potent candidates of the duocarmycin class of compounds and used for activity comparison.^13^ Although a previous study explained the electronic effect of the C-7 methoxy group of *seco*-CBI (*i.e.*, *seco*-MCBI) on the functional reactivity of the agents, it had little or no perceptible effect on the biological activities against L1210 cell lines compared to the corresponding *seco*-CBI-based agent.^22^ In our study, the *seco*-MCBI-TMI was 6 and 12 times more potent than *seco*-CBI-TMI in the SK-OV3 (IC_50_ = 5.4 *versus* 30 pM) and NCI-N87 (IC_50_ = 11 *versus* 130 pM) cell assays, respectively (Table 1). However, a significant activity difference was observed between heteroaromatic analogues (selenophene-fused pyridine, furan and thiophene) of *seco*-CBI and *seco*-MCBI. All heteroaromatic conjugates of *seco*-MCBI 18a–c have been found to be much more effective against both cell lines than the corresponding *seco*-CBI analogues 19a–c (Table 1). It was also observed that the selenophene-fused analogues of *seco*-MCBI and *seco*-CBI are more active against the SK-OV3 cell line than against the NCI-N87 cell line.

**Table 1:** Comparison between *seco*-MCBI and *seco*-CBI analogues

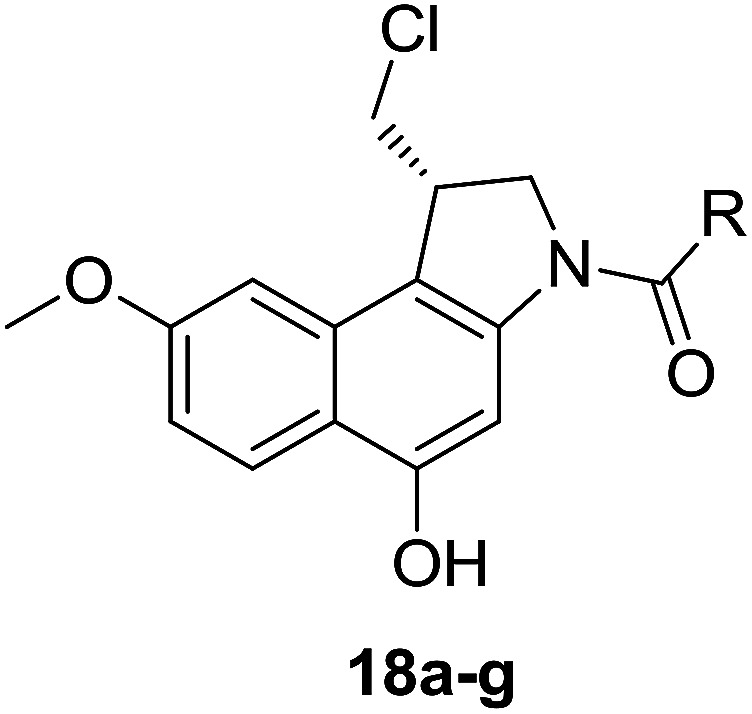				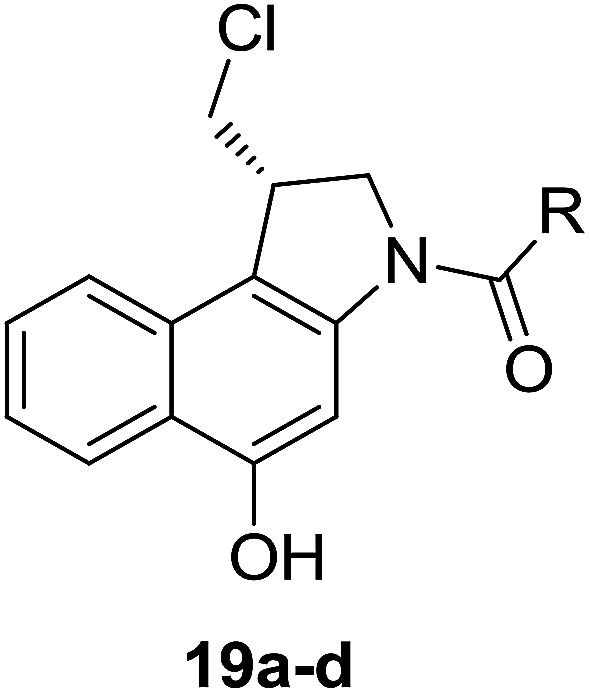			
Compound	R	IC_50_^a^ (pM)		Compound	R	IC_50_^a^ (pM)	
		NCI-N87^b^	SK-OV3^c^			NCI-N87^b^	SK-OV3^c^
*seco*-MCBI-TMI	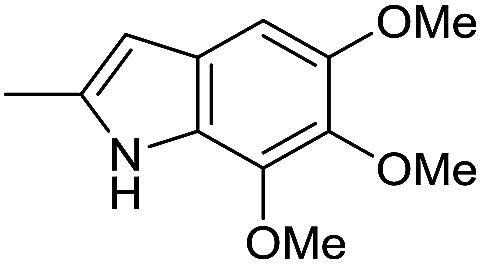	11	5.4	*seco*-CBI-TMI	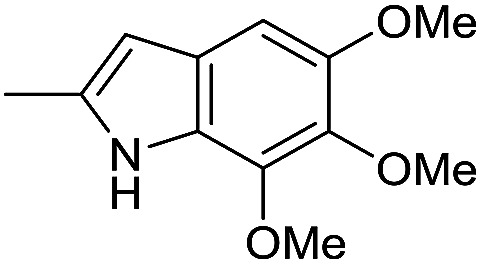	130	30
18a	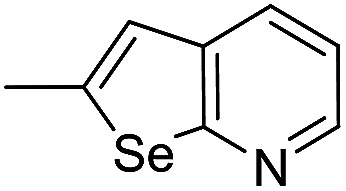	24	9.2	19a	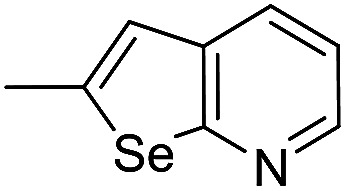	5200	3800
18b	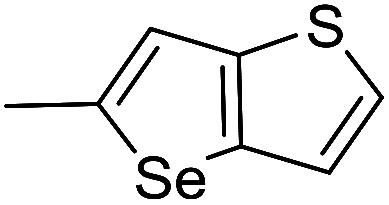	0.79	0.6	19b	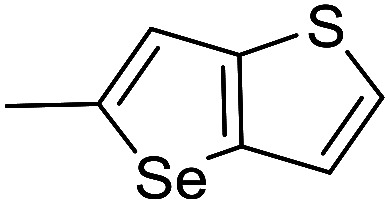	2000	1000
18c	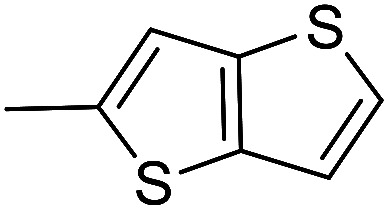	15	13.2	19c	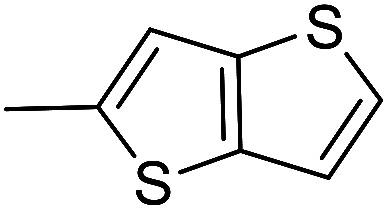	1100	370
18d	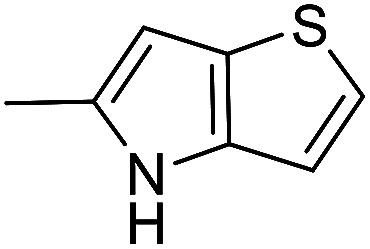	ND^d^	ND^d^	19d	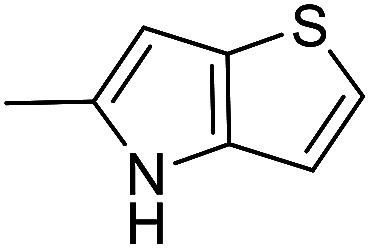	1500	750
18e	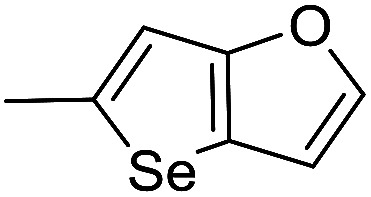	13	0.18	—	—	—	—
18f	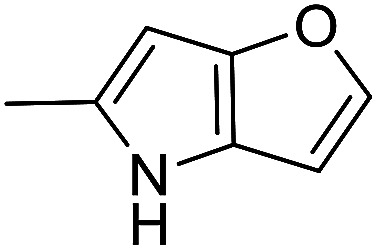	680	280	—	—	—	—
18g	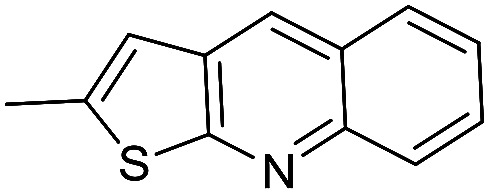	29	2.8	—	—	—	—

a IC_50_ values were calculated as an average of quadruplicate experiments.

b NCI-N87: human gastric cancer cell line.

c SK-OV3: human ovarian cancer cell line.

d Not determined.

The methoxy substitution effect on the biological activity of *seco*-MCBI-benzoselenophene analogues occurred in the order C-5 > C-6 > C-7 for the SK-OV3 cell line and C-7 ≈ C-5 > C-6 for the NCI-N87 cell line (Table 2). The activity for SK-OV3 was similar to that of the corresponding moieties of *seco*-CBI-indole analogues against L1210 leukaemia cell lines.^26^ Compound 18h with C-5 OMe was only 1.4- and 2.3-fold less potent than the *seco*-MCBI-TMI analogue against SK-OV3 (IC_50_ = 7.7 *versus* 5.4 pM) and NCI-N87 (IC_50_ = 26 *versus* 11 pM) cell lines, respectively (Table 2). These results indicate that the C-5-substituted benzoselenophene analogues are more effective at enhancing their cytotoxicity than the C-6 and C-7 substituted benzoselenophene analogues are.

**Table 2:** Methoxy-substituted benzoselenophene analogues of *seco*-MCBI

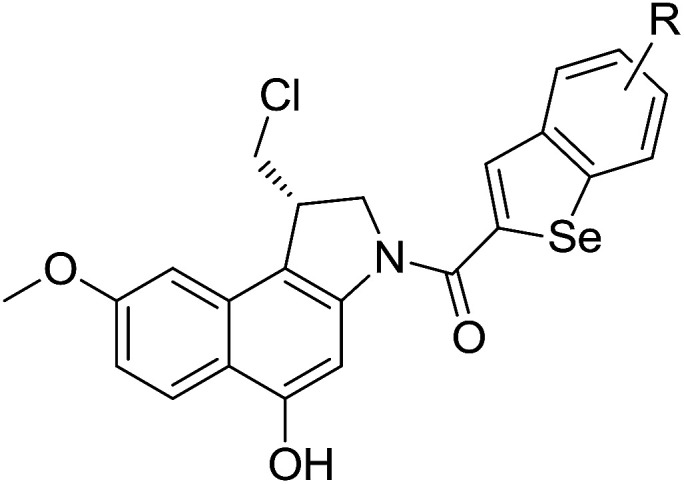			
Compound	R	IC_50_^a^ (pM)	
		NCI-N87	SK-OV3
*seco*-MCBI-TMI	—	11	5.4
18h	5-OMe	26	7.7
18i	6-OMe	91	12
18j	7-OMe	22	96
18k	5,6-Dimethoxy	ND	ND

a IC_50_ values were calculated as an average of quadruplicate experiments.

In our preliminary study, we found that the *N*-substituted benzoselenophene analogues at the C-5 position are more cytotoxic.^21^ Therefore, we prepared different *N*-substitute *seco*-MCBI-benzoselenophene analogues and examined their cytotoxicity (Table 3). In a previously reported study,^26^ the *seco*-CBI-indole analogues with NO_2_, NMe_2_, NHCOMe and NHCOPr substituents at the C-5 position have similar activities (IC_50_ = 20–40 pM against L1210) in our study, we observed a significant difference in the biological activity for the corresponding benzoselenophene analogues. For example, 18l, a nitro-substituted analogue, was less cytotoxic (IC_50_ = 22 700 and 5000 pM against NCI-N87 and SK-OV3, respectively) than 18m with an NMe_2_ substituent (IC_50_ = 490 and 65 pM against NCI-N87 and SK-OV3, respectively). 18n, with an *N*-acetamido moiety (IC_50_ = 1.7 and 0.2 pM against NCI-N87 and SK-OV3, respectively), was highly potent, surpassing the cytotoxicity of *seco*-MCBI-TMI. To increase the hydrophilic properties of the cytotoxic agent, we prepared analogues 18p–q with *N*,*N*-dimethylethoxy and Peg_5_ substituents, respectively, at the C-6 position of the acetamido analogue 18n, but diminished activities were observed compared to that of analogue 18n. Interestingly, for the compound 18r with a *N*-butyramido substituent, 5- and 3-fold enhancements in activities were observed against NCI-N87 and SK-OV3 cells, respectively, compared to 18n, but no improvements were observed for 18s, with an *N*-hexanamide substituent. To achieve further enhancement in the activity, we replaced the γ-carbon of the *N*-butyramide substituent of 18r with an *N*,*N*-dimethyl amine and *S*-Me group, resulting in analogues 18t and 18u, respectively. The 18t has significantly reduced potency against both cell lines, while 3.5-fold improvement was observed for 18u against the SK-OV3 cell line. The substituted pyrrole 18v was found to be 2 times less potent than the 18n against the SK-OV3 cell line, but it was >10 times more potent than the *seco*-MCB-TMI analogue against both cell lines. The activity was dramatically reduced in sulfonamide derivatives 18w–x, which were >2200 times less potent than *seco*-MCBI-TMI. This may due to the poor interaction of the sp^3^ hybridized sulfonyl group in the minor groove. Overall, in this series of *N*-substituted benzoselenophene analogues, the *N*-butyramido (18r) and methylthiopropanamido (18u) analogues were found to be the most potent, exhibiting IC_50_ values < 1 pM against both cell lines in the current assay.

**Table 3:** C-5 amido-substituted benzoselenophene analogues of *seco*-MCBI

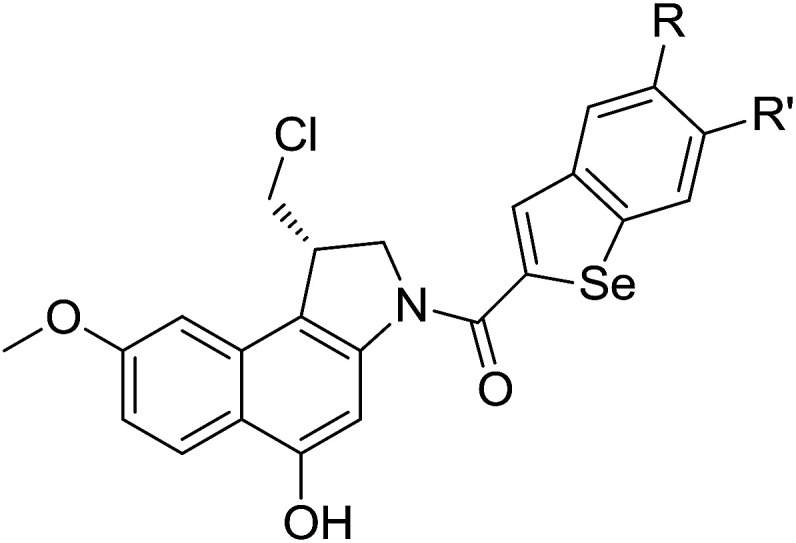				
Compound	R	R′	IC_50_^a^ (pM)	
			NCI-N87	SK-OV3
18l	–NO_2_	H	23 000 (230 nM)	5000
18m	–NMe_2_	H	490	65
18n	–NHAc	H	1.7	0.2
18o	–NHAc	OMe	ND	ND
18p	–NHAc	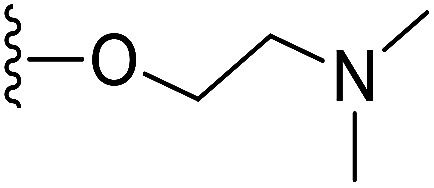	190	37
18q	–NHAc	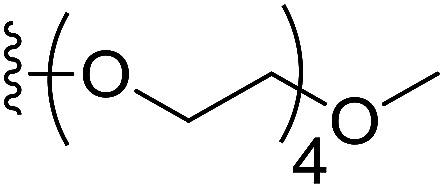	1000	260
18r	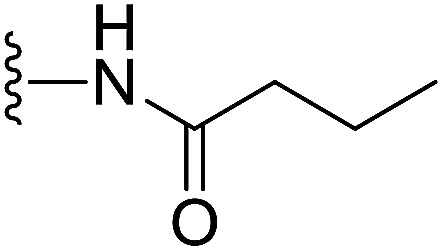	H	0.35	0.07
18s	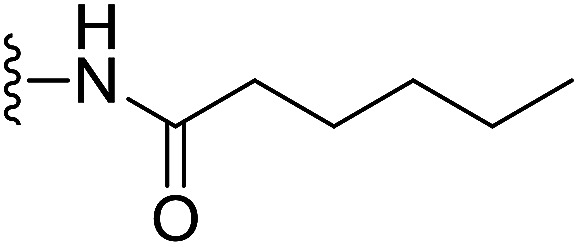	H	16	12
18t	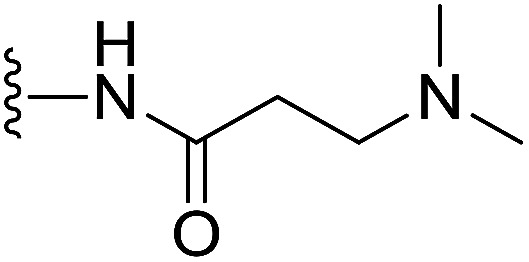	H	55	42
18u	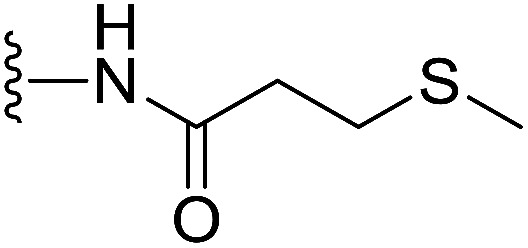	H	0.46	0.02
18v	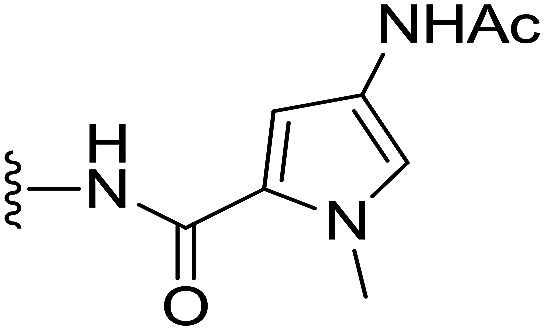	H	1.3	0.44
18w	–NHSO_2_Me	H	120 000 (120 nM)	53 000 (53 nM)
18x	–NHSO_2_Me	OMe	25	22

a IC_50_ values were calculated as an average of quadruplicate experiments.

## Experimental section

### General materials and methods

All reagents were obtained from commercial suppliers and used without further purification, unless specified. The starting carboxylic acids of the analogues 18c–d and 18f–g*i.e.* thieno[3,2-*b*]thiophene-2-carboxylic acid, 4*H*-thieno[3,2-*b*]pyrrole-5-carboxylic acid, 4*H*-furo[3,2-*b*]pyrrole-5-carboxylic acid and thieno[2,3-*b*]quinoline-2-carboxylic acid respectively, were purchased from Aldrich. Dry DMF and ethyl acetate were purchased from Sigma Aldrich (>99.9%). Tetrahydrofuran (THF) and dichloromethane (CH_2_Cl_2_) were distilled over sodium and benzophenone. A saturated solution of HCl in ethyl acetate was prepared by purging pure HCl gas (99.99%, manufactured by RIGAS, Korea) in dry ethyl acetate at 0 to −5 °C for 1 h and stored in a deep freezer. ^1^H and ^13^C NMR spectra were collected at resonance frequencies of 500.1 and 125.7 MHz, respectively. The solvents used for NMR were DMSO-d_6_, acetone-d_6_, CDCl_3_ and CD_3_OD as indicated. The chemical shifts for ^1^H NMR are reported in ppm from tetramethylsilane (0 ppm) or referenced to the solvent (DMSO-d_6_ 2.50; acetone-d_6_ 2.05; CD_3_OD 3.31 and CDCl_3_ 7.26 ppm) on the *δ* scale. Chemical shifts (*δ*) for ^13^C NMR spectra are referenced to the signals for residual deuterated solvents (DMSO-d_6_ 39.5; acetone-d_6_ 29.84, 206.26; CD_3_OD 49.00 and CDCl_3_ 77.16 ppm). Multiplicities are reported by the following abbreviations: s (singlet), d (doublet), t (triplet), q (quartet), m (multiplet), dd (doublet of doublet), brs (broad singlet), *J* (coupling constants in hertz). Analytical reverse-phase high performance liquid chromatography (RP-HPLC) was carried out using a C18 (4.6 × 150 mm) reverse-phase column at a flow rate of 1 mL min^−1^ with UV detection at 214 and 254 nm. Linear gradients of CH_3_CN/H_2_O solvents, each containing 0.1% TFA were used as follows: condition A (10 to 80% CH_3_CN gradient over 20 min), condition B (30 to 100% CH_3_CN gradient over 20 min). For preparative HPLC, a C18 column (5 μm, 10 × 250 mm) was employed at a flow rate of 4 mL min^−1^ using the gradient condition B. High resolution mass spectra (HRMS) were recorded using two different instruments: (i) fast atom bombardment ionization using a double-focusing magnetic sector mass analyzer (ii) electrospray ionization using an ion trap analyzer. All reactions were monitored by thin-layer chromatography (TLC) performed on glass packed silica gel plates (60F-254) with UV light and visualized with ninhydrin, *p*-anisaldehyde, phosphomolybdic acid or KMnO_4_ solution stains. Column chromatography was performed with silica gel (100–200 mesh) with the indicated solvent system.

### Synthetic procedure for amide and urea derivatives of benzoselenophene

#### Ethyl 5-aminobenzo[*b*]selenophene-2-carboxylate (2)

The synthetic procedure for compound 2 is described in our previous work.^21^

#### 5-(Dimethylamino)benzo[*b*]selenophene-2-carboxylic acid (3)

To a solution of ethyl 5-aminobenzo[*b*]selenophene-2-carboxylate (2) (200 mg, 0.75 mmol) in 5 mL CH_3_CN, 36.5% formaldehyde solution in H_2_O (1 mL) was slowly added at 0 °C. After 5 min stirring, NaBH_3_CN (9 mg, 0.15 mmol) and acetic acid (0.1 mL) were added and the reaction mixture was stirred continuously at room temperature for 24 h. After complete conversion of starting compound, the reaction mixture was diluted with CH_2_Cl_2_–H_2_O (10 mL, 1 : 1) mixture. The organic layer was separated and dried over the MgSO_4_ and filtered. The filtrate was concentrated under reduced pressure to provide the crude product, which was purified by silica column chromatography using 30% ethyl acetate in hexane as an eluent to afford the desired ethyl 5-(dimethylamino)benzo[*b*]selenophene-2-carboxylate as a brown solid (213 mg, 97%). ^1^H NMR (500.1 MHz, CDCl_3_) *δ* 8.19 (s, 1H), 7.69 (d, *J* = 8.9, 1H), 7.15 (s, 1H), 6.95 (d, *J* = 8.9, 1H), 4.37 (q, *J* = 7.1 Hz, 2H), 2.98 (s, 6H), 1.40 (t, *J* = 7.1 Hz, 3H); ^13^C NMR (125.7 MHz, CDCl_3_) *δ* 164.2, 149.1, 142.5, 136.5, 134.5, 132.1, 126.0, 115.5, 109.7, 61.5, 41.1 (2C), 14.4; HRMS (ESI): *m*/*z* calcd for (C_13_H_15_NO_2_Se) [M]^+^ 297.0268, found 297.0268. The obtained ester intermediate (200 mg, 0.68 mmol) was dissolved in 5 mL MeOH and then 5 mL 3 N NaOH solution was added. The mixture was stirred at room temperature for 24 h. After complete hydrolysis, the reaction mixture was concentrated under reduced pressure to obtain a crude residue which was acidified with 2 N HCl solution. The crude product was extracted with CH_2_Cl_2_ (3 × 10 mL), concentrated under reduced pressure and then purified by silica column chromatography using 5% MeOH in CH_2_Cl_2_ as an eluent to afford the desired product 3 as a brown solid (150 mg, 83%). ^1^H NMR (500.1 MHz, CD_3_OD) *δ* 8.40 (s, 2H), 8.29–8.27 (m, 1H), 7.78 (d, *J* = 8.6 Hz, 1H), 3.39 (s, 6H); ^13^C NMR (125.7 MHz, CD_3_OD) *δ* 164.5, 149.6, 144.6, 142.2, 140.1, 138.3, 133.9, 127.7, 117.3, 43.0 (2C); HRMS (ESI): *m*/*z* calcd for (C_11_H_12_NO_2_Se) [M + H]^+^ 270.0033, found 270.0034.

#### 5-(Methylsulfonamido)benzo[*b*]selenophene-2-carboxylic acid (4)

Compound 2 (130 mg, 0.48 mmol) was dissolved in dry CH_2_Cl_2_ (5 mL) and then pyridine (0.2 mL, 2.42 mmol) was added at 0 °C. The reaction mixture was stirred for 10 min, after that methanesulfonyl chloride (0.06 mL, 0.72 mmol) was added slowly under a N_2_ atmosphere. The reaction mixture was stirred at room temperature for 3 h. Upon completion of the reaction, the solvent was removed under reduced pressure, and the residue was purified by silica column chromatography using 20% ethyl acetate in hexane. The desired intermediate ethyl 5-(methylsulfonamido)benzo[*b*]selenophene-2-carboxylate was obtained as a yellow solid (150 mg, 89%). ^1^H NMR (500.1 MHz, CDCl_3_) *δ* 8.23 (s, 1H), 7.85 (d, *J* = 8.0 Hz, 1H), 7.81 (s, 1H), 7.29 (d, *J* = 8.0 Hz, 1H), 7.25 (s, 1H), 4.41–4.37 (m, 2H), 3.04 (s, 3H), 1.40 (m, 3H); ^13^C NMR (125.7 MHz, CDCl_3_) *δ* 162.7, 141.1, 139.8, 137.6, 133.4, 132.7, 126.0, 120.1, 118.3, 60.9, 38.2, 13.3. The obtained ester intermediate (128 mg, 0.37 mmol) was hydrolysed by a similar method for the synthesis of compound 3. The desired acid 4 was obtained as a yellow solid after purification by silica column chromatography using 5% MeOH in CH_2_Cl_2_ as an eluent (111 mg, 94%). ^1^H NMR (500.1 MHz, CD_3_OD) *δ* 8.20 (s, 1H), 7.93 (brs, 1H), 7.82 (s, 1H), 7.30 (brs, 1H), 2.95 (s, 3H); ^13^C NMR (125.7 MHz, CD_3_OD) *δ* 166.7, 143.7, 141.5, 140.3, 137.3, 135.2, 128.0, 122.3, 120.0, 39.4.

#### 5-(3-(Dimethylamino)propanamido)benzo[*b*]selenophene-2-carboxylic acid (5)

The mixture of 3-(dimethylamino)propanoic acid (103 mg, 0.67 mmol), *N*-(3-dimethylaminopropyl)-*N*′-ethylcarbodiimide hydrochloride EDCI (257 mg, 1.34 mmol) and DMAP (109 mg, 0.89 mmol) were dissolved in 5 mL dry DMF. The reaction mixture was stirred at 0 °C for 15 min and then the solution of compound 2 (120 mg, 0.45 mmol) in 0.5 mL DMF was slowly added under a N_2_ atmosphere. The reaction mixture was stirred continuously at room temperature for 17 h until complete conversion of starting material was confirmed by TLC. The reaction mixture was diluted with 10 mL water and extracted with CH_2_Cl_2_ (3 × 10 mL). The combined organic layers was washed with brine, dried over the MgSO_4_, filtered and concentrated under reduced pressure. The crude product was purified by silica column chromatography using 10% MeOH in CH_2_Cl_2_ as an eluent to provide the desired ethyl 5-(3-(dimethylamino)propanamido)benzo[*b*]selenophene-2-carboxylate as a brown solid (116 mg, 71%). ^1^H NMR (500.1 MHz, CDCl_3_) *δ* 8.25 (s, 1H), 8.23 (s, 1H), 7.77 (d, *J* = 8.6 Hz, 1H), 7.40 (d, *J* = 8.6 Hz, 1H), 4.37 (q, *J* = 7.1 Hz, 2H), 2.71 (t, *J* = 5.8 Hz, 2H), 2.56 (t, *J* = 5.7 Hz, 2H), 2.42 (s, 6H), 1.39 (t, *J* = 7.2 Hz, 3H); ^13^C NMR (125.7 MHz, CDCl_3_) *δ* 169.7, 162.9, 140.8, 137.6, 136.4, 135.4, 133.3, 124.9, 119.1, 116.8, 60.6, 54.1, 43.4 (2C), 32.3, 13.3; HRMS (ESI): *m*/*z* calcd for (C_16_H_21_N_2_O_3_Se) [M + H]^+^ 369.0717, found 369.0718. The obtained ester intermediate (100 mg, 0.27 mmol) was hydrolyzed by a similar method used for the synthesis of 3, the desired product 5 was obtained as a pale yellow solid after purification by silica column chromatography using 10% MeOH in CH_2_Cl_2_ as an eluent (80 mg, 88%). ^1^H NMR (500.1 MHz, CD_3_OD) *δ* 8.05 (s, 1H), 7.85 (s, 1H), 7.72 (d, *J* = 8.6 Hz, 1H), 7.38 (d, *J* = 8.6 Hz, 1H), 3.42 (t, *J* = 6.4 Hz, 2H), 2.91–2.88 (m, 8H); ^13^C NMR (125.7 MHz, CD_3_OD) *δ* 171.4, 168.0, 141.6, 139.8, 137.7, 136.6, 133.4, 126.4, 119.6, 117.1, 52.5, 42.1, 30.9; HRMS (ESI): *m*/*z* calcd for (C_14_H_17_N_2_O_3_Se) [M + H]^+^ 341.0404, found 341.0406.

#### 5-(3-(Methylthio)propanamido)benzo[*b*]selenophene-2-carboxylic acid (6)

The intermediate ethyl 5-(3-(methylthio)propanamido)benzo[*b*]selenophene-2-carboxylate was synthesized by using compound 2 (150 mg, 0.56 mmol) and 3-(methylthio)propanoic acid (101 mg, 0.84 mmol). EDCI (321 mg, 1.68 mmol) and DMAP (137 mg, 1.12 mmol) were used as coupling reagents. The reaction method was similar to that described for the synthesis of the ester intermediate 5 from the corresponding amine 2. The crude product was purified by silica column chromatography using 10% MeOH in CH_2_Cl_2_ as an eluent. The desired product was obtained as a brown solid (157 mg, 76%). ^1^H NMR (500.1 MHz, CDCl_3_) *δ* 8.22 (brs, 1H, NH), 8.16 (d, *J* = 2.0 Hz, 1H), 8.13 (s, 1H), 7.74 (d, *J* = 8.6 Hz, 1H), 7.40 (dd, *J* = 8.7, 2.1 Hz, 1H), 4.36 (q, *J* = 7.1 Hz, 2H), 2.88 (t, *J* = 7.1 Hz, 2H), 2.68 (t, *J* = 7.1 Hz, 2H), 2.14 (s, 3H), 1.38 (t, *J* = 7.2 Hz, 3H); ^13^C NMR (125.7 MHz, CDCl_3_) *δ* 170.1, 163.9, 141.7, 139.5, 137.7, 135.5, 134.1, 126.1, 120.3, 118.4, 61.8, 37.2, 29.9, 15.8, 14.4; HRMS (ESI): *m*/*z* calcd for (C_15_H_18_NO_3_SSe) [M + H]^+^ 372.0173, found 372.0172. Then, the obtained ester intermediate (150 mg, 0.36 mmol) was hydrolyzed by a similar method used for the synthesis of 3. Compound 6 was obtained as a yellow solid after purification by silica column chromatography using 5% MeOH in CH_2_Cl_2_ as an eluent (132 mg, 96%). ^1^H NMR (500.1 MHz, CD_3_OD) *δ* 8.22 (s, 1H), 8.14 (s, 1H), 7.86 (s, 1H), 7.48 (s, 1H), 2.84 (s, 2H), 2.70 (s, 2H), 2.14 (s, 3H); ^13^C NMR (125.7 MHz, CD_3_OD) *δ* 172.9, 167.7, 143.5, 140.7, 137.5, 134.4, 132.6, 127.2, 121.2, 119.3, 38.0, 30.8, 15.6; HRMS (ESI): *m*/*z* calcd for (C_13_H_14_NO_3_SSe) [M + H]^+^ 343.9860, found 343.9859.

#### 5-(4-Acetamido-1-methyl-1*H*-pyrrole-2-carboxamido)benzo[*b*]selenophene-2-carboxylic acid (7)

The intermediate ethyl 5-(4-((*tert*-butoxycarbonyl)amino)-1-methyl-1*H*-pyrrole-2-carboxamido)benzo[*b*]selenophene-2-carboxylate was synthesized by using 2 (200 mg, 0.75 mmol) and 4-((*tert*-butoxycarbonyl)amino)-1-methyl-1*H*-pyrrole-2-carboxylic acid (269 mg, 1.12 mmol). The reaction method was similar to the described procedure for the synthesis of ester intermediate of 5 from amine 2. EDCI (571 mg, 2.98 mmol) and DMAP (364 mg, 2.98 mmol) were used as coupling reagents. The desired product was obtained as a yellow solid after purification by silica column chromatography using 5% MeOH in CH_2_Cl_2_ as an eluent (245 mg, 68%).^1^H NMR (500.1 MHz, CDCl_3_) *δ* 8.16 (s, 1H), 8.08 (s, 1H), 8.01 (s, 1H), 7.63 (d, *J* = 7.6 Hz, 1H), 7.36 (d, *J* = 6.5 Hz, 1H), 6.82 (s, 2H), 6.68 (s, 1H), 4.30 (q, *J* = 7.2 Hz, 2H), 3.77 (s, 3H), 1.44 (s, 9H), 1.33 (t, *J* = 7.1 Hz, 3H); ^13^C NMR (125.7 MHz, CDCl_3_) *δ* 162.6, 158.6, 152.3, 140.2, 137.5, 135.8, 134.5, 132.9, 124.5, 121.7, 120.5, 119.2, 117.9, 116.9, 103.3, 78.7, 60.4, 35.3, 27.0 (3C), 12.9; HRMS (ESI): *m*/*z* calcd for (C_22_H_26_N_3_O_5_Se) [M + H]^+^ 492.1038, found 492.1039. The obtained intermediate (230 mg, 0.47 mmol) was dissolved in 5 mL dry CH_2_Cl_2_, and then 20% TFA in CH_2_Cl_2_ was added slowly at 0 °C. The reaction mixture was stirred continuously at room temperature for 3 h. After completion the reaction, the solvent was removed under reduced pressure. The residue was dissolved in 5 mL dry CH_2_Cl_2,_ and then acetyl chloride (110 mg, 1.41 mmol) and TEA (237 mg, 2.34 mmol) were added at 0 °C. The reaction mixture was stirred at room temperature for 5 h until complete conversion of starting material was confirmed by TLC. The reaction mixture was diluted with 10 mL water and extracted with CH_2_Cl_2_ (3 × 10 mL). The combined organic layers was washed with brine, dried over the MgSO_4_, filtered and concentrated under reduced pressure. The crude product was purified by silica column chromatography using 5% MeOH in CH_2_Cl_2_ as an eluent. The desired ethyl 5-(4-acetamido-1-methyl-1*H*-pyrrole-2-carboxamido)benzo[*b*]selenophene-2-carboxylate was obtained as a yellow solid (90 mg, 45%). ^1^H NMR (500.1 MHz, DMSO-d_6_) *δ* 9.94 (s, 1H), 9.80 (s, 1H), 8.42 (s, 1H), 8.34 (s, 1H), 8.02 (d, *J* = 8.7 Hz, 1H), 7.72 (d, *J* = 8.8 Hz, 1H), 7.19 (s, 1H), 6.98 (s, 1H), 4.32 (q, *J* = 7.1 Hz, 2H), 3.84 (s, 3H), 1.98 (s, 3H), 1.32 (t, *J* = 7.0 Hz, 3H); ^13^C NMR (125.7 MHz, DMSO-d_6_) *δ* 166.8, 163.3, 159.9, 141.3, 137.5, 137.1, 136.5, 134.5, 126.1, 122.6, 122.2, 121.2, 119.0, 118.4, 105.0, 61.5, 36.1, 23.0, 14.1; HRMS (ESI): *m*/*z* calcd for (C_19_H_20_N_3_O_4_Se) [M + H]^+^ 434.0619, found 434.0620. The obtained ester intermediate (90 mg, 0.21 mmol) was hydrolyzed by a similar method used for the synthesis of 3. The desired acid 7 was obtained as an off-white solid after purification by silica column chromatography using 10% MeOH in CH_2_Cl_2_ as an eluent (76 mg, 91%). ^1^H NMR (500.1 MHz, DMSO-d_6_) *δ* 10.10 (s, 1H), 10.02 (s, 1H), 8.43 (s, 1H), 8.23 (s, 1H), 7.98 (s, 1H), 7.74 (s, 1H), 7.20 (s, 1H), 7.07 (s, 1H), 3.82 (s, 3H), 1.98 (s, 3H); ^13^C NMR (125.7 MHz, DMSO-d_6_) *δ* 166.7, 164.6, 159.9, 141.5, 138.5, 137.4, 137.1, 133.9, 126.1, 122.5, 122.3, 120.9, 119.1, 118.2, 105.3, 36.3, 23.1; HRMS (ESI): *m*/*z* calcd for (C_17_H_16_N_3_O_4_Se) [M + H]^+^ 406.0306, found 406.0307.

#### Ethyl 5-amino-6-methoxybenzo[*b*]selenophene-2-carboxylate (9)

To a solution of ethyl 6-methoxy-5-nitrobenzo[*b*]selenophene-2-carboxylate^24^ (1.8 g, 4.36 mmol) in 25 mL dry ethyl acetate, 10% Pd/C was added under a N_2_ atmosphere. The reaction mixture was stirred under a H_2_ atmosphere for 6 h. On complete conversion, the mixture was filtered through a Celite pad followed by washing with ethyl acetate (3 × 25 mL). The filtrate was concentrated under reduced pressure to obtain the crude product, which was purified by silica column chromatography using 30% ethyl acetate in hexane as an eluent to provide the desired product 9 as an oily liquid (1.54 g, 94%) ^1^H NMR (500.1 MHz, CDCl_3_) *δ* 8.05 (s, 1H), 7.18 (s, 1H), 7.10 (s, 1H), 4.34 (q, *J* = 7.1 Hz, 2H), 3.87 (s, 3H), 3.82 (brs, 2H), 1.37 (t, *J* = 7.1 Hz, 3H); ^13^C NMR (125.7 MHz, CDCl_3_) *δ* 164.2, 149.1, 135.6, 135.2, 134.8, 133.8, 133.4, 110.9, 106.0, 61.3, 55.8, 14.4.

#### Ethyl 5-acetamido-6-methoxybenzo[*b*]selenophene-2-carboxylate (10)

Pyridine (1.49 mL, 18.5 mmol) was added to the solution of 9 (1.1 g, 3.69 mmol) in 10 mL dry CH_2_Cl_2_. The reaction mixture was stirred for 15 min at room temperature, after that acetic anhydride (0.5 mL, 3.87 mmol) was added slowly under a N_2_ atmosphere. The reaction mixture was stirred continuously at room temperature for 8 h. The reaction mixture was quenched with 10 mL water and desired product was extracted with CH_2_Cl_2_ (3 × 10 mL). The combined organic layer was washed with brine and dried over MgSO_4_, filtered and concentrated under reduced pressure. The crude product was purified by silica column chromatography using 50% ethyl acetate in hexane as an eluent to afford the desired 10 as a brown solid (1.13 g, 90%). ^1^H NMR (500.1 MHz, CDCl_3_) *δ* 8.84 (s, 1H), 8.16 (s, 1H), 7.82 (s, 1H), 7.27 (s, 1H), 4.34 (q, *J* = 7.1 Hz, 2H), 3.92 (s, 3H), 2.21 (s, 3H), 1.37 (t, *J* = 7.1 Hz, 3H); ^13^C NMR (125.7 MHz, CDCl_3_) *δ* 168.3, 163.9, 148.3, 139.5, 135.0, 134.6, 134.3, 126.7, 117.3, 105.9, 61.5, 56.1, 25.0, 14.4. HRMS (ESI): *m*/*z* calcd for (C_14_H_15_NNaO_4_Se) [M + Na]^+^ 364.0064, found 364.0058.

#### 5-Acetamido-6-methoxybenzo[*b*]selenophene-2-carboxylic acid (11)

Compound 10 (200 mg, 0.59 mmol) was hydrolysed by similar method used for the synthesis of 3. The desired acid 11 was obtained as a yellow solid after purification by silica column chromatography using 5% MeOH in CH_2_Cl_2_ as an eluent (170 mg, 93%). ^1^H NMR (500.1 MHz, DMSO-d_6_) *δ* 9.28 (s, 1H), 8.49 (s, 1H), 8.18 (s, 1H), 7.78 (s, 1H), 3.90 (s, 3H), 2.11 (s, 3H); ^13^C NMR (125.7 MHz, DMSO-d_6_) *δ* 168.9, 164.9, 150.2, 139.8, 135.3, 134.4 (2C), 126.6, 119.8, 107.7, 56.1, 24.0.

#### Ethyl 5-acetamido-6-hydroxybenzo[*b*]selenophene-2-carboxylate (12)

Compound 10 (0.8 g, 2.35 mmol) was dissolved in 10 mL anhydrous CH_2_Cl_2_, and then tetra *n*-butylammonium iodide (2.17 g, 5.87 mmol) was added under a N_2_ atmosphere at −78 °C. After 10 min stirring, 5.9 mL of BCl_3_ (1 M CH_2_Cl_2_ solution, 5.87 mmol) was slowly added, and then the reaction mixture was stirred for 2 h at 0 °C. The reaction mixture was quenched with 25 mL ice water and desired product was extracted with CH_2_Cl_2_ (3 × 50 mL). The combined organic layer was washed with brine, dried over MgSO_4_, filtered and concentrated under reduced pressure to obtain a crude product 0.620 g, which was used without purification for next step reaction. HRMS (ESI): *m*/*z* calcd for (C_13_H_13_NNaO_4_Se) [M + Na]^+^ 349.9907, found 349.9902.

#### 5-Acetamido-6-(2-(dimethylamino)ethoxy)benzo[*b*]selenophene-2-carboxylic acid (13)

Compound 12 (300 mg, 0.92 mmol) and potassium carbonate (636 mg, 4.6 mmol) were mixed in 30 mL acetone. 2-Chloro-*N*,*N*-dimethylethanamine hydrochloride (397 mg, 1.38 mmol) was slowly added to this solution with stirring at 60 °C for 4 h. After complete conversion of starting material, the reaction mixture was cooled to room temperature, and then quenched with 50 mL water. The desired product was extracted with CH_2_Cl_2_ (3 × 50 mL), washed with brine and concentrated under reduced pressure. The crude residue was purified by silica column chromatography using 50% ethyl acetate in hexane as an eluent to provide the intermediate, ethyl 5-acetamido-6-(2-(dimethylamino)ethoxy)benzo[*b*]selenophene-2-carboxylate (292 mg, 80%). ^1^H NMR (500.1 MHz, CDCl_3_) *δ* 9.08 (s, 1H), 8.82 (s, 1H), 8.15 (s, 1H), 7.37 (s, 1H), 4.33 (q, *J* = 7.1 Hz, 2H), 4.15 (m, 2H), 2.67 (m, 2H), 2.35 (s, 6H), 2.16 (s, 3H), 1.34 (t, *J* = 7.1 Hz, 3H). The obtained ester intermediate (280 mg, 0.7 mmol) was hydrolysed by similar method using for synthesis of 3. The desired acid 13 was obtained as a yellow solid after purification by silica column chromatography using 5% MeOH in CH_2_Cl_2_ as an eluent (229 mg, 88%). ^1^H NMR (500.1 MHz, CD_3_OD) *δ* 8.26 (s, 1H), 7.80 (s, 1H), 7.45 (s, 1H), 4.33 (m, 2H), 3.39 (m, 2H), 2.82 (s, 6H), 2.20 (s, 3H); ^13^C NMR (125.7 MHz, CD_3_OD) *δ* 172.2, 171.2, 149.5, 146.3, 141.7, 137.6, 131.2, 126.8, 121.4, 109.2, 64.9, 58.1, 44.5, 24.0. HRMS (ESI): *m*/*z* calcd for (C_15_H_18_N_2_O_4_Se) [M]^+^ 369.2744, found 369.3512.

#### 6-(2,5,8,11-Tetraoxatridecan-13-yloxy)-5-acetamidobenzo[*b*]selenophene-2-carboxylic acid (14)

The desired intermediate ethyl 6-(2,5,8,11-tetraoxatridecan-13-yloxy)-5-acetamidobenzo[*b*]selenophene-2-carboxylate was synthesized by using 12 (200 mg, 0.61 mmol), 2,5,8,11-tetraoxatridecan-13-yl 4-methylbenzenesulfonate (667 mg, 1.84 mmol) and K_2_CO_3_ (424 mg, 3.07 mmol). The reaction method was similar to that described for the synthesis of the ester intermediate of 13 from the corresponding amine 12. The crude product was purified by silica column chromatography using 5% MeOH in CH_2_Cl_2_ as an eluent (294 mg, 93%). ^1^H NMR (500.1 MHz, CDCl_3_) *δ* 8.87 (s, 1H), 8.22 (s, 1H), 8.18 (s, 1H), 7.36 (s, 1H), 4.35 (q, *J* = 7.1 Hz, 2H), 4.25 (t, *J* = 4.4 Hz, 2H), 3.90 (t, *J* = 4.4 Hz, 2H), 3.74–3.59 (m, 10H), 3.51 (t, *J* = 4.6 Hz, 2H), 3.34 (s, 3H), 2.22 (s, 3H), 1.38 (t, *J* = 4.6 Hz, 3H); ^13^C NMR (125.7 MHz, CDCl_3_) *δ* 168.7, 163.9, 147.8, 139.3, 136.6, 135.6, 134.6, 127.5, 118.0, 108.5, 71.9, 70.7, 70.6, 70.5 (2C), 69.3 (2C), 69.2, 61.5, 59.0, 24.8, 14.4. The obtained ester intermediate (250 mg, 0.48 mmol) was hydrolysed by similar method used for the synthesis of 3. The desired acid 14 was obtained as an off-white solid after purification by silica column chromatography using 5% MeOH in CH_2_Cl_2_ as an eluent (210 mg, 89%). ^13^C NMR (125.7 MHz, DMSO-d_6_) *δ* 167.5, 166.1, 147.4, 137.7, 134.2, 129.3, 127.0, 125.2, 117.9, 108.3, 69.9, 68.6, 68.4 (3C), 68.2, 67.4, 67.3, 56.6, 22.6.

### Synthesis of the intermediate (16)

A solution of 9 (5.0 g, 13.17 mmol) in anhydrous THF (350 mL) was cooled to −78 °C, then treated with catalytic amount H_2_SO_4_ (85 μL) in THF (10 mL). After 15 min stirring, a solution of NIS (3.55 g, 15.82 mmol) in THF (20 mL) was added and the reaction mixture was stirred at −78 °C for 2 h, and then at room temperature for 30 min. The progress of the reaction was monitored by TLC. After complete conversion of starting material, NaH (60% dispersion in mineral oil, 4.3 g, 105.42 mmol) was added in portion under N_2_ atmosphere at 0 °C and then stirred the reaction mixture at room temperature for 30 min. (*S*)-Glycidyl nosylate (4.10 g, 15.82 mmol) was added under N_2_ atmosphere and the mixture was stirred for 3 to 5 h at room temperature. On complete conversion of intermediate, 3 M solution of EtMgBr in diethyl ether (13.15 mL, 39.53 mmol) was added slowly and stirred continuously for 2 h. The reaction mixture was quenched with saturated NH_4_Cl at 0 °C, and then extracted with ethyl acetate (3 × 200 mL). The combine organic layer was washed with aqueous NaCl and dried over Na_2_SO_4_, filtered and concentrated under reduced pressure to get crude residue, which was purified by flash column chromatography on silica gel using 40% ethyl acetate in hexane as an eluent to provide the desired product 16 (4.74 g, 82%).

### General procedures for the synthesis of *seco*-MCBI derivatives (18a–x)

To 17 (30 mg, 0.09 mmol) in a round bottom flask, 4 mL saturated solution of HCl in ethyl acetate was added at −78 °C. The reaction mixture was stirred at the −78 °C for 30 min and then room temperature for 1 h. After salt formation was observed on TLC, ethyl acetate was evaporated under nitrogen flow and then completely dried under high vacuum for 1 h. The resulting residue was dissolved in anhydrous DMF (0.2 mL) and added to the mixture of acid (1.1 eq.) and EDCI (52 mg, 0.27 mmol) in anhydrous DMF (0.5 mL) at 0 °C. The reaction mixture was stirred at 0 °C for 3 h and then at room temperature for 5 h. After completion the reaction, the reaction mixture was diluted with water and extracted with ethyl acetate (3 × 15 mL). The combined organic layer was washed with brine, dried over MgSO_4_, filtered and concentrated. The crude product was purified by column chromatography to afford the desired product.

### Spectral characteristics of 18a–x

#### (*S*)-(1-(Chloromethyl)-5-hydroxy-8-methoxy-1,2-dihydro-3*H*-benzo[*e*]indol-3-yl)(selenopheno[3,2-*b*]thiophen-5-yl)methanone (18a)

Pale yellow solid, 68%, ^1^H NMR (500.1 MHz, acetone-d_6_) *δ* 9.34 (brs, 1H), 8.61 (dd, *J* = 4.6, 1.3 Hz, 1H), 8.33 (dd, *J* = 8.0, 1.3 Hz, 1H), 8.16 (m, 1H), 7.97 (s, 1H), 7.75 (brs, 1H), 7.49 (dd, *J* = 8.0, 4.6, 1H), 7.19 (d, *J* = 2.4 Hz, 1H), 7.04 (dd, *J* = 9.2, 2.4 Hz, 1H), 4.72 (t, *J* = 9.6 Hz, 1H), 4.64 (d, *J* = 10.8 Hz, 1H), 4.20–4.16 (m, 1H), 4.05 (dd, *J* = 11.2, 3.3 Hz, 1H), 3.95 (s, 3H), 3.83 (dd, *J* = 11.1, 8.5 Hz, 1H); ^13^C NMR (125.7 MHz, acetone-d_6_) *δ* 166.3, 163.1, 162.8, 160.2, 155.3, 149.0, 143.5, 137.2, 135.3, 132.8, 128.3, 126.0, 121.4, 118.7, 116.7, 116.3, 102.3, 99.5, 56.5, 55.8, 47.4, 42.8; HRMS (FAB): *m*/*z* calcd for (C_22_H_18_ClN_2_O_3_Se) [M + H]^+^ 473.0171, found 473.0166.

#### (*S*)-(1-(Chloromethyl)-5-hydroxy-8-methoxy-1,2-dihydro-3*H*-benzo[*e*]indol-3 yl)(selenopheno[3,2-*b*]thiophen-5-yl)methanone (18b)

Pale yellow solid, 48%, ^1^H NMR (500.1 MHz, CDCl_3_) *δ* 8.91 (s, 1H), 8.20 (d, *J* = 9.1 Hz, 1H), 8.05 (s, 1H), 7.95 (s, 1H), 7.33–7.30 (m, 2H), 7.02 (dd, *J* = 9.1, 1.7 Hz, 1H), 6.85 (s, 1H), 4.64 (d, *J* = 10.5 Hz, 1H), 4.58–4.54 (m, 1H), 3.97–3.95 (m, 2H), 3.91 (s, 3H), 3.43 (t, *J* = 10.8 Hz, 1H); ^13^C NMR (125.7 MHz, CDCl_3_) *δ* 164.4, 155.1, 149.9, 148.6, 141.9, 138.9, 136.2, 134.8, 131.4, 130.9, 125.9, 118.3, 115.7, 115.1, 106.5, 101.1, 99.2, 56.3, 55.5, 45.7, 43.0.

#### (*S*)-(1-(Chloromethyl)-5-hydroxy-8-methoxy-1,2-dihydro-3*H*-benzo[*e*]indol-3-yl)(thieno[3,2-*b*]thiophen-2-yl)methanone (18c)

Yellow solid, 46%, ^1^H NMR (500.1 MHz, CDCl_3_ + CD_3_OD) ^1^H NMR (500.1 MHz, CDCl_3_ + CD_3_OD) *δ* 8.94 (s, 1H), 8.10 (d, *J* = 9.1 Hz, 1H), 7.85 (s, 1H), 7.57 (d, *J* = 5.3 Hz, 1H), 7.30 (d, *J* = 5.3 Hz, 1H), 6.96–6.93 (m, 2H), 4.59–4.58 (m, 2H), 3.98–3.97 (m, 1H), 3.90 (m, 1H), 3.88 (s, 3H), 3.52–3.48 (m, 1H); ^13^C NMR (125.7 MHz, CDCl_3_ + CD_3_OD) *δ* 162.7, 159.6, 155.3, 143.1, 142.4, 140.3, 139.5, 131.9, 131.7, 125.9, 123.5, 120.0, 118.7, 116.1, 115.5, 101.5, 98.7, 56.6, 55.6, 46.0, 43.1; HRMS (FAB): *m*/*z* calcd for (C_21_H_17_ClNO_3_S_2_) [M + H]^+^ 430.0338, found 430.0333.

#### (*S*)-(1-(Chloromethyl)-5-hydroxy-8-methoxy-1,2-dihydro-3*H*-benzo[*e*]indol-3-yl)(4*H*-thieno[3,2-*b*]pyrrol-5-yl)methanone (18d)

Pale yellow solid, 51%, ^1^H NMR (500.1 MHz, acetone-d_6_) *δ* 10.85 (s, 1H), 8.99 (s, 1H), 8.01 (d, *J* = 9.2 Hz, 1H), 7.79 (s, 1H), 7.30 (d, *J* = 5.3 Hz, 1H), 7.09–7.06 (m, 2H), 6.96 (d, *J* = 5.2 Hz, 1H), 6.88 (dd, *J* = 9.2, 2.4 Hz, 1H), 4.64–4.57 (m, 2H), 4.12–4.07 (m, 1H), 3.94 (dd, *J* = 11.2, 3.3 Hz, 1H), 3.83 (s, 3H), 3.65 (dd, *J* = 11.2, 8.6 Hz, 1H); ^13^C NMR (125.7 MHz, acetone-d_6_) *δ* 160.1, 144.5, 142.0, 132.8, 131.9, 131.5, 129.2, 125.9, 125.4, 116.3 (2C), 115.4, 112.6, 106.0, 102.1, 99.8, 99.7, 55.8, 55.7, 47.6, 43.2; HRMS (FAB): *m*/*z* calcd for (C_21_H_18_ClN_2_O_3_S) [M + H]^+^ 413.0727, found 413.0722.

#### (S)-(1-(Chloromethyl)-5-hydroxy-8-methoxy-1,2-dihydro-3*H*-benzo[*e*]indol-3-yl)(selenopheno[3,2-*b*]furan-5-yl)methanone (18e)

Yellow solid, 58%, ^1^H NMR (500.1 MHz, CDCl_3_) *δ* 8.16 (d, *J* = 9.2 Hz, 1H), 7.94 (s, 1H), 7.79 (s, 1H), 7.67 (d, *J* = 1.9 Hz, 1H), 7.08–7.05 (m, 1H), 6.92 (d, *J* = 2.3 Hz, 1H), 6.89 (m, *J* = 1.8 Hz, 1H), 4.68 (d, *J* = 9.4 Hz, 1H), 4.67 (t, *J* = 9.4 Hz, 1H), 4.03 (m, 1H), 3.95 (s, 3H), 3.94 (m, 1H), 3.49–3.44 (m, 1H); ^13^C NMR (125.7 MHz, CDCl_3_) *δ* 161.6, 158.7, 157.4, 153.8, 147.3, 144.0, 142.6, 131.4, 126.1, 124.5, 117.2, 115.7, 115.1, 114.4, 109.1, 100.8, 98.3, 55.0, 54.3, 46.1, 41.6; HRMS (FAB): *m*/*z* calcd for (C_21_H_17_ClNO_4_Se) [M + H]^+^ 462.0011, found 462.0005.

#### (*S*)-(1-(Chloromethyl)-5-hydroxy-8-methoxy-1,2-dihydro-3*H*-benzo[*e*]indol-3-yl)(4*H*-furo[3,2-*b*]pyrrol-5-yl)methanone (18f)

Pale yellow solid, 61%, ^1^H NMR (500.1 MHz, acetone-d_6_ + CD_3_OD) *δ* 7.99 (d, *J* = 9.2 Hz, 1H), 7.65 (s, 1H), 7.54 (d, *J* = 2.0 Hz, 1H), 7.00 (d, *J* = 2.3 Hz, 1H), 6.87 (dd, *J* = 9.2, 2.3 Hz, 1H), 6.75 (s, 1H), 6.48 (d, *J* = 1.6, 1H), 4.57–4.50 (m, 2H), 4.06–4.02 (m, 1H), 3.91 (m, 1H), 3.83 (s, 3H), 3.61 (dd, *J* = 11.2, 8.6 Hz, 1H); ^13^C NMR (125.7 MHz, acetone-d_6_ + CD_3_OD) *δ* 160.6, 158.8, 154.1, 148.1 (2C), 143.0, 131.5, 127.7, 126.6, 124.7, 117.2, 114.9, 114.1, 100.7, 98.6, 98.1, 94.4, 54.8, 54.3, 46.1, 41.8; HRMS (FAB): *m*/*z* calcd for (C_21_H_17_ClN_2_NaO_4_) [M + Na]^+^ 419.0775, found 469.0769.

#### (*S*)-(1-(Chloromethyl)-5-hydroxy-8-methoxy-1,2-dihydro-3*H*-benzo[*e*]indol-3-yl)(thieno[2,3-*b*]quinolin-2-yl)methanone (18g)

Yellow solid, 30%, ^1^H NMR (500.1 MHz, acetone-d_6_ + CD_3_OD) *δ* 8.85 (s, 1H), 8.05–8.00 (m, 4H), 7.76 (m, 1H), 7.54 (t, *J* = 7.4 Hz, 2H), 7.04 (d, *J* = 2.3 Hz, 1H), 6.92 (dd, *J* = 9.2, 2.4 Hz, 1H), 4.67 (m, 1H), 4.56 (d, *J* = 10.5 Hz, 1H), 4.05 (m, 1H), 3.92 (m, 1H), 3.84 (s, 3H), 3.71 (dd, *J* = 11.1, 8.3 Hz, 1H); HRMS (FAB): *m*/*z* calcd for (C_26_H_20_ClN_2_O_3_S) [M + H]^+^ 475.0883, found 475.0879.

#### (*S*)-(1-(Chloromethyl)-5-hydroxy-8-methoxy-1,2-dihydro-3*H*-benzo[*e*]indol-3-yl)(5-methoxybenzo[*b*]selenophen-2-yl)methanone (18h)

Pale yellow solid, 54%, ^1^H NMR (500.1 MHz, acetone-d_6_) *δ* 9.26 (brs, 1H), 8.02 (s, 1H), 8.01 (d, *J* = 9.4 Hz, 1H), 7.80 (d, *J* = 8.8 Hz, 1H), 7.62 (brs, 1H), 7.40 (d, *J* = 2.5 Hz, 1H), 7.04 (d, *J* = 2.4 Hz, 1H), 6.94–6.92 (m, 1H), 6.90–6.88 (m, 1H), 4.56 (t, *J* = 10.6 Hz, 1H), 4.49 (dd, *J* = 10.8, 1.6 Hz, 1H), 4.04–4.00 (m, 1H), 3.91 (dd, *J* = 11.2, 3.3 Hz, 1H), 3.81 (s, 3H), 3.74 (s, 3H), 3.67 (dd, *J* = 11.1, 8.5 Hz, 1H); ^13^C NMR (125.7 MHz, acetone-d_6_) *δ* 163.4, 160.1, 159.1, 155.3, 145.5, 144.1, 143.7, 134.8, 132.7, 130.5, 127.1, 126.0, 118.6, 117.3, 116.5, 116.1, 110.2, 102.2, 99.5, 56.6, 55.8, 55.7, 47.4, 42.8; HRMS (FAB): *m*/*z* calcd for (C_24_H_21_ClNO_4_Se) [M + H]^+^ 502.0324, found 502.0319.

#### (*S*)-(1-(Chloromethyl)-5-hydroxy-8-methoxy-1,2-dihydro-3*H*-benzo[*e*]indol-3-yl)(6-methoxybenzo[*b*]selenophen-2-yl)methanone (18i)

Yellow solid, 51%, ^1^H NMR (500.1 MHz, CDCl_3_ + acetone-d_6_) *δ* 9.00 (s, 1H), 8.10 (d, *J* = 9.1 Hz, 1H), 7.79 (s, 1H), 7.73 (d, *J* = 8.8 Hz, 1H), 7.60 (brs, 1H), 7.41 (s, 1H), 6.97–6.93 (m, 3H), 4.59–4.53 (m, 2H), 3.98–3.95 (m, 1H), 3.91–3.88 (m, 4H), 3.84 (s, 3H), 3.50 (t, *J* = 10.4 Hz, 1H); ^13^C NMR (125.7 MHz, CDCl_3_ + acetone-d_6_) *δ* 162.6, 158.7, 158.5, 154.1, 144.0, 142.1, 139.5, 135.2, 131.2, 129.2, 127.4, 125.0, 117.4, 115.1, 114.5 (2C), 107.6, 100.7, 98.3, 55.4, 55.0, 54.8, 45.4, 42.1; HRMS (FAB): *m*/*z* calcd for (C_24_H_21_ClNO_4_Se) [M + H]^+^ 502.0324, found 502.0319.

#### (*S*)-(1-(Chloromethyl)-5-hydroxy-8-methoxy-1,2-dihydro-3*H*-benzo[*e*]indol-3-yl)(7-methoxybenzo[*b*]selenophen-2-yl)methanone (18j)

Yellow solid, 47%, ^1^H NMR (500.1 MHz, acetone-d_6_) *δ* 9.20 (s, 1H), 8.24 (s, 1H), 8.15 (d, *J* = 9.2 Hz, 1H), 7.74 (brs, 1H), 7.60 (d, *J* = 7.9 Hz, 1H), 7.45 (t, *J* = 7.9 Hz, 1H), 7.20 (d, *J* = 2.4 Hz, 1H), 7.03 (dd, *J* = 9.2, 2.4 Hz, 1H), 7.00 (d, *J* = 7.9 Hz, 1H), 4.74 (t, *J* = 9.7 Hz, 1H), 4.64 (dd, *J* = 10.9, 1.5 Hz, 1H), 4.19 (m, 1H), 4.07–4.04 (m, 1H), 4.04 (s, 3H), 4.00 (s, 3H), 3.82 (dd, *J* = 11.1, 8.5 Hz, 1H); ^13^C NMR (125.7 MHz, acetone-d_6_ + CD_3_OD) *δ* 163.9, 160.3, 157.3, 155.5, 144.5, 143.6, 132.8, 131.8, 131.2, 127.9, 126.1, 120.6, 118.9, 116.7, 116.3, 107.1, 102.2, 99.3, 97.3, 56.9, 56.3, 55.7, 47.4, 42.9; HRMS (FAB): *m*/*z* calcd for (C_24_H_21_ClNO_4_Se) [M + H]^+^ 502.0324, found 502.0319.

#### (*S*)-(1-(Chloromethyl)-5-hydroxy-8-methoxy-1,2-dihydro-3*H*-benzo[*e*]indol-3-yl)(5,6-dimethoxybenzo[*b*]selenophen-2-yl)methanone (18k)

Yellow solid, 44% ^1^H NMR (500.1 MHz, acetone-d_6_) *δ* 9.24 (s, 1H), 8.15–8.13 (m, 2H), 7.75 (s, 1H), 7.63 (s, 1H), 7.51 (s, 1H), 7.19 (d, *J* = 2.4 Hz, 1H), 7.02 (dd, *J* = 9.2, 2.5 Hz, 1H), 4.73 (t, *J* = 10.7 Hz, 1H), 4.66 (dd, *J* = 10.8, 1.8 Hz, 1H), 4.20–4.15 (m, 1H), 4.07–4.04 (m, 1H), 3.95 (s, 3H), 3.92 (s, 3H), 3.88 (s, 3H), 3.79 (dd, *J* = 11.1, 8.7 Hz, 1H); ^13^C NMR (125.7 MHz, acetone-d_6_) *δ* 163.4, 160.1, 155.2, 151.2, 149.9, 144.0, 142.3, 136.5, 136.4, 132.8, 130.8, 126.0, 118.6, 116.5, 115.9, 109.5, 108.1, 102.2, 99.7, 56.6, 56.3, 56.2, 55.7, 47.4, 43.0; LCMS (ESI): *m*/*z* calcd for (C_25_H_23_ClNO_5_Se) [M + H]^+^ 532.04, found 532.12.

#### (*S*)-(1-(Chloromethyl)-5-hydroxy-8-methoxy-1,2-dihydro-3*H*-benzo[*e*]indol-3-yl)(5-nitrobenzo[*b*]selenophen-2-yl)methanone (18l)

Yellow solid, 70%, ^1^H NMR (500.1 MHz, DMSO-d_6_) *δ* 10.38 (s, 1H), 8.92 (d, *J* = 2.0 Hz, 1H), 8.54 (s, 1H), 8.44 (d, *J* = 8.9 Hz, 1H), 8.21 (dd, *J* = 8.9, 2.1 Hz, 1H), 8.02 (d, *J* = 9.2 Hz, 1H), 7.74 (brs, 1H), 7.12 (d, *J* = 1.8 Hz, 1H), 7.01 (dd, *J* = 9.2, 2.0 Hz, 1H), 4.71 (t, *J* = 10.4 Hz, 1H), 4.46 (d, *J* = 10.8 Hz, 1H), 4.21 (m, 1H), 4.02 (dd, *J* = 11.1, 2.7 Hz, 1H), 3.91 (m, 4H); ^13^C NMR (125.7 MHz, DMSO-d_6_) *δ* 161.5, 158.5, 154.4, 149.0, 147.4, 145.6, 142.2, 131.3, 129.8, 127.3, 124.9, 122.4 (2C), 119.7, 117.4, 115.6, 114.8, 101.7, 98.1, 55.3 (2C), 47.4, 40.9; HRMS (FAB): *m*/*z* calcd for (C_23_H_18_ClN_2_O_5_Se) [M + H]^+^ 517.0069, found 517.0063.

#### (*S*)-(1-(Chloromethyl)-5-hydroxy-8-methoxy-1,2-dihydro-3*H*-benzo[*e*]indol-3-yl)(5-(dimethylamino)benzo[*b*]selenophen-2-yl)methanone (18m)

Yellow solid, 63%, ^1^H NMR (500.1 MHz, acetone-d_6_) *δ* 9.30 (s, 1H), 8.14 (d, *J* = 9.2 Hz, 1H), 8.09 (s, 1H), 7.83 (d, *J* = 8.9 Hz, 1H), 7.74 (brs, 1H), 7.29 (s, 1H), 7.18 (s, 1H), 7.02 (d, 9.0 Hz, 2H), 4.69 (t, *J* = 9.6 Hz, 1H), 4.62 (d, *J* = 10.8 Hz, 1H), 4.17 (t, *J* = 8.5 Hz, 1H), 4.04 (dd, *J* = 11.2, 2.7 Hz, 1H), 3.95 (s, 3H), 3.82–3.78 (m, 1H), 2.99 (s, 6H); ^13^C NMR (500.1 MHz, CDCl_3_) *δ* 165.0, 159.3, 155.2, 149.4, 142.7, 142.0, 140.8, 131.5, 130.9 (2C), 126.1, 125.7, 118.5, 115.7, 115.3, 115.2, 109.8, 101.4, 99.3, 56.6, 55.5, 45.8, 42.9, 41.3 (2C); HRMS (FAB): *m*/*z* calcd for (C_25_H_24_ClN_2_O_3_Se) [M + H]^+^ 515.0641, found 515.0634.

#### (*S*)-*N*-(2-(1-(Chloromethyl)-5-hydroxy-8-methoxy-2,3-dihydro-1*H*-benzo[*e*]indole-3-carbonyl)benzo[*b*]selenophen-5-yl)acetamide (18n)

Pale yellow solid, 54%, ^1^H NMR (500.1 MHz, acetone-d_6_ + CD_3_OD) *δ* 8.35 (s, 1H), 8.12 (s, 1H), 8.07 (d, *J* = 9.2 Hz, 1H), 7.92 (d, *J* = 8.7 Hz, 1H), 7.59 (brs, 1H), 7.45 (d, *J* = 8.6 Hz, 1H), 7.08 (d, *J* = 1.9 Hz, 1H), 6.97 (dd, *J* = 9.2, 2.1 Hz, 1H), 4.67 (t, *J* = 9.6 Hz, 1H), 4.56 (d, *J* = 10.9 Hz, 1H), 4.11–4.05 (m, 1H), 3.97 (dd, *J* = 11.2, 3.0 Hz, 1H), 3.89 (s, 3H), 3.74 (dd, *J* = 11.0, 8.3 Hz, 1H), 2.12 (s, 3H); ^13^C NMR (125.7 MHz, acetone-d_6_ + CD_3_OD) *δ* 169.2, 162.6, 158.8, 154.1, 142.0, 141.9, 136.6, 136.3, 131.3, 129.6, 125.1, 124.6, 118.8, 117.5, 116.9, 115.1, 114.9, 100.7, 97.8, 95.9, 55.5, 54.2, 45.8, 41.3, 22.4; HRMS (FAB): *m*/*z* calcd for (C_25_H_21_ClN_2_NaO_4_Se) [M + Na]^+^ 551.0253, found 551.0246.

#### (*S*)-*N*-(2-(1-(Chloromethyl)-5-hydroxy-8-methoxy-2,3-dihydro-1*H*-benzo[*e*]indole-3-carbonyl)-6-methoxybenzo[*b*]selenophen-5-yl)acetamide (18o)

Pale yellow solid, 46%, ^1^H NMR (500.1 MHz, DMSO-d_6_) *δ* 10.39 (s, 1H), 9.28 (s, 1H), 8.59 (s, 1H), 8.23 (s, 1H), 8.01 (d, *J* = 9.2 Hz, 1H), 7.76 (s, 1H), 7.67 (brs, 1H), 7.09 (d, *J* = 2.3 Hz, 1H), 6.99 (dd, *J* = 9.2, 2.4 Hz, 1H), 4.75 (t, *J* = 9.9 Hz, 1H), 4.46 (d, *J* = 10.0 Hz, 1H), 4.17 (m, 1H), 4.03 (m, 1H), 3.92 (s, 3H), 3.89 (s, 3H), 3.42 (m, 1H), 2.14 (s, 3H); ^13^C NMR (125.7 MHz, DMSO-d_6_) *δ* 168.8, 162.0, 158.5, 154.2, 149.6, 142.7, 141.2, 138.1, 135.2, 131.3, 130.3, 126.4, 124.9, 119.2, 117.3, 115.4, 114.5, 107.0, 101.6, 98.3, 56.1, 55.5, 55.3, 47.5, 40.8, 23.9; HRMS (FAB): *m*/*z* calcd for (C_26_H_24_ClN_2_O_5_Se) [M + H]^+^ 559.0539, found 559.0533.

#### (*S*)-*N*-(2-(1-(Chloromethyl)-5-hydroxy-8-methoxy-2,3-dihydro-1*H*-benzo[*e*]indole-3-carbonyl)-6-(2-(dimethylamino)ethoxy)benzo[*b*]selenophen-5-yl)acetamide (18p)

Yellow solid, 28%, ^1^H NMR (500.1 MHz, DMSO-d_6_) *δ* 10.33 (s, 1H), 9.45 (s, 1H), 8.63 (s, 1H), 8.26 (s, 1H), 8.01 (d, *J* = 8.4 Hz, 1H), 7.84 (s, 1H), 7.68 (s, 1H), 7.12 (s, 1H), 6.99 (d, *J* = 9.2 Hz, 1H), 4.76 (t, *J* = 9.9 Hz, 1H), 4.46 (d, *J* = 11.0 Hz, 1H), 4.22–4.18 (m, 3H), 4.04 (m, 1H), 3.91 (s, 3H), 3.87 (m, 1H), 2.70–2.68 (m, 2H), 2.28 (s, 6H), 2.13 (s, 3H); HRMS (FAB): *m*/*z* calcd (C_29_H_31_ClN_3_O_5_Se) [M + H]^+^ 616.1117, found 616.1112.

#### (*S*)-*N*-(6-((2,5,8,11-Tetraoxatridecan-13-yl)oxy)-2-(1-(chloromethyl)-5-hydroxy-8-methoxy-2,3-dihydro-1*H*-benzo[*e*]indole-3-carbonyl)benzo[*b*]selenophen-5-yl)acetamide (18q)

Pale yellow solid, 25%, ^1^H NMR (500.1 MHz, CDCl_3_) *δ* 8.84 (s, 1H), 8.18 (d, *J* = 9.2 Hz, 1H), 8.07 (s, 1H), 7.97 (s, 1H), 7.32 (s, 1H), 7.01 (dd, *J* = 9.2, 2.1 Hz, 1H), 6.84 (s, 1H), 4.24–4.19 (m, 2H), 3.94–3.91 (m, 5H), 3.86–3.83 (m, 2H), 3.78–3.60 (m, 11H), 3.53–3.51 (m, 3H), 3.35 (m, 4H), 2.18 (s, 3H); HRMS (FAB): *m*/*z* calcd (C_34_H_40_ClN_2_O_9_Se) [M + H]^+^ 735.1588, found 735.1582.

#### (*S*)-*N*-(2-(1-(Chloromethyl)-5-hydroxy-8-methoxy-2,3-dihydro-1*H*-benzo[*e*]indole-3-carbonyl)benzo[*b*]selenophen-5-yl)butyramide (18r)

Yellow solid, 50%, ^1^H NMR (500.1 MHz, CDCl_3_) *δ* 8.18 (d, *J* = 9.0 Hz, 1H), 8.08 (s, 1H), 7.94 (s, 1H), 7.84 (s, 1H), 7.74 (s, 1H), 7.56 (d, *J* = 8.6 Hz, 1H), 7.33 (d, *J* = 8.2 Hz, 1H), 6.95 (dd, *J* = 9.2, 2.0 Hz, 1H), 6.57 (s, 1H), 4.21–4.14 (m, 2H), 3.83 (s, 3H), 3.64 (d, *J* = 9.7 Hz, 1H), 3.31–3.28 (m, 1H), 3.20 (t, *J* = 10.0 Hz, 1H), 2.35 (t, *J* = 7.4 Hz, 2H), 1.79–1.74 (m, 2H), 1.00 (t, *J* = 7.4 Hz, 3H); ^13^C NMR (125.7 MHz, CDCl_3_) *δ* 172.5, 164.4, 158.9, 155.0, 141.8, 141.7, 141.5, 138.1, 135.4, 131.2, 130.4, 125.7, 125.5, 120.3, 118.7, 118.2, 115.6, 115.1, 101.3, 99.4, 56.4, 55.5, 46.0, 42.0, 39.5, 19.3, 14.0; HRMS (FAB): *m*/*z* calcd (C_27_H_25_ClN_2_O_4_Se) [M + H]^+^ 557.0746, found 557.0741.

#### (*S*)-*N*-(2-(1-(Chloromethyl)-5-hydroxy-8-methoxy-2,3-dihydro-1*H*-benzo[*e*]indole-3-carbonyl)benzo[*b*]selenophen-5-yl)hexanamide (18s)

Yellow solid, 45%, ^1^H NMR (500.1 MHz, CDCl_3_) *δ* 10.03 (s, 1H), 8.18 (m, 1H), 8.08 (s, 2H), 7.90 (s, 1H), 7.75 (s, 1H), 7.57 (d, *J* = 7.9 Hz, 1H), 7.34 (d, *J* = 7.8 Hz, 1H), 6.96 (d, *J* = 9.3 Hz, 1H), 6.60 (s, 1H), 4.25–4.23 (m, 2H), 3.84 (s, 3H), 3.67 (d, *J* = 10.3 Hz, 1H), 3.41 (m, 1H), 3.29–3.24 (m, 1H), 2.37 (t, *J* = 6.7 Hz, 2H), 1.73 (m, 2H), 1.34 (m, 4H), 0.90 (m, 3H); ^13^C NMR (125.7 MHz, CDCl_3_) *δ* 172.7, 164.2, 158.9, 155.0, 141.8, 141.7, 141.6, 137.9, 135.6, 131.3, 130.4, 125.7, 125.4, 120.2, 118.6, 118.3, 115.6, 115.1, 101.2, 99.3, 55.6, 55.4, 46.0, 42.1, 37.6, 31.6, 25.5, 22.6, 14.1; HRMS (FAB): *m*/*z* calcd for (C_29_H_30_ClN_2_O_4_Se) [M + H]^+^ 585.1059, found 585.1054.

#### (*S*)-*N*-(2-(1-(Chloromethyl)-5-hydroxy-8-methoxy-2,3-dihydro-1*H*-benzo[*e*]indole-3-carbonyl)benzo[*b*]selenophen-5-yl)-3-(dimethylamino)propanamide (18t)

Yellow solid, 38%, ^1^H NMR (500.1 MHz, acetone-d_6_) *δ* 10.37 (s, 1H), 8.46 (s, 1H), 8.21 (d, *J* = 6.9 Hz, 1H), 8.15 (d, *J* = 9.2 Hz, 1H), 7.98 (d, *J* = 8.6 Hz, 1H), 7.75 (brs, 1H), 7.52 (d, *J* = 7.1 Hz, 1H), 7.21 (s, 1H), 7.03 (d, *J* = 9.2 Hz, 1H), 4.75 (t, *J* = 9.9 Hz, 1H), 4.66 (d, *J* = 11.0 Hz, 1H), 4.21 (m, 1H), 4.06 (dd, *J* = 11.0, 3.0 Hz, 1H), 3.96 (s, 3H), 3.83 (m, 1H), 2.80 (t, *J* = 5.9 Hz, 2H), 2.60 (t, *J* = 5.8 Hz, 2H), 2.41 (s, 6H); HRMS (FAB): *m*/*z* calcd for (C_28_H_29_ClN_3_O_4_Se) [M + H]^+^ 586.1012, found 586.1006.

#### (*S*)-*N*-(2-(1-(Chloromethyl)-5-hydroxy-8-methoxy-2,3-dihydro-1*H*-benzo[*e*]indole-3-carbonyl)benzo[*b*]selenophen-5-yl)-3-(methylthio)propanamide (18u)

Brown solid, 54%, ^1^H NMR (500.1 MHz, acetone-d_6_) *δ* 9.41 (s, 1H), 9.27 (s, 1H), 8.51 (s, 1H), 8.20 (s, 1H), 8.15 (d, *J* = 9.2 Hz, 1H), 7.98 (d, *J* = 8.7 Hz, 1H), 7.75 (brs, 1H), 7.54 (d, *J* = 8.6 Hz, 1H), 7.20 (d, *J* = 2.0 Hz, 1H), 7.03 (dd, *J* = 9.2, 2.2 Hz, 1H), 4.75 (t, *J* = 9.7 Hz, 1H), 4.65 (d, *J* = 11.0 Hz, 1H), 4.20–4.15 (m, 1H), 4.05 (dd, *J* = 11.2, 3.2 Hz, 1H), 3.95 (s, 3H), 3.82 (dd, *J* = 11.1, 8.5 Hz, 1H), 2.85 (t, *J* = 7.1 Hz, 2H), 2.72 (t, *J* = 7.2 Hz, 2H), 2.11 (s, 3H); ^13^C NMR (500.1 MHz, CDCl_3_) *δ* 170.5, 164.3, 158.9, 154.9, 141.8, 141.7, 141.6, 138.2, 135.3, 131.3, 130.2, 125.7, 125.6, 120.1, 118.6, 118.2, 115.7, 115.2, 101.2, 99.4, 56.3, 55.5, 46.0, 42.0, 37.2, 30.0, 29.8; HRMS (FAB): *m*/*z* calcd for (C_27_H_25_ClN_2_NaO_4_SSe) [M + Na]^+^ 611.0286, found 611.0280.

#### (*S*)-4-Acetamido-*N*-(2-(1-(chloromethyl)-5-hydroxy-8-methoxy-2,3-dihydro-1*H*-benzo[*e*]indole-3-carbonyl)benzo[*b*]selenophen-5-yl)-1-methyl-1*H*-pyrrole-2-carboxamide (18v)

Yellow solid, 48%, ^1^H NMR (500.1 MHz, CD_3_OD + CDCl_3_) *δ* 8.24 (s, 1H), 8.07 (d, *J* = 9.0 Hz, 1H), 7.87 (s, 1H), 7.77 (d, *J* = 8.5 Hz, 1H), 7.42 (d, *J* = 8.5 Hz, 1H), 7.38 (s, 1H), 7.02 (s, 1H), 6.94 (d, *J* = 9.1 Hz, 1H), 6.82 (s, 2H), 4.51–4.45 (m, 2H), 3.88 (m, 2H), 3.84 (s, 3H), 3.83 (s, 3H), 3.42 (m, 1H), 2.02 (s, 3H); ^13^C NMR (500.1 MHz, CD_3_OD + CDCl_3_) *δ* 169.4, 164.0, 161.1, 159.5, 155.2, 142.5, 142.1, 137.9, 136.5, 131.8, 130.6, 125.9 (2C), 123.5, 122.1, 120.8, 120.2 (2C), 118.9, 118.6, 116.0, 115.6, 105.6, 101.6, 98.6, 56.5, 55.6, 46.0, 42.8, 36.8, 22.9; HRMS (FAB): *m*/*z* calcd for (C_31_H_28_ClN_4_O_5_Se) [M + H]^+^ 651.0913, found 651.0908.

#### (*S*)-*N*-(2-(1-(Chloromethyl)-5-hydroxy-8-methoxy-2,3-dihydro-1*H*-benzo[*e*]indole-3-carbonyl)benzo[*b*]selenophen-5-yl)methanesulfonamide (18w)

Brown solid, 44%, ^1^H NMR (500.1 MHz, acetone-d_6_) *δ* 9.24 (s, 1H), 8.70 (s, 1H), 8.26 (s, 1H), 8.15 (d, *J* = 9.2 Hz, 1H), 8.08 (d, *J* = 8.6 Hz, 1H), 7.99 (d, *J* = 2.1 Hz, 1H), 7.76 (brs, 1H), 7.42 (dd, *J* = 8.6, 2.3 Hz, 1H), 7.21 (d, *J* = 2.4 Hz, 1H), 4.77 (t, *J* = 9.7 Hz, 1H), 4.65 (dd, *J* = 10.9, 1.7 Hz, 1H), 4.22–4.64 (m, 1H), 4.06 (dd, *J* = 11.2, 3.3 Hz, 1H), 3.95 (s, 3H), 3.83 (dd, *J* = 11.2, 8.4 Hz, 1H), 3.05 (s, 3H); ^13^C NMR (500.1 MHz, acetone-d_6_) *δ* 160.1, 155.3, 144.0, 143.8, 143.5, 137.1, 132.9, 130.4, 127.4, 126.1, 121.4 (2C), 119.6, 119.3, 118.7, 116.5 (2C), 102.1, 99.4, 56.6, 55.7, 47.5, 42.8, 39.2; HRMS (FAB): *m*/*z* calcd for (C_24_H_21_ClN_2_O_5_SSe) [M]^+^ 564.000, found 600.9741 [M + K]^+^.

#### (*S*)-*N*-(2-(1-(Chloromethyl)-5-hydroxy-8-methoxy-2,3-dihydro-1*H*-benzo[*e*]indole-3-carbonyl)-6-methoxybenzo[*b*]selenophen-5-yl)methanesulfonamide (18x)

Pale yellow solid, 26%, ^1^H NMR (500.1 MHz, DMSO-d_6_) *δ* 10.31 (s, 1H), 9.01 (brs, 1H), 8.27 (s, 1H), 8.02 (d, *J* = 9.0 Hz, 1H), 7.89 (s, 1H), 7.84 (s, 1H), 7.68 (s, 1H), 7.12 (s, 1H), 7.00 (d, *J* = 7.8 Hz, 1H), 4.75 (t, *J* = 9.2 Hz, 1H), 4.47 (d, *J* = 10.8 Hz, 1H), 4.20 (m,1H), 4.03 (d, 10.8 Hz, 1H), 3.93 (s, 3H), 3.91 (s, 3H), 3.87 (m, 1H), 2.99 (s, 3H).

Spectral characterization of new compounds (19a–d) is described in our previous work.^21^

### Cell growth inhibition assay

Her-2 positive human gastric cancer cell NCI-N87 and human ovarian cancer cell SK-OV3 (Both cell lines were purchased from American Type Culture Collection (ATCC; Manassas, VA, USA)) were seeded 384-well plates at 500 cells per well. After 2 h plating, cells were treated with toxins in 5-fold and 14-point serial dilution series in quadruplicate. After 3 days of incubation at 37 °C in a 5% CO_2_ humidified incubator, cell viability was checked using an adenosine triphosphate monitoring system based on firefly luciferase (ATPliteTM 1step, PerkinElmer, MA, USA). IC_50_ values were calculated as an average of quadruplicated experiments (GraphPad Prism 5.0, CA, and USA).

## Conclusions

In summary, a series of benzoselenophene and heteroaromatic analogues of *seco*-MCBI (18a–w) were synthesized, and their cytotoxicities against the human gastric NCI-N87 and human ovarian SK-OV3 cancer cell lines were evaluated. The incorporation of a methoxy group at the C-7 position in *seco*-CBI enhances the cytotoxicity through additional van der Waals interactions, and it was found to be much more potent than a *seco*-CBI-based analogue. The *seco*-MCBI-benzoselenophene analogues (18h–x) exhibited substitution effects on the biological activity and allowed for detailed study of the structure–activity relationship (SAR). Among the series of *N*-substituted analogues (18h–x), the 18n, 18r and 18u–v analogues were more potent than *seco*-MCBI-TMI and other compounds. The higher potency of 18r than 18n results from the extended length of the C-5 substituents of the benzoselenophene unit, which has greater hydrophobicity and van der Waals interactions in the DNA minor groove. The potency of *N*-butyramido analogue 18r was diminished after substitution with the hydrophilic *N*,*N*-dimethyl amino group, but it maintained or slightly increased the activity after substitution with *S*-methyl group. The activity was reduced in hydrophilic analogues 18p (IC_50_ = 190, 37 pM against NCI-N87 and SK-OV3, respectively) and 18q (IC_50_ = 1000, 260 pM against NCI-N87 and SK-OV3, respectively). However, the activities of hydrophilic analogues 18p and 18t are sufficiently high for use as a cytotoxic agent in ADCs. Overall, we successfully synthesized and screened potent candidates of benzoselenophene analogues of duocarmycin that can be used to develop effective therapeutics for advanced chemotherapy.

## Conflicts of interest

There are no conflicts to declare.

## Supplementary Material

RA-009-C9RA04749B-s001
