# Supplementary material for: Synthesis and biological evaluation of potent benzoselenophene and heteroaromatic analogues of (S)-1-(chloromethyl)-8-methoxy-2,3-dihydro-1H-benzo[e]indol-5-ol (seco-MCBI)
Source: RSC Adv. 2019 Sep 16;9(50):29023–36. doi: 10.1039/c9ra04749b (PMC9071829; doi:10.1039/c9ra04749b)

Electronic Supplementary Material (ESI) for RSC Advances.
This journal is © The Royal Society of Chemistry 2019

Supplementary Information

Synthesis and biological evaluation of novel potent benzoselenophene and heteroaromatic analogs of 1,2,9,9a-tetrahydro-7-methoxycyclopropa[c]benzo[e]indol-4-one (MCBI)

Amol B. Mhetre,[a] Eppakayala Sreedhar,[a] Rashmi Dubey,[a] Ganesh Sable,[a] Hangeun Lee,[a] Heekyoung Yang,[b] Kyoungmin Lee,[b] Do-Hyun Nam,[b] and Dongyeol Lim*[a]

Table of content

^{1}H NMR and ^{13}C NMR spectra of ester intermediate of compound **3**……S1

^{1}H NMR and ^{13}C NMR spectra of compound **3**……S2

^{1}H NMR and ^{13}C NMR spectra of ester intermediate of compound **4**……S3

^{1}H NMR and ^{13}C NMR spectra of compound **4**……S4

^{1}H NMR and ^{13}C NMR spectra of ester intermediate of compound **5**……S5

^{1}H NMR and ^{13}C NMR spectra of compound **5**……S6

^{1}H NMR and ^{13}C NMR spectra of ester intermediate of compound **6**……S7

^{1}H NMR and ^{13}C NMR spectra of compound **6**……S8

^{1}H NMR and ^{13}C NMR spectra of (*N*-Boc) ester intermediate of compound **7**……S9

^{1}H NMR and ^{13}C NMR spectra of (*N*-Ac) ester intermediate of compound **7**……S10

^{1}H NMR and ^{13}C NMR spectra of compound **7**……S11

^{1}H NMR and ^{13}C NMR spectra of compound **9**……S12

^{1}H NMR and ^{13}C NMR spectra of compound **10**……S13

^{1}H NMR and ^{13}C NMR spectra of compound **11**……S14

^{1}H NMR spectra of ester intermediate of compound **13**……S15

^{1}H NMR and ^{13}C NMR spectra of compound **13**……S16

^{1}H NMR and ^{13}C NMR spectra of ester intermediate of compound **14**……S17

^{13}C NMR spectra of compound **14**……………………………………………………………………………………………………...S18

^{1}H NMR and ^{13}C NMR spectra of compounds **18a-x**…………………………………………………………………..S18–S14

Ester Intermediate of compound **3**

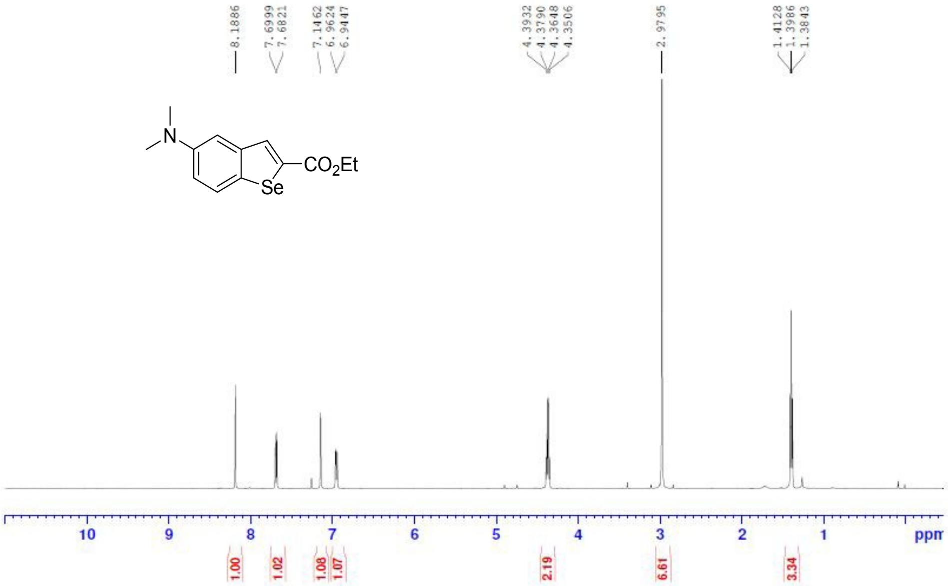

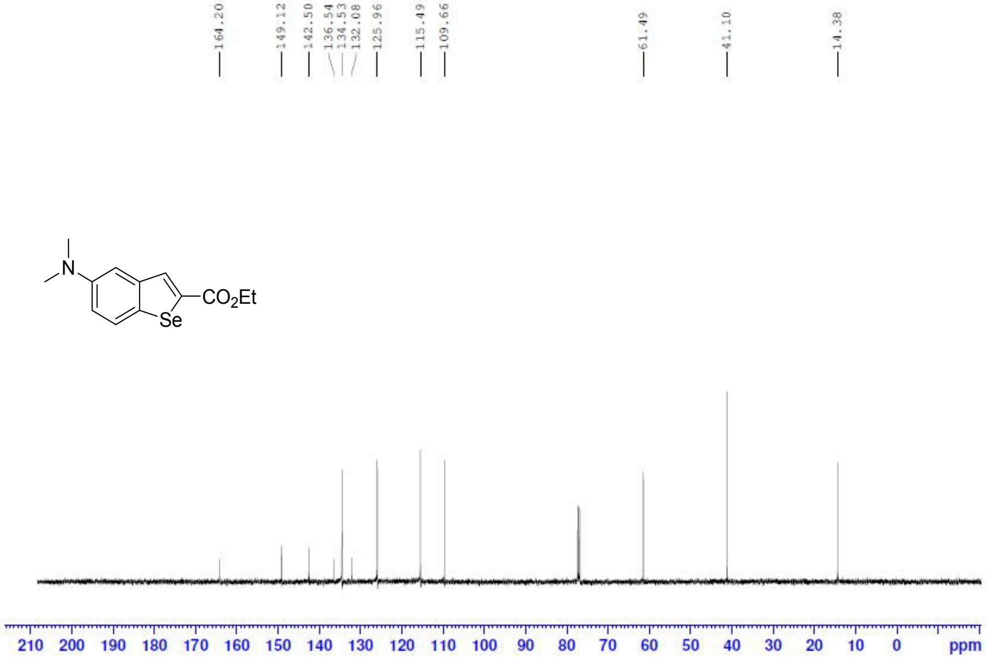

Compound **3**

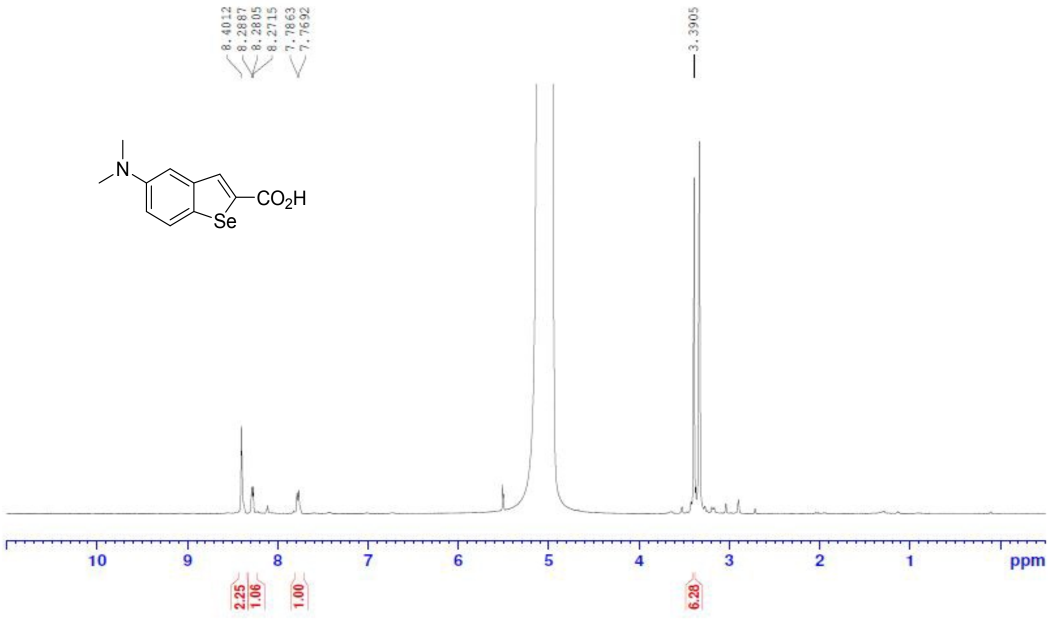

8.4012
8.2887
8.2805
8.2715
7.7863
7.7692
3.3905
CO2H
Se
N
10 9 8 7 6 5 4 3 2 1 ppm
2.25
1.06
1.00
6.28

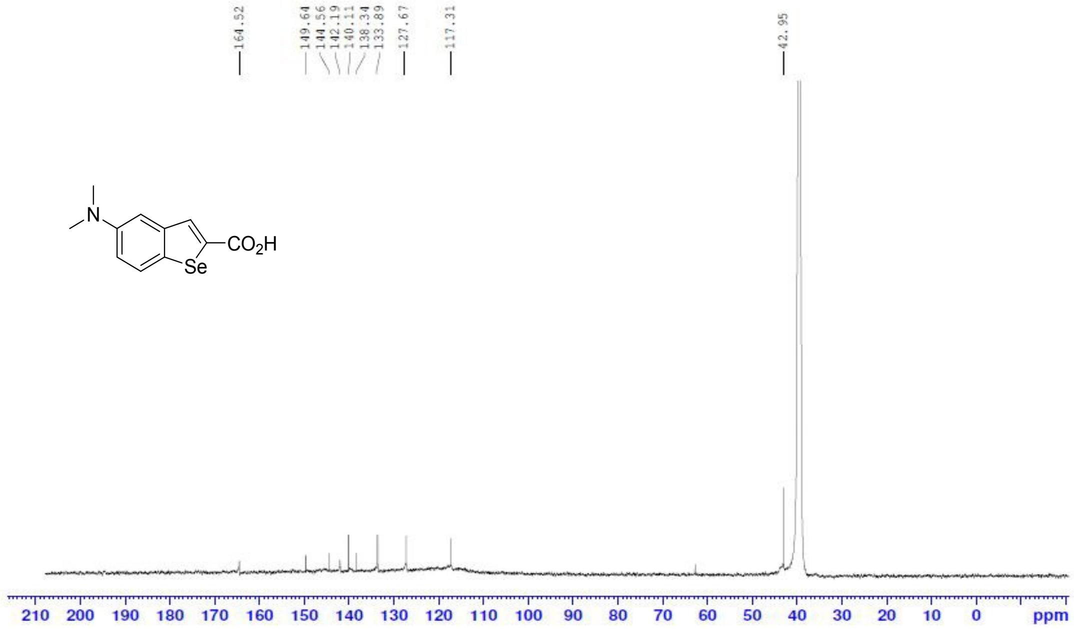

164.52
149.64
144.56
142.19
140.11
138.34
133.89
127.67
117.31
42.95
CO2H
Se
N
210 200 190 180 170 160 150 140 130 120 110 100 90 80 70 60 50 40 30 20 10 0 ppm

Ester Intermediate of compound **4**

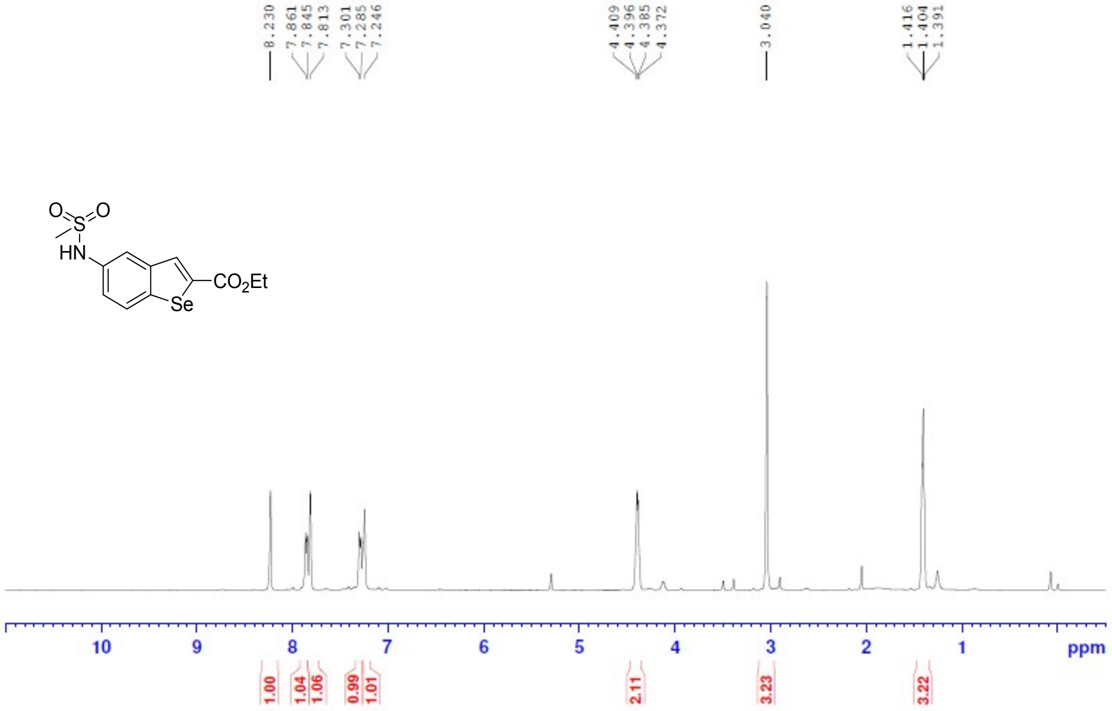

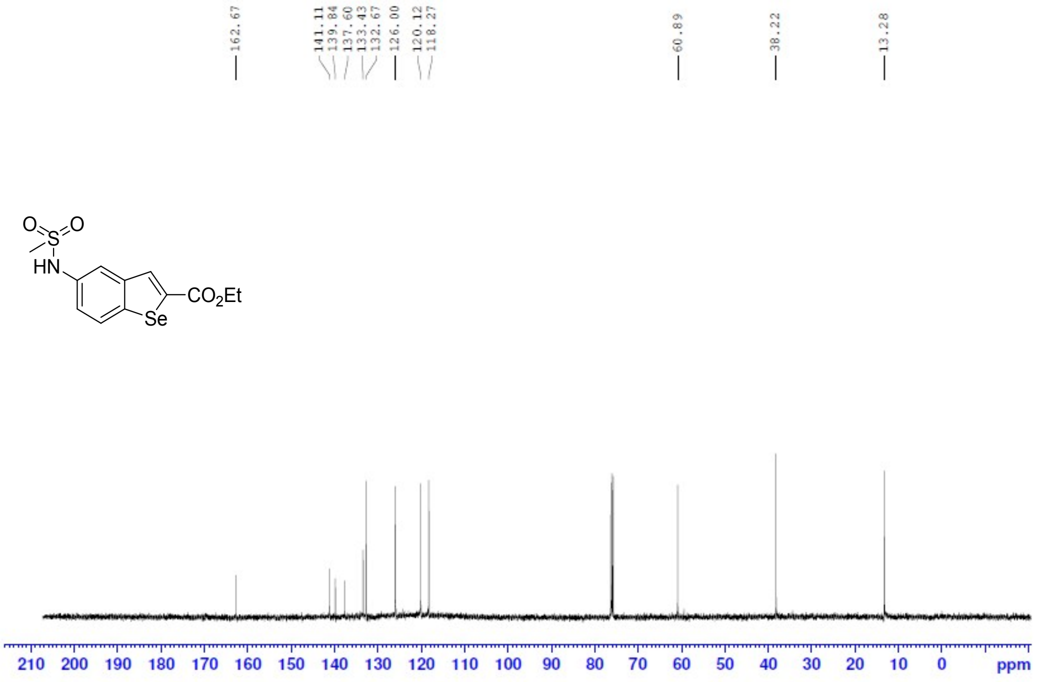

Compound **4**

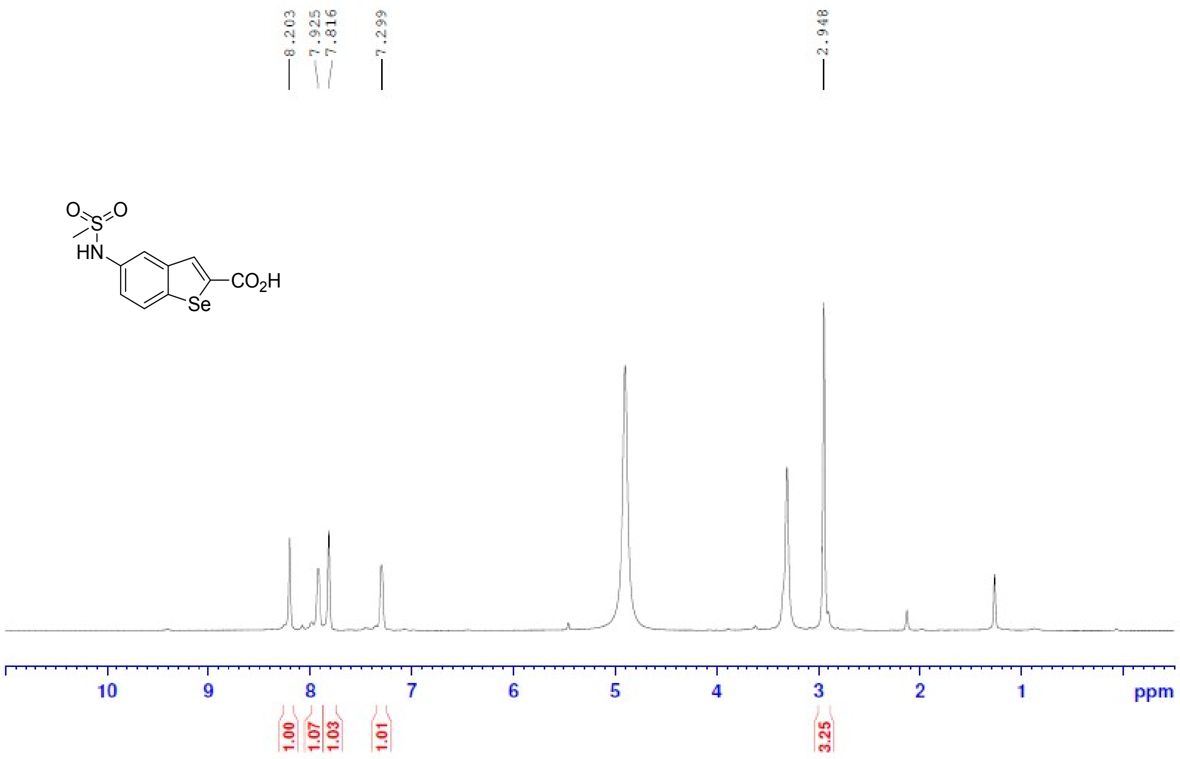

8.203
7.925
7.816
7.299
2.948
CO2H
HN
Se
10
9
8
7
6
5
4
3
2
1
ppm
1.00
1.07
1.03
1.01
3.25

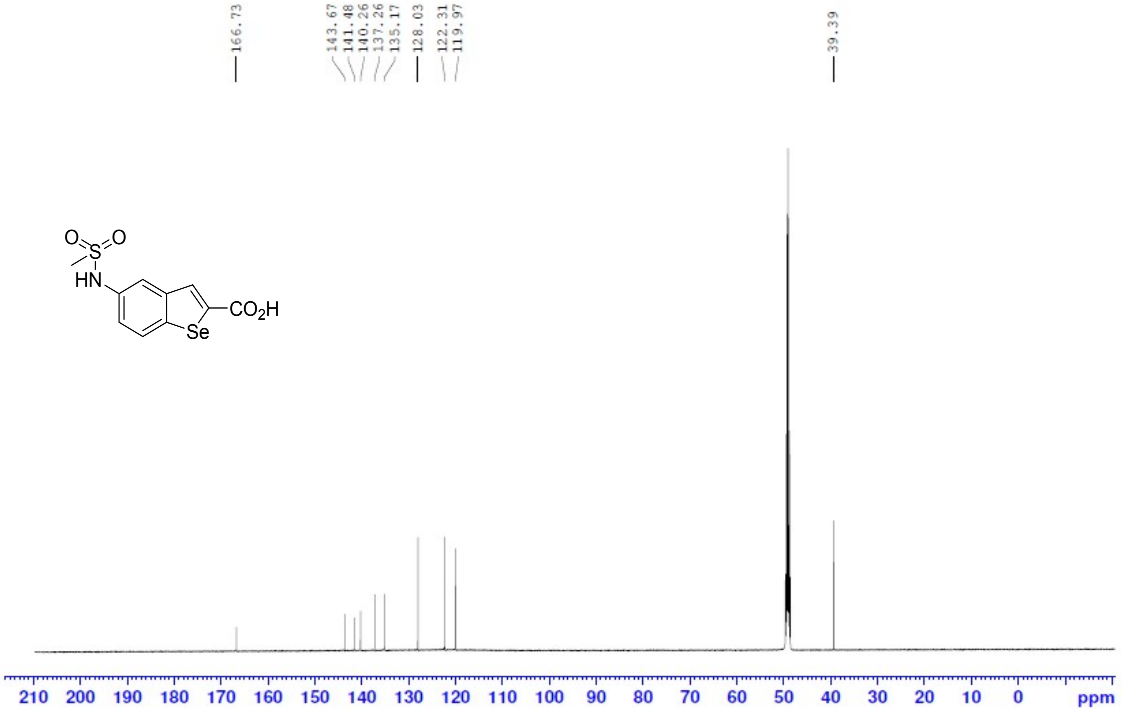

166.73
143.67
141.48
140.26
137.26
135.17
128.03
122.31
119.97
39.39
CO2H
HN
Se
210
200
190
180
170
160
150
140
130
120
110
100
90
80
70
60
50
40
30
20
10
0
ppm

Ester Intermediate of compound **5**

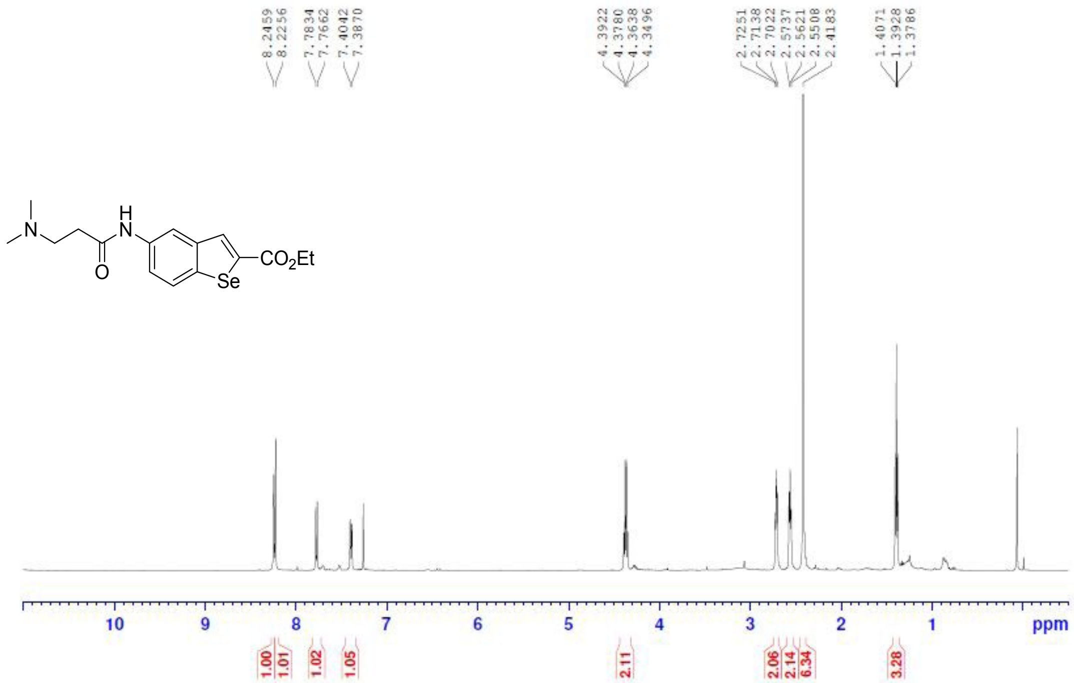

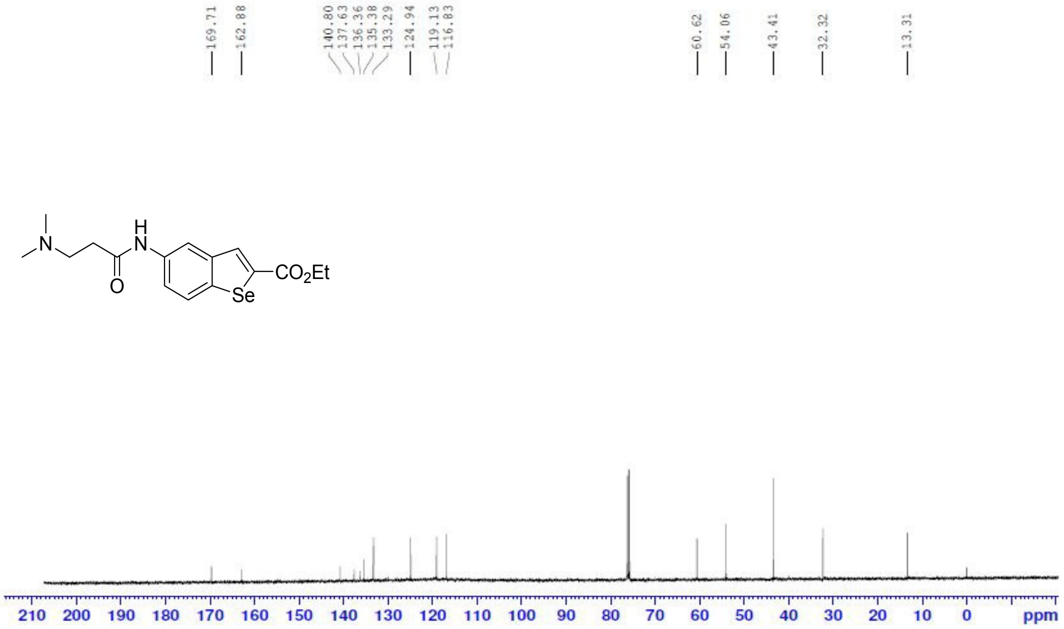

Compound **5**

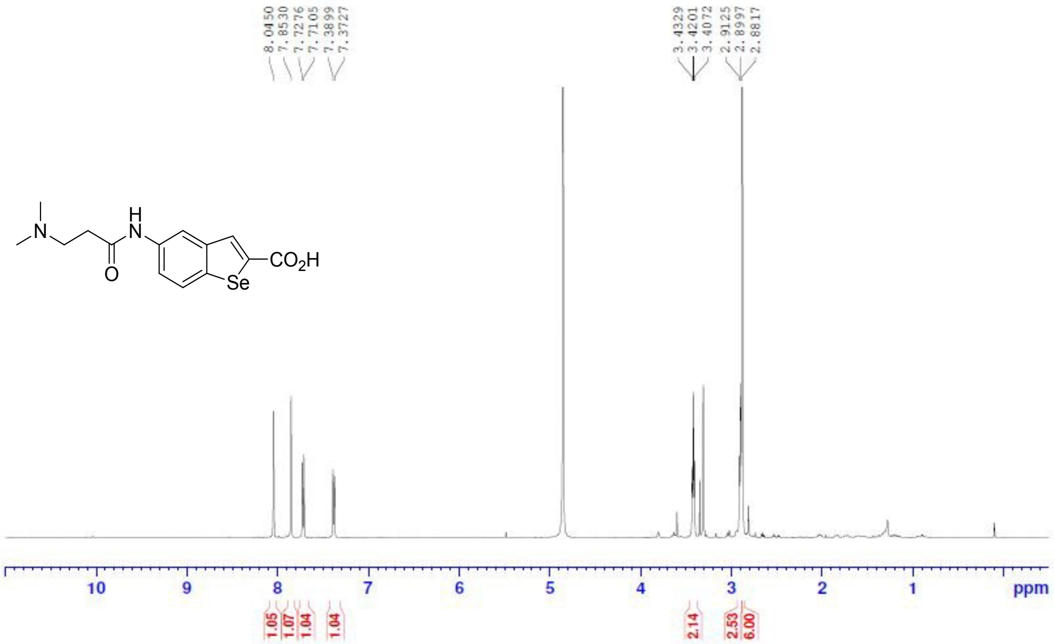
8.0450
7.8530
7.7276
7.7105
7.3899
7.3727
3.4329
3.4201
3.4072
2.9125
2.8997
2.8817
N
H
N
O
CO2H
Se
10
9
8
7
6
5
4
3
2
1
ppm
1.05
1.07
1.04
1.04
2.14
2.53
6.00

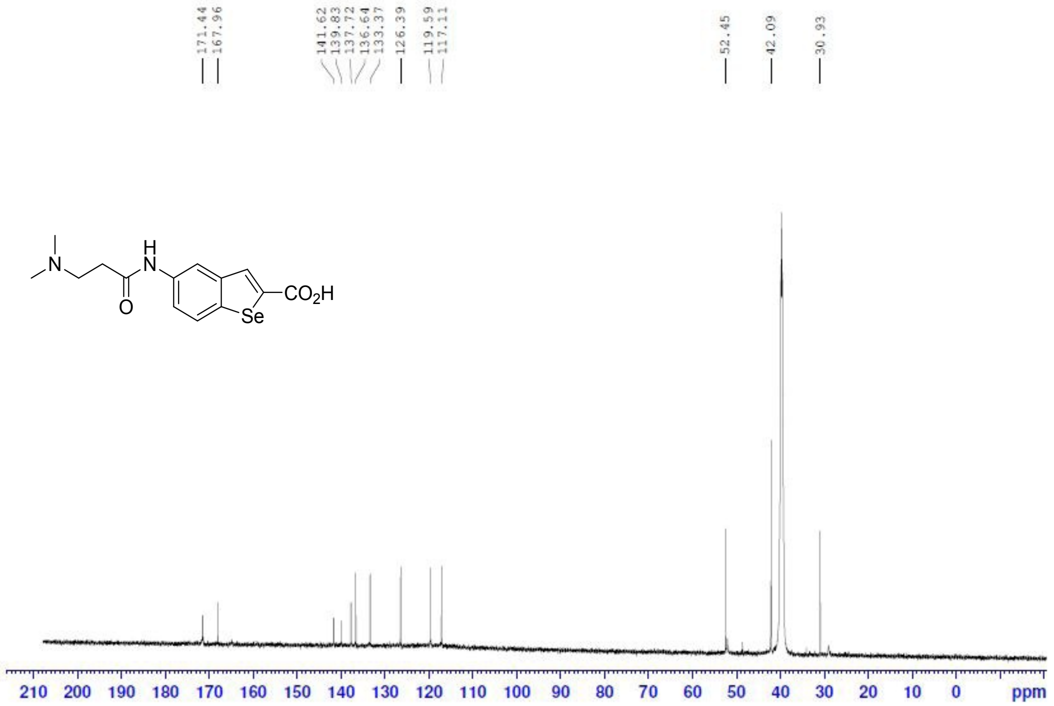
171.44
167.96
141.62
139.83
137.72
136.64
133.37
126.39
119.59
117.11
52.45
42.09
30.93
N
H
N
O
CO2H
Se
210
200
190
180
170
160
150
140
130
120
110
100
90
80
70
60
50
40
30
20
10
0
ppm

Ester intermediate of compound **6**

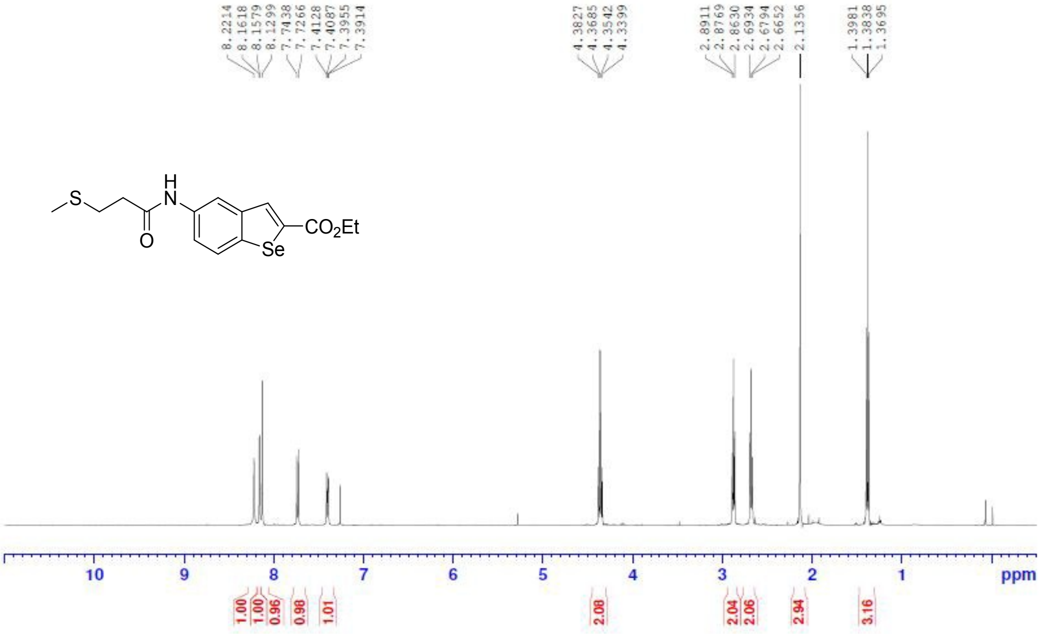

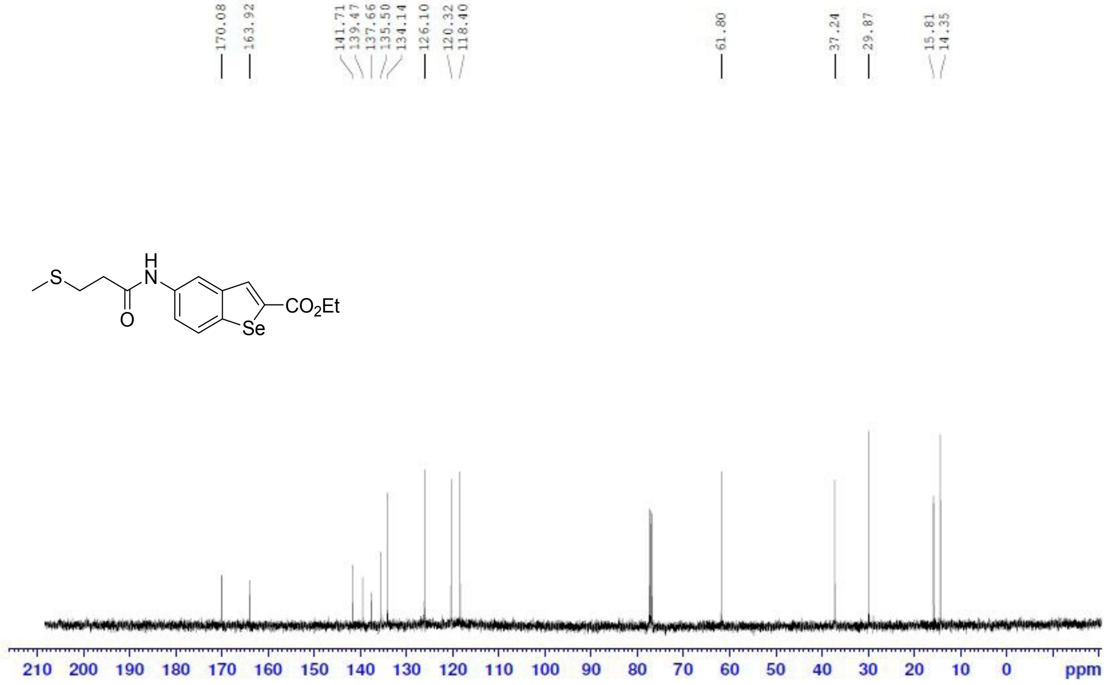

Compound **6**

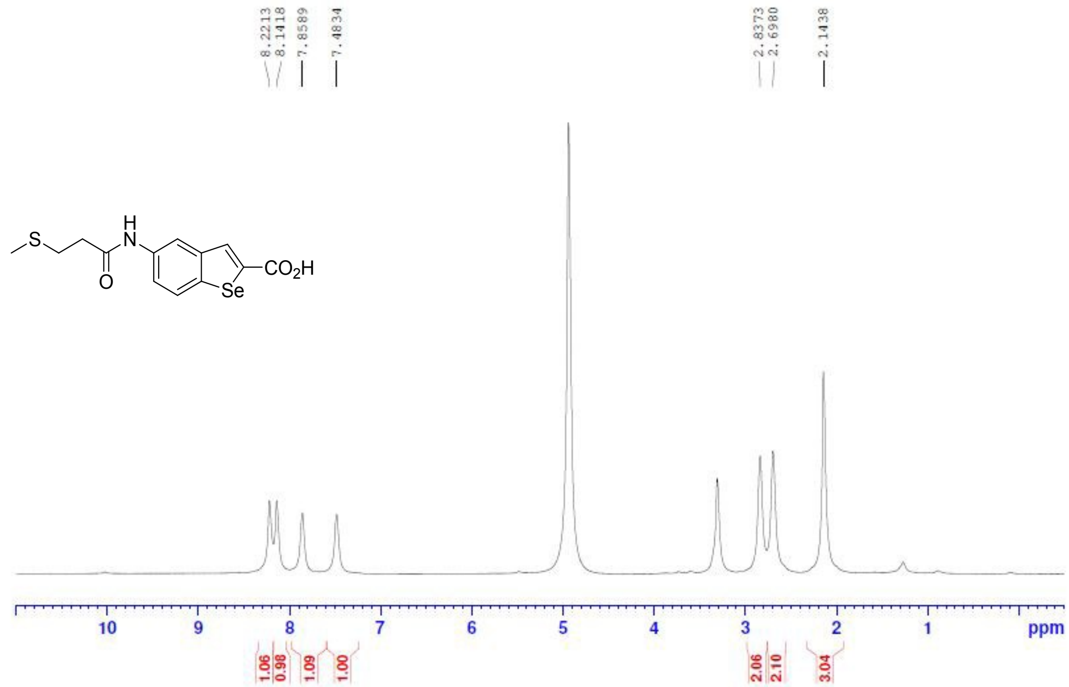

8.2213
8.1418
7.8589
7.4834
2.8373
2.6980
2.1438
S
H
N
O
CO2H
Se
10 9 8 7 6 5 4 3 2 1 ppm
1.06
0.98
1.09
1.00
2.06
2.10
3.04

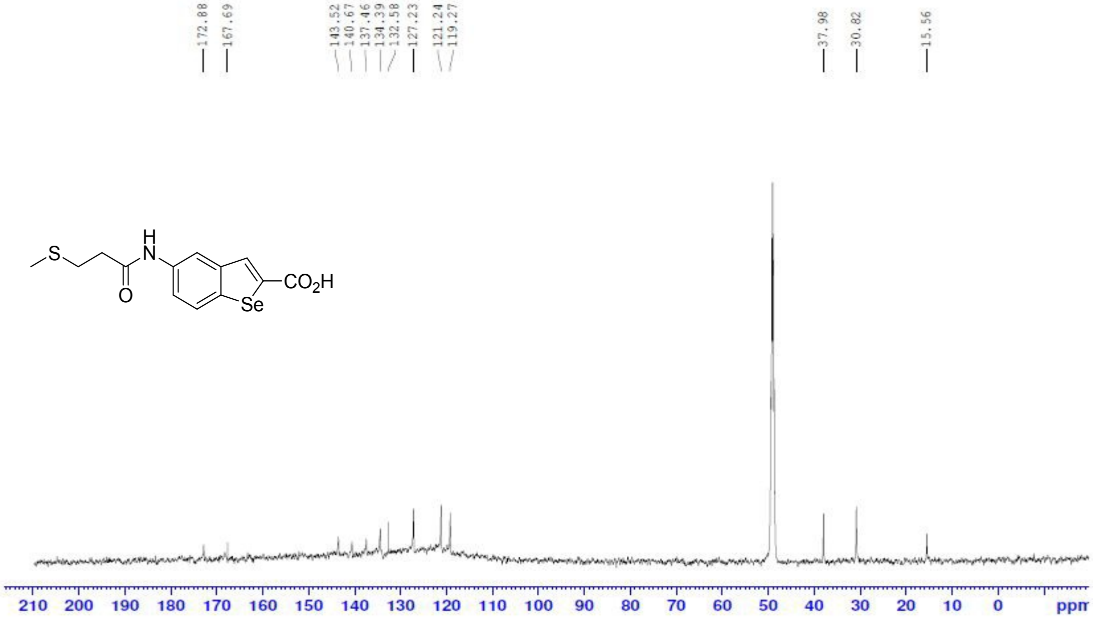

172.88
167.69
143.52
140.67
137.46
134.39
132.58
127.23
121.24
119.27
37.98
30.82
15.56
S
H
N
O
CO2H
Se
210 200 190 180 170 160 150 140 130 120 110 100 90 80 70 60 50 40 30 20 10 0 ppm

(*N*-Boc) ester intermediate of compound **7**

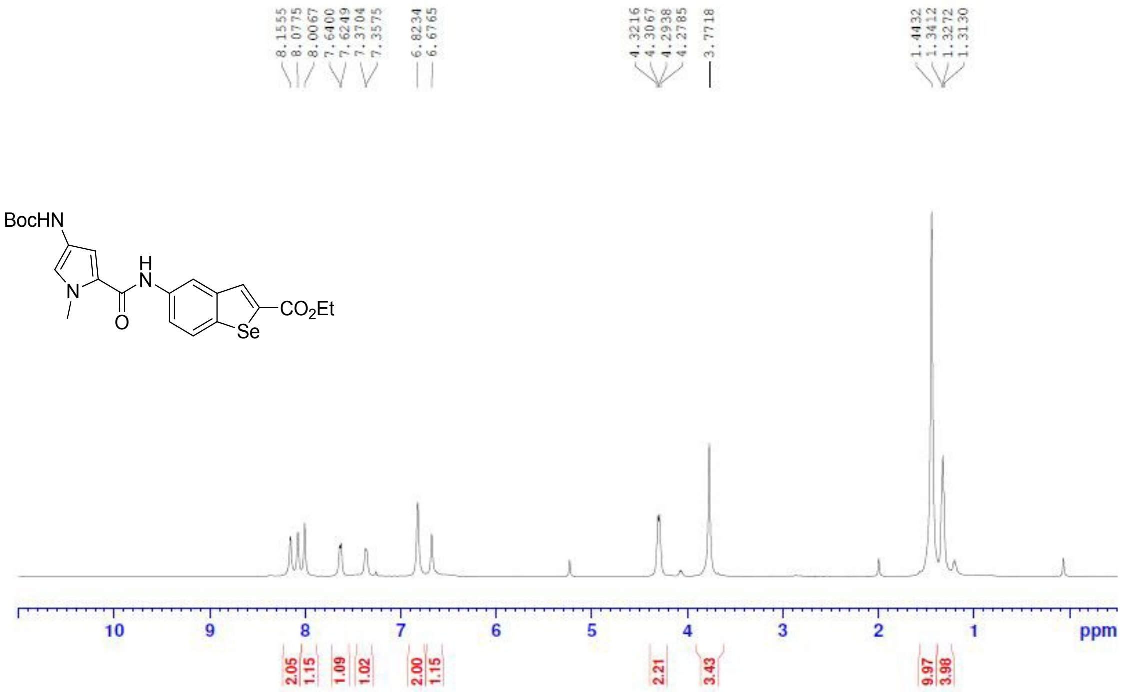

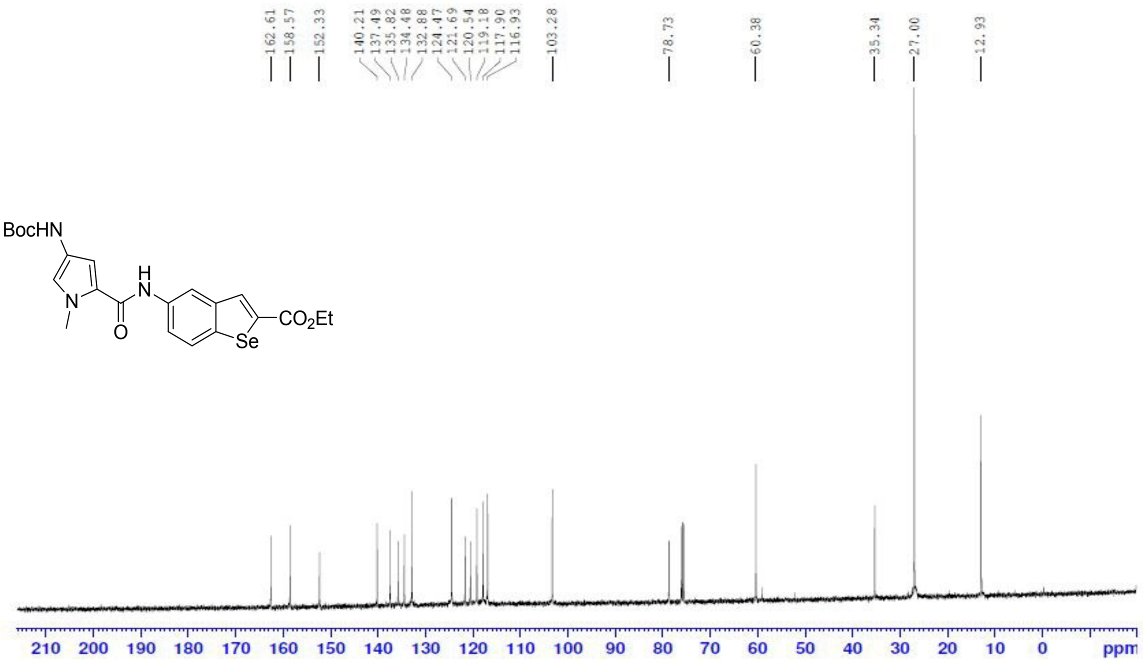

(*N*-Acetyl) ester intermediate of compound **7**

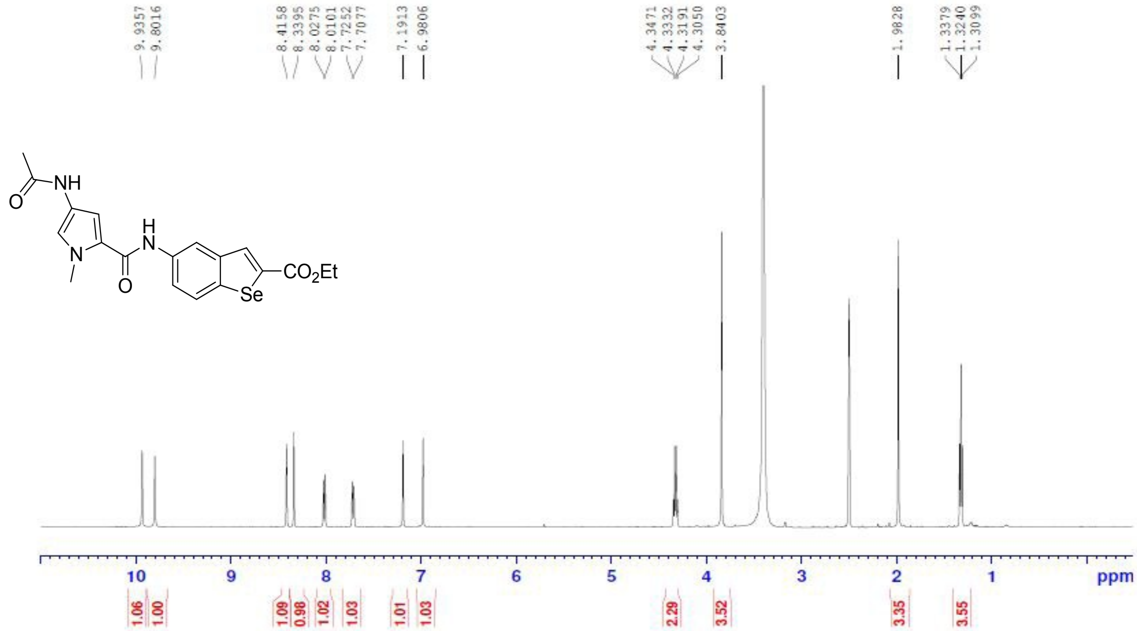

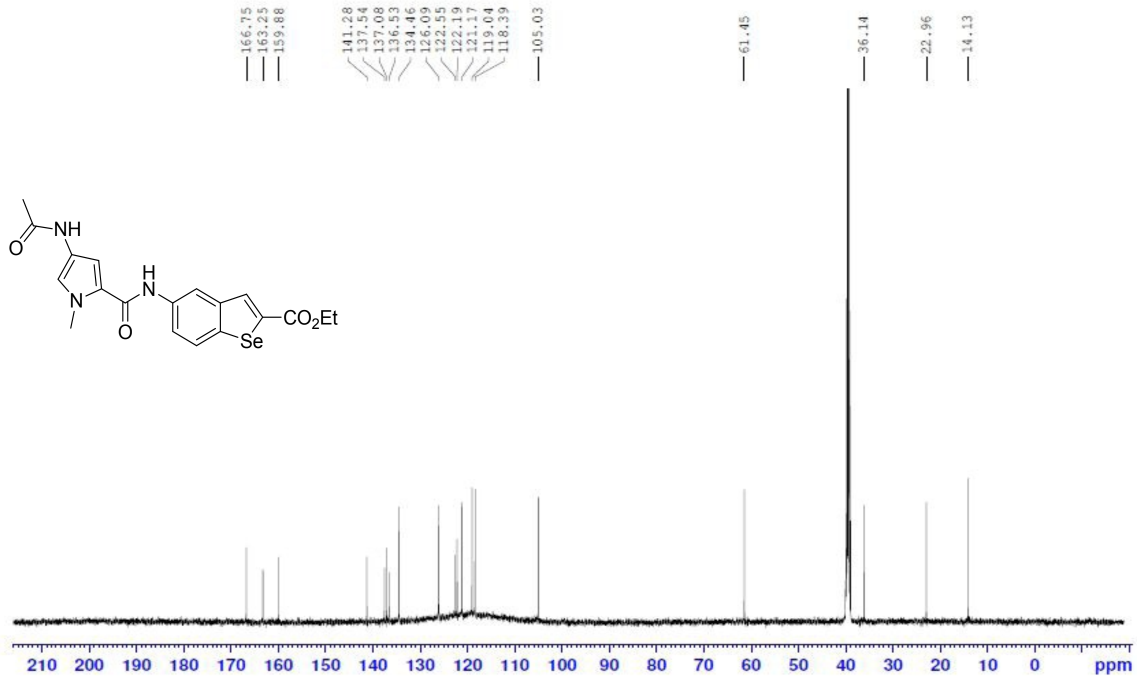

Compound **7**

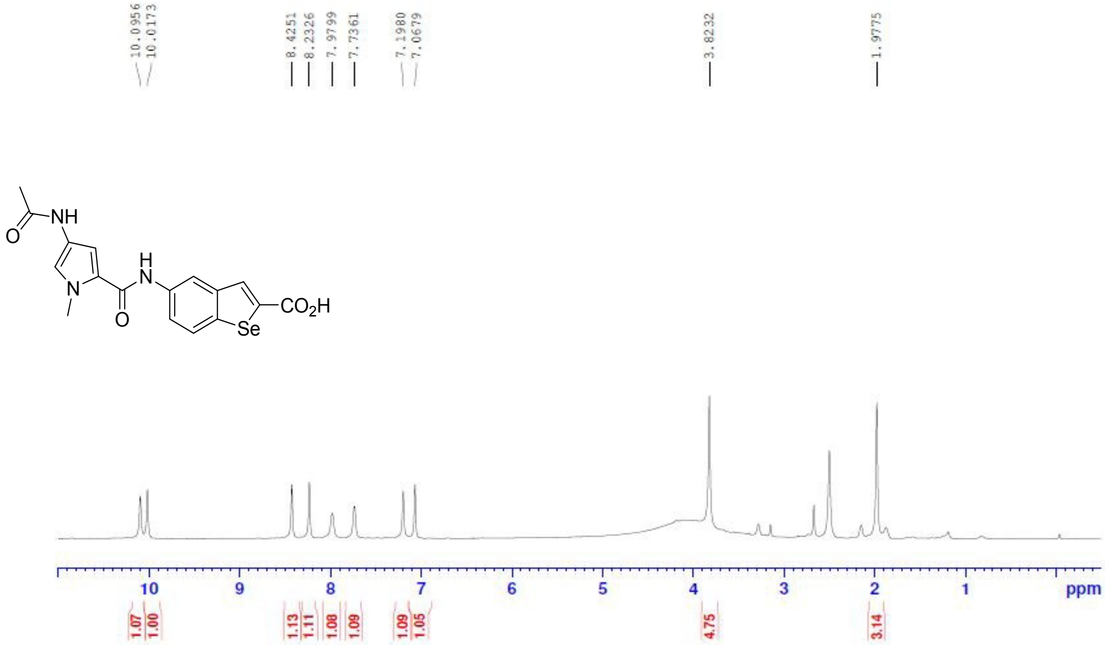

10.0956
10.0173
8.4251
8.2326
7.9799
7.7361
7.1980
7.0679
3.8232
1.9775
NH
O
H
N
N
CO2H
Se
O
10
9
8
7
6
5
4
3
2
1
ppm
1.07
1.00
1.13
1.11
1.08
1.09
1.09
1.05
4.75
3.14

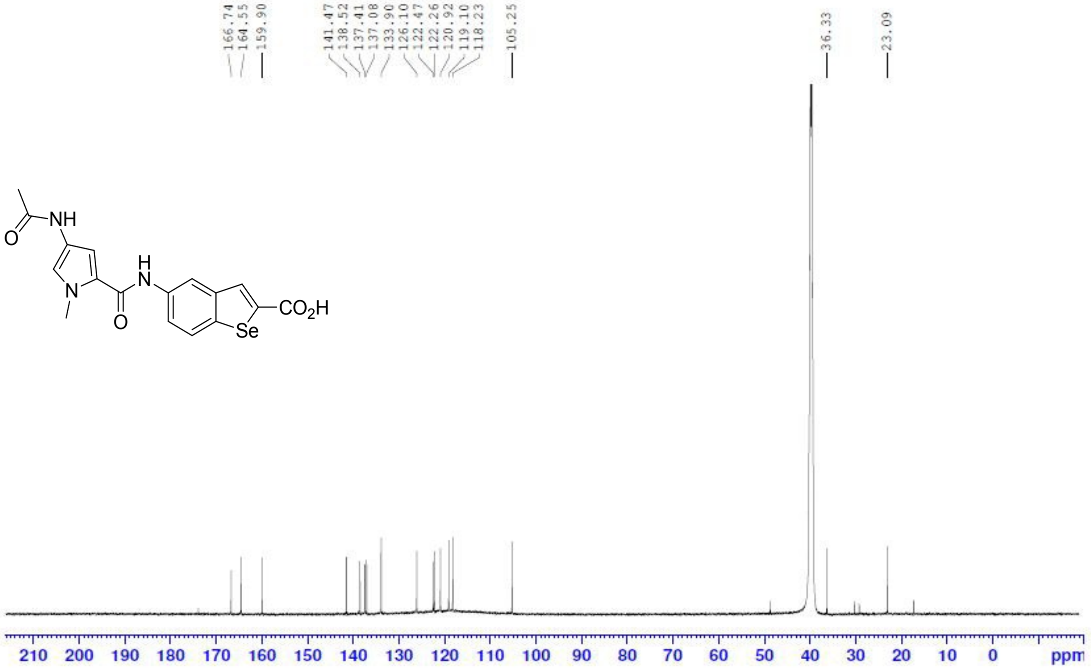

166.74
164.55
159.90
141.47
138.52
137.41
137.08
133.90
126.10
122.47
122.26
120.92
119.10
118.23
105.25
36.33
23.09
NH
O
H
N
N
CO2H
Se
O
210
200
190
180
170
160
150
140
130
120
110
100
90
80
70
60
50
40
30
20
10
0
ppm

Compound **9**

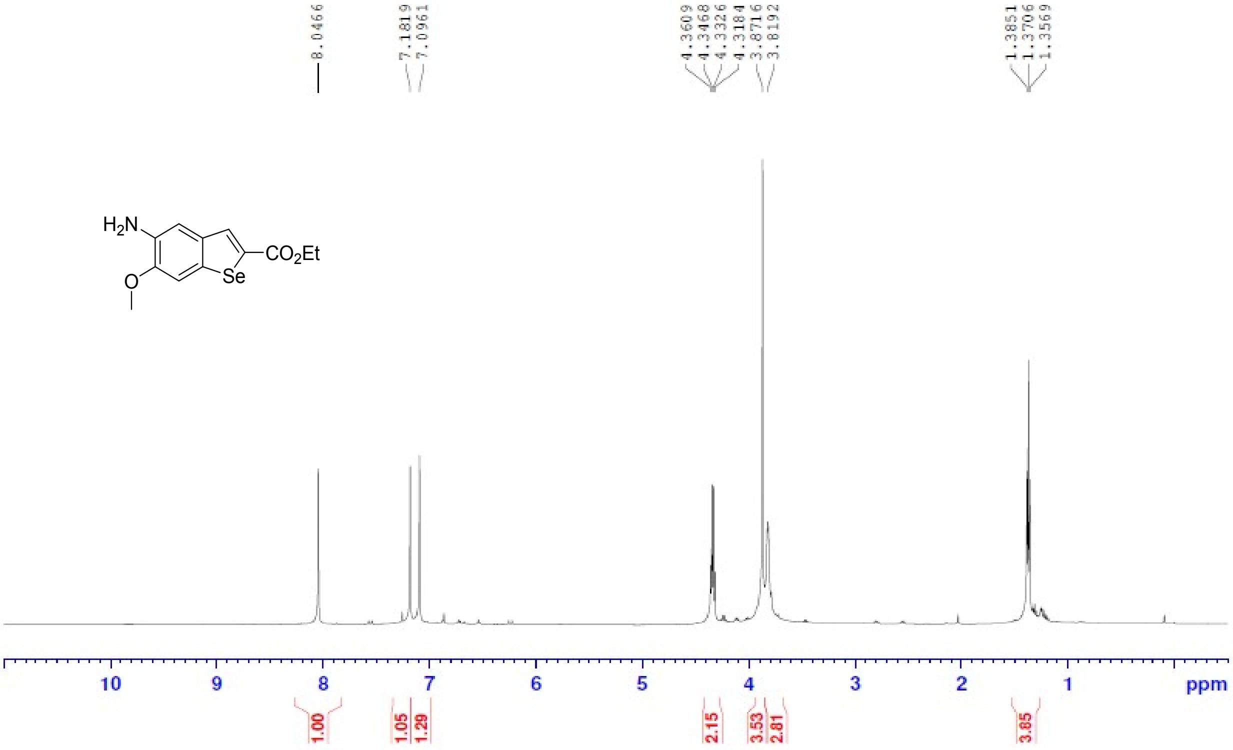
8.0466
7.1819
7.0961
4.3609
4.3468
4.3326
4.3184
3.8716
3.8192
1.3851
1.3706
1.3569
H2N
CO2Et
Se
O
10
9
8
7
6
5
4
3
2
1
ppm
1.00
1.05
1.29
2.15
3.53
2.81
3.85

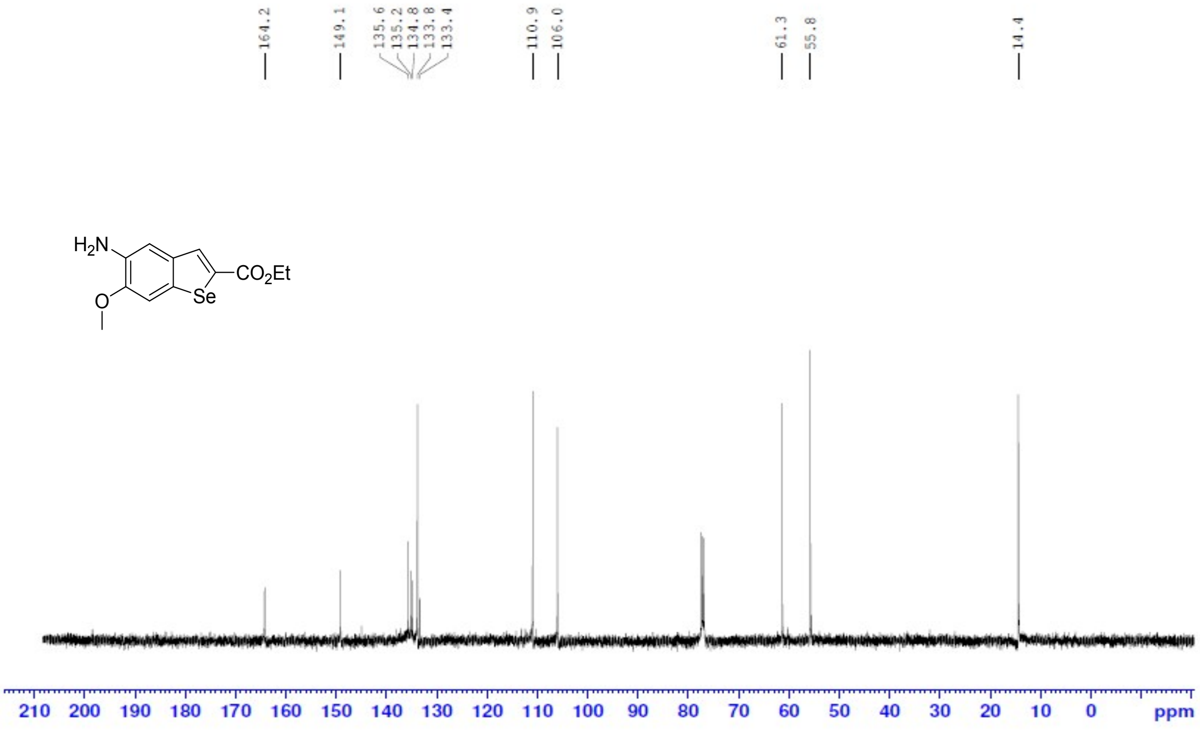
164.2
149.1
135.6
135.2
134.8
133.8
133.4
110.9
106.0
61.3
55.8
14.4
H2N
CO2Et
Se
O
210
200
190
180
170
160
150
140
130
120
110
100
90
80
70
60
50
40
30
20
10
0
ppm

Compound **10**

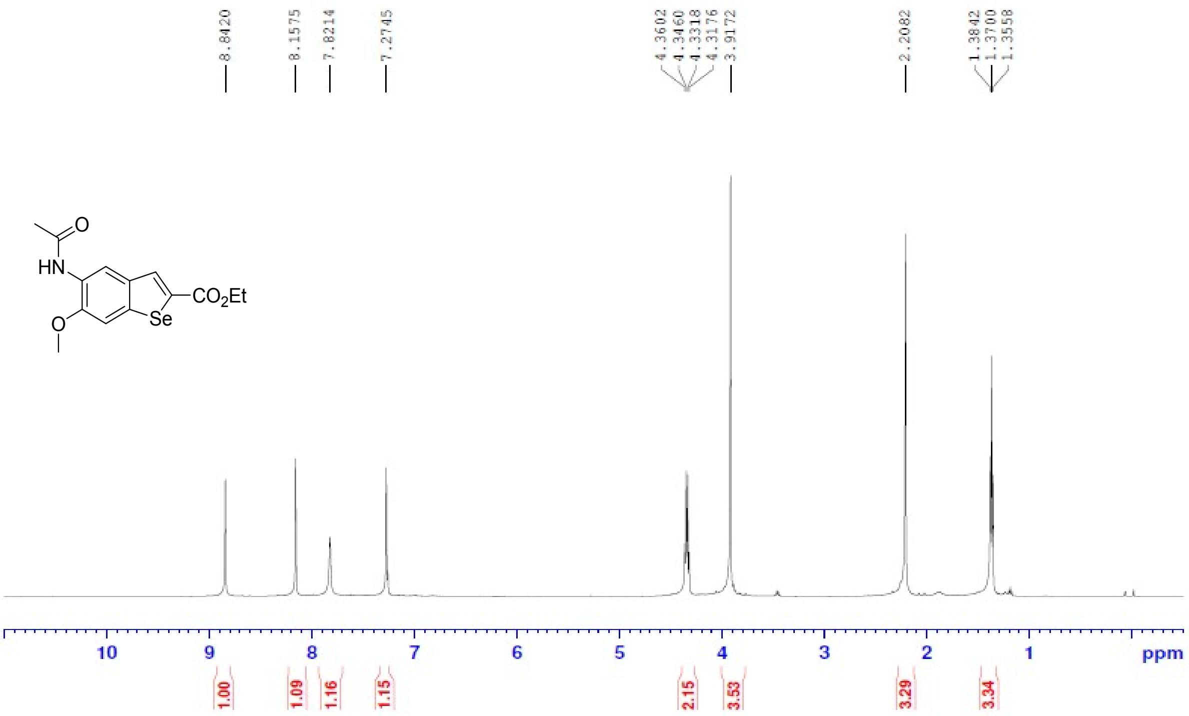

8.8420
8.1575
7.8214
7.2745
4.3602
4.3460
4.3318
4.3176
3.9172
2.2082
1.3842
1.3700
1.3558
HN
O
CO2Et
Se
10
9
8
7
6
5
4
3
2
1
ppm
1.00
1.09
1.16
1.15
2.15
3.53
3.29
3.34

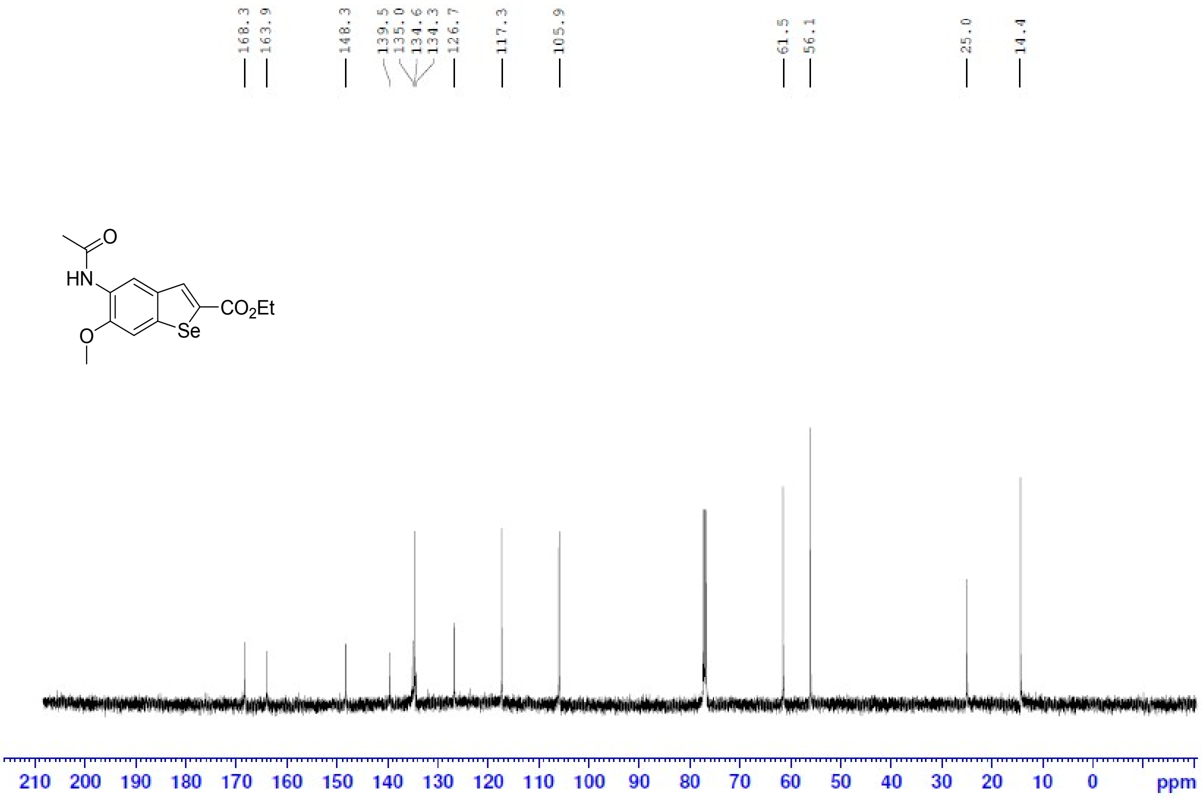

168.3
163.9
148.3
139.5
135.0
134.6
134.3
126.7
117.3
105.9
61.5
56.1
25.0
14.4
HN
O
CO2Et
Se
210
200
190
180
170
160
150
140
130
120
110
100
90
80
70
60
50
40
30
20
10
0
ppm

Compound **11**

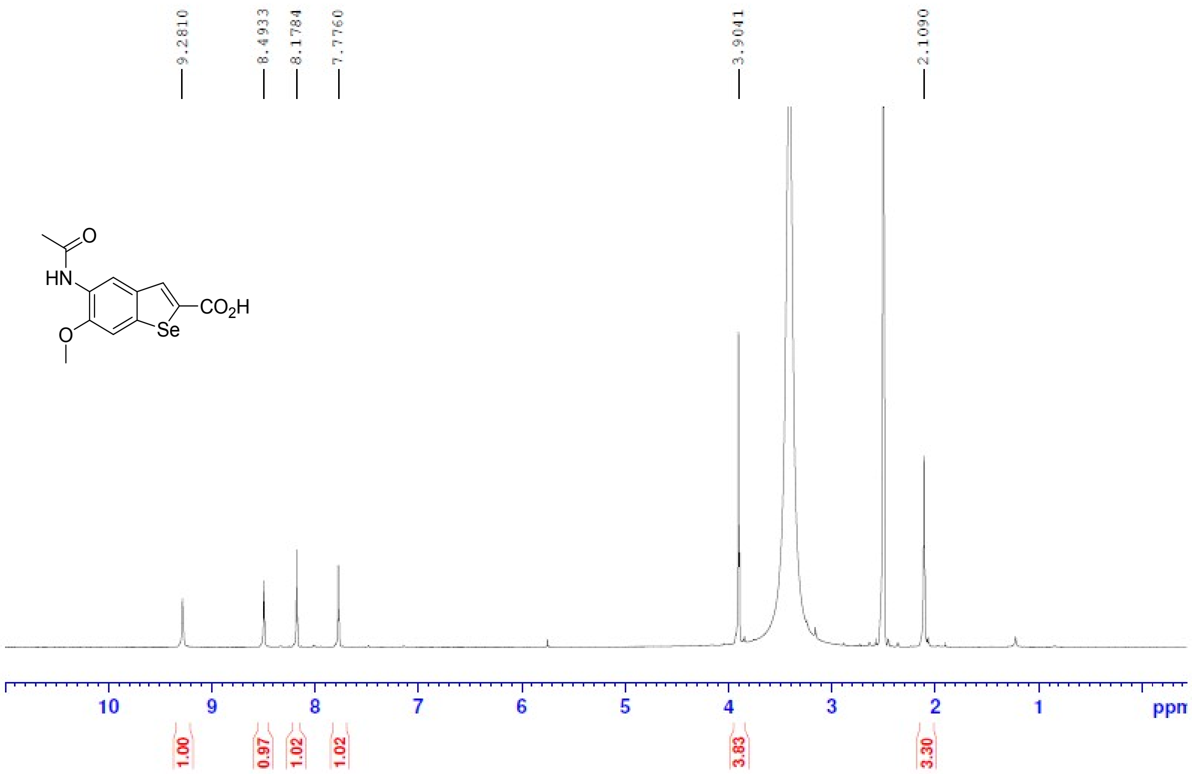

9.2810
8.4933
8.1784
7.7760
3.9041
2.1090
HN
O
CO2H
Se
10
9
8
7
6
5
4
3
2
1
ppm
1.00
0.97
1.02
1.02
3.83
3.30

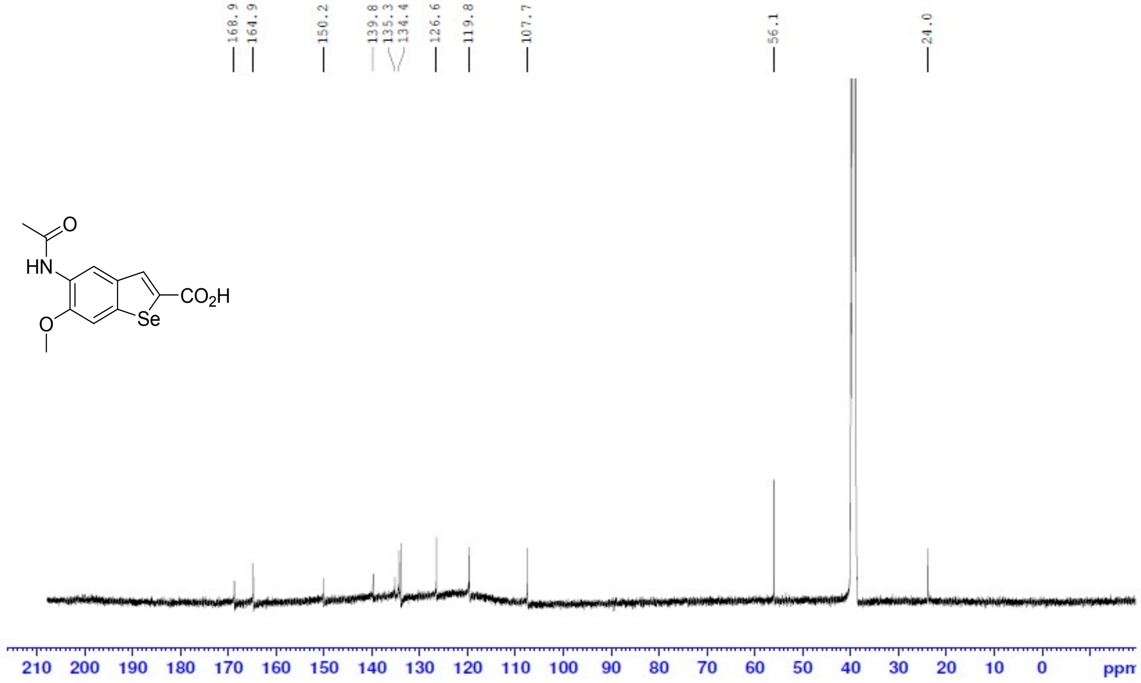

168.9
164.9
150.2
139.8
135.3
134.4
126.6
119.8
107.7
56.1
24.0
HN
O
CO2H
Se
210
200
190
180
170
160
150
140
130
120
110
100
90
80
70
60
50
40
30
20
10
0
ppm

Ester Intermediate of compound **13**

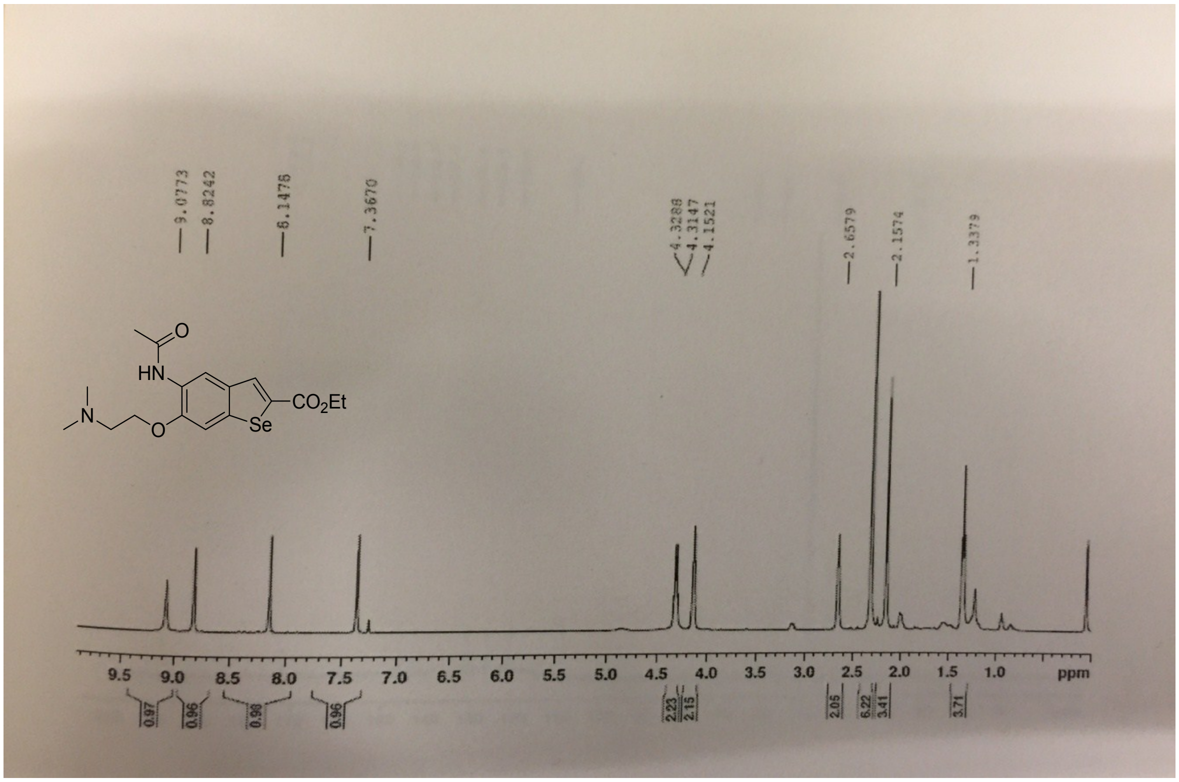

Compound **13**

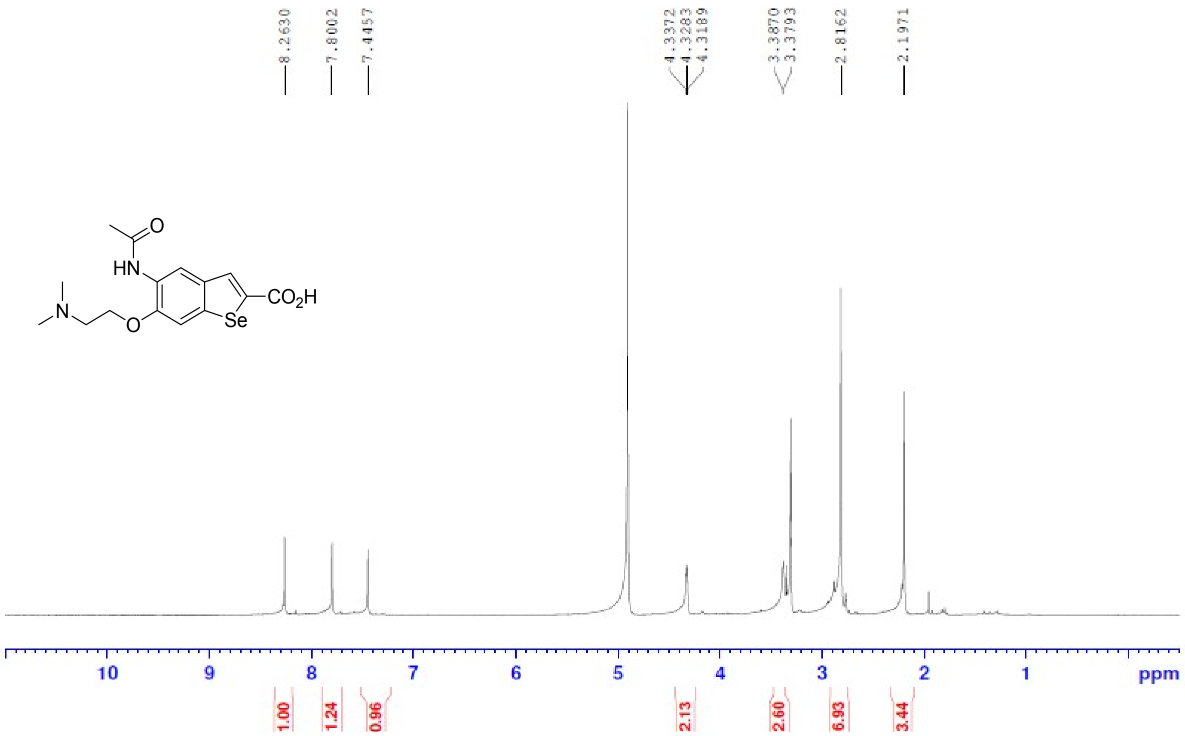

8.2630
7.8002
7.4457
4.3372
4.3283
4.3189
3.3870
3.3793
2.8162
2.1971
HN
O
N
O
Se
CO_2H
10
9
8
7
6
5
4
3
2
1
ppm
1.00
1.24
0.96
2.13
2.60
6.93
3.44

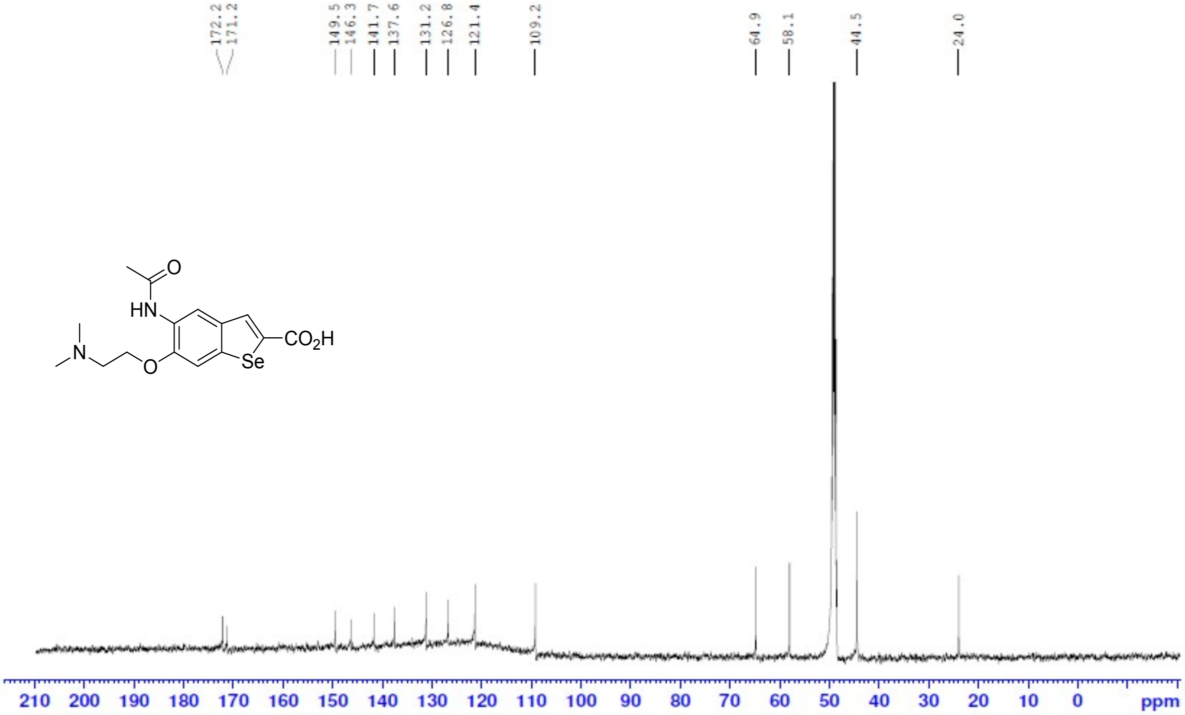

172.2
171.2
149.5
146.3
141.7
137.6
131.2
126.8
121.4
109.2
64.9
58.1
44.5
24.0
HN
O
N
O
Se
CO_2H
210
200
190
180
170
160
150
140
130
120
110
100
90
80
70
60
50
40
30
20
10
0
ppm

Ester intermediate of compound **14**

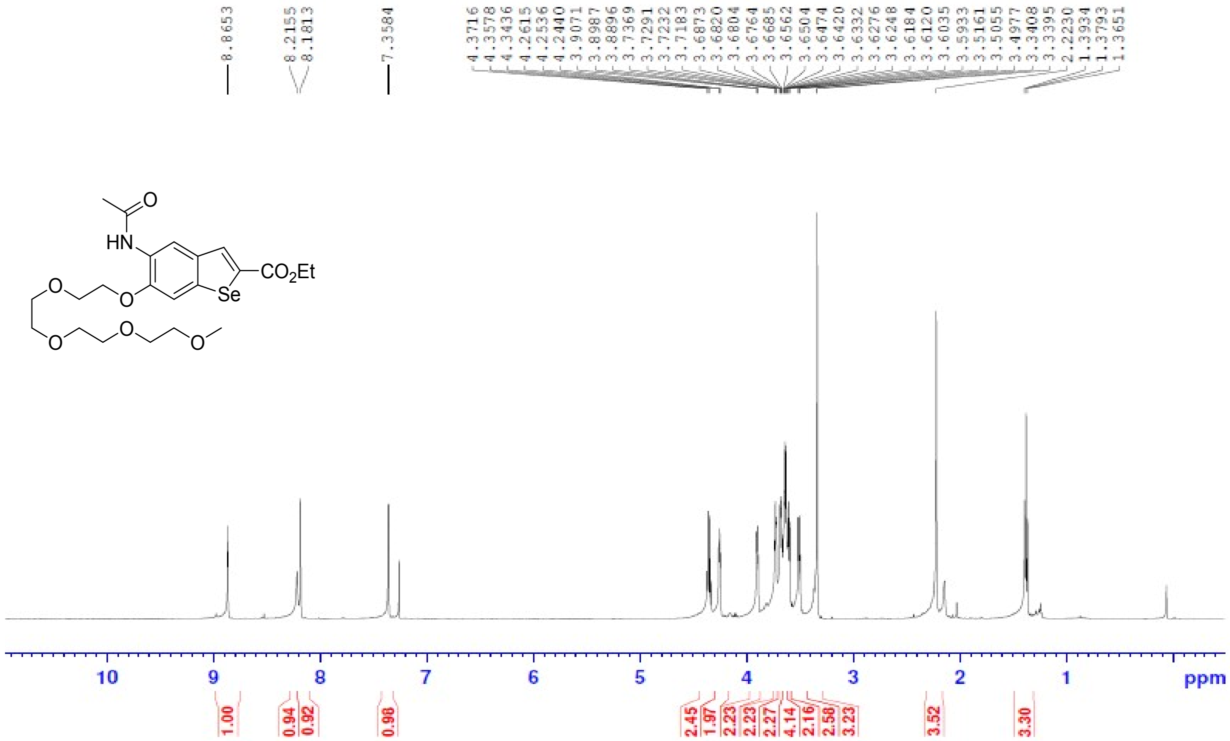

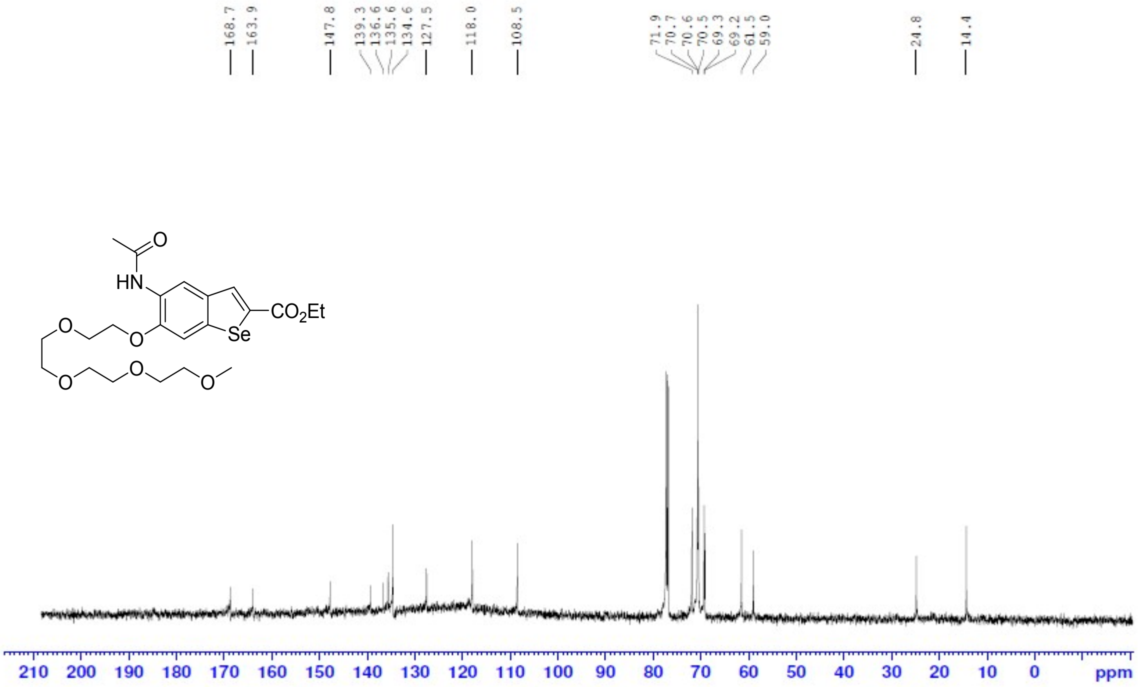

Compound **14**

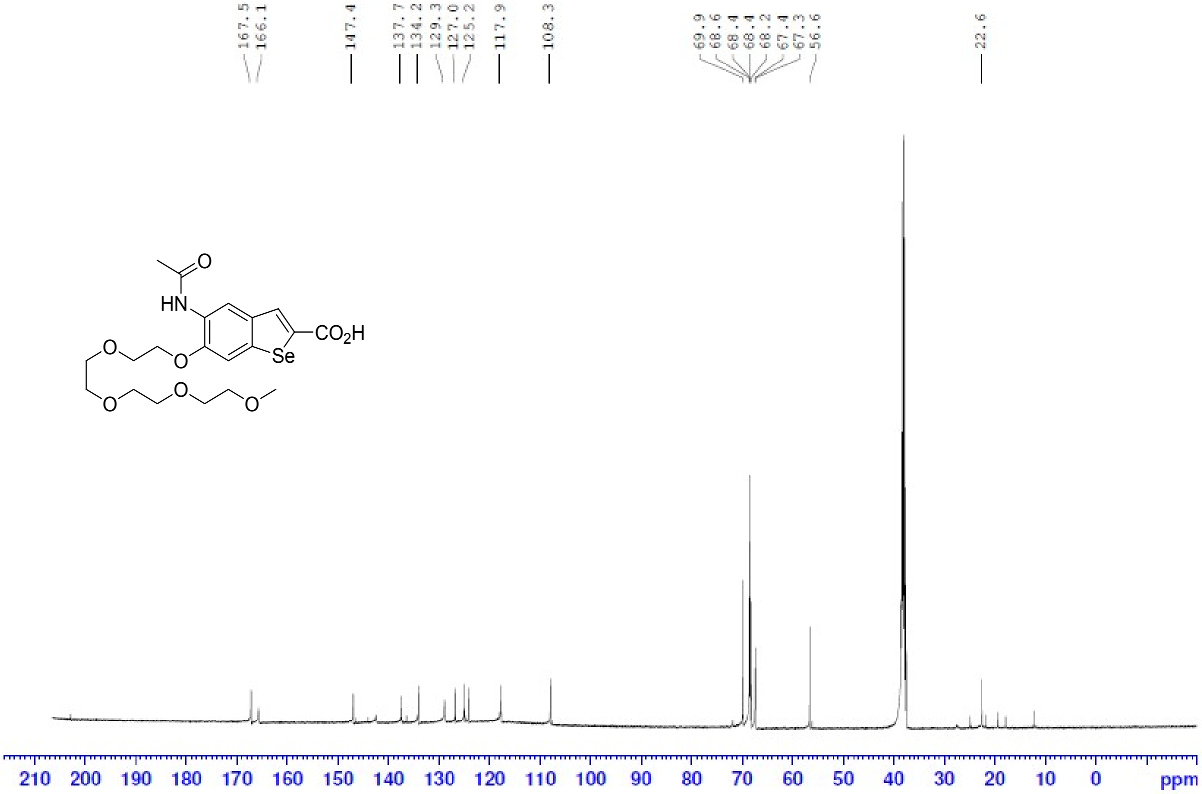

167.5
166.1
147.4
137.7
134.2
129.3
127.0
125.2
117.9
108.3
69.9
68.6
68.4
68.4
68.2
67.4
67.3
56.6
22.6
HN
O
CO2H
Se
O
O
O
O
O
210 200 190 180 170 160 150 140 130 120 110 100 90 80 70 60 50 40 30 20 10 0 ppm

Compound **18a**

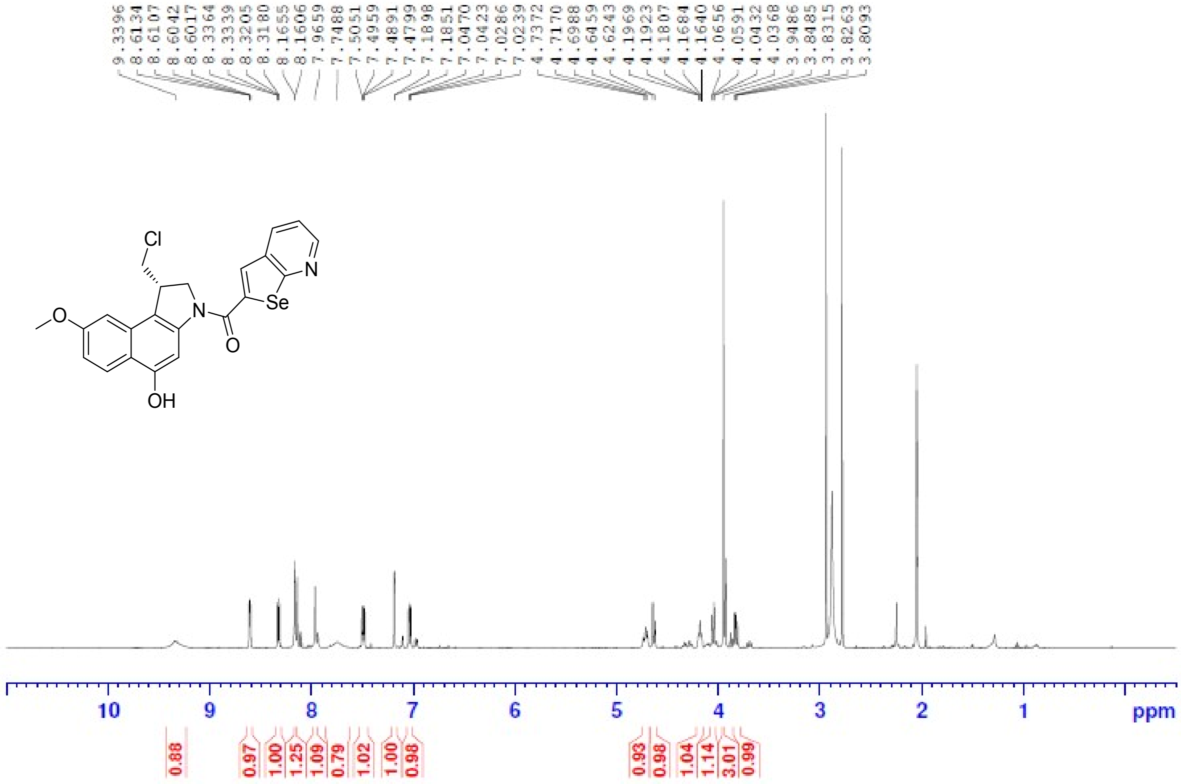

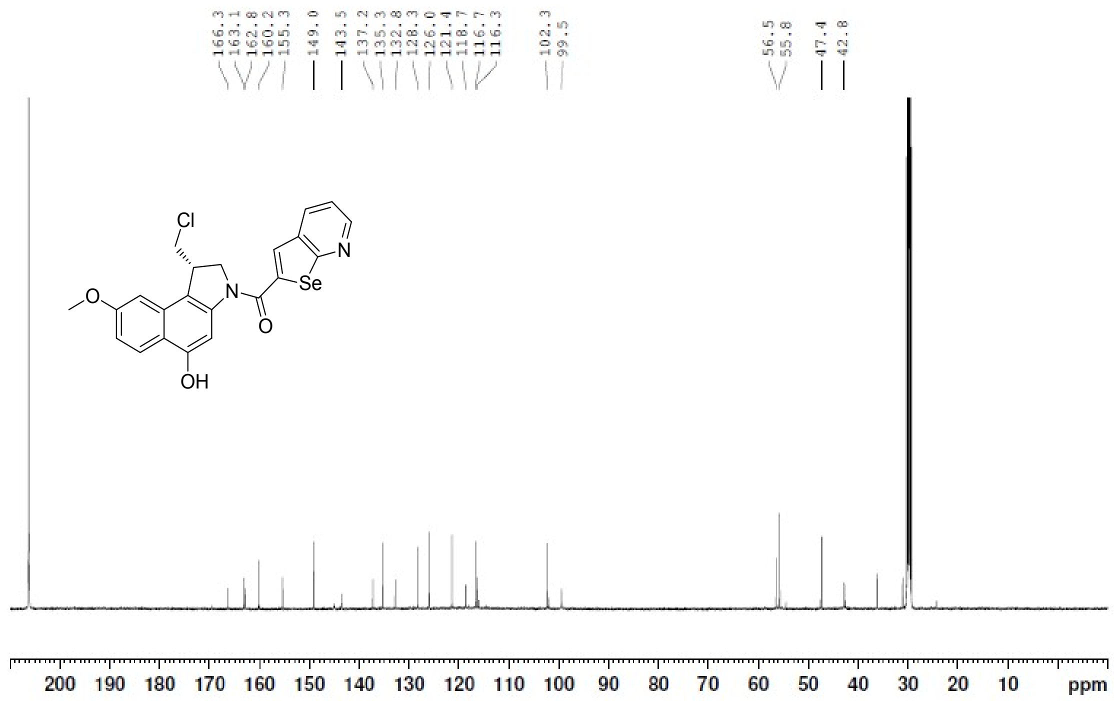

Compound **18b**

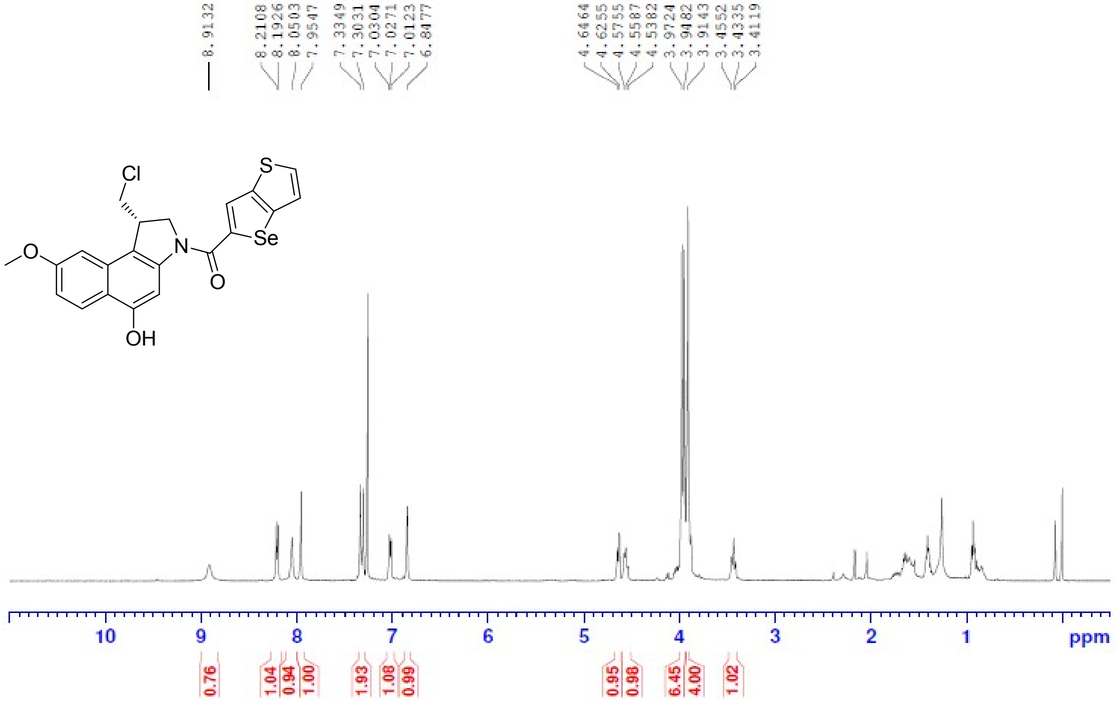

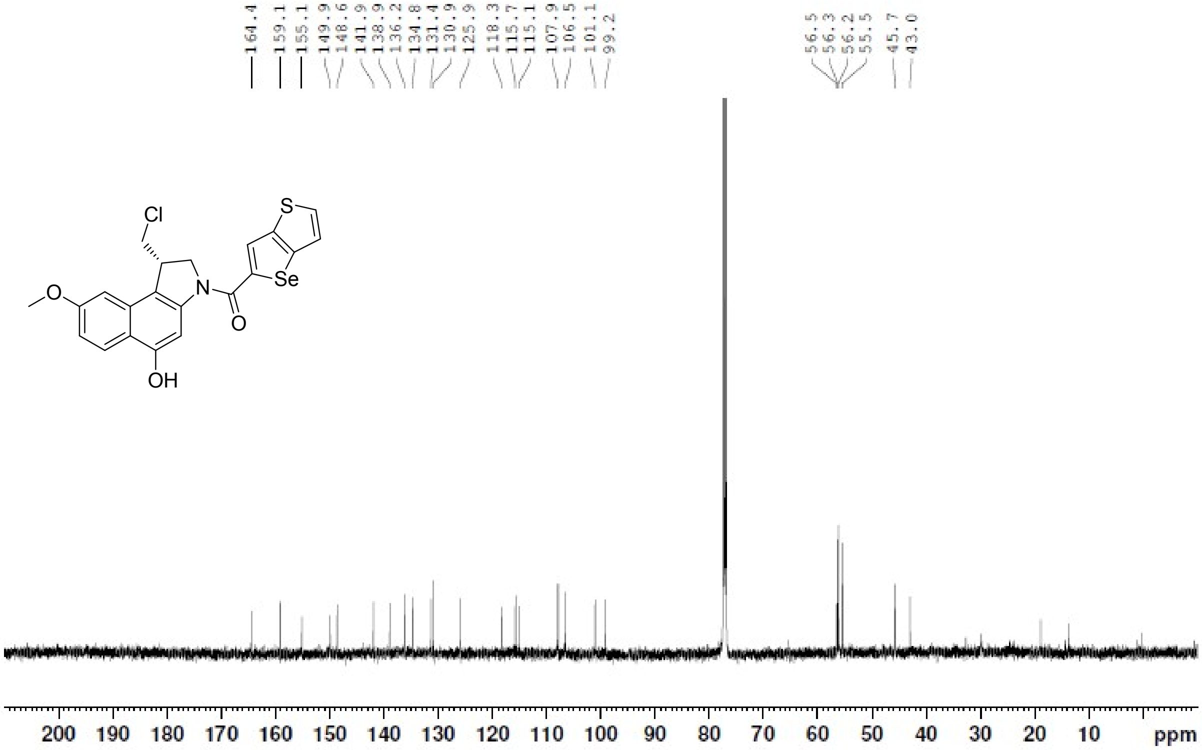

Compound **18c**

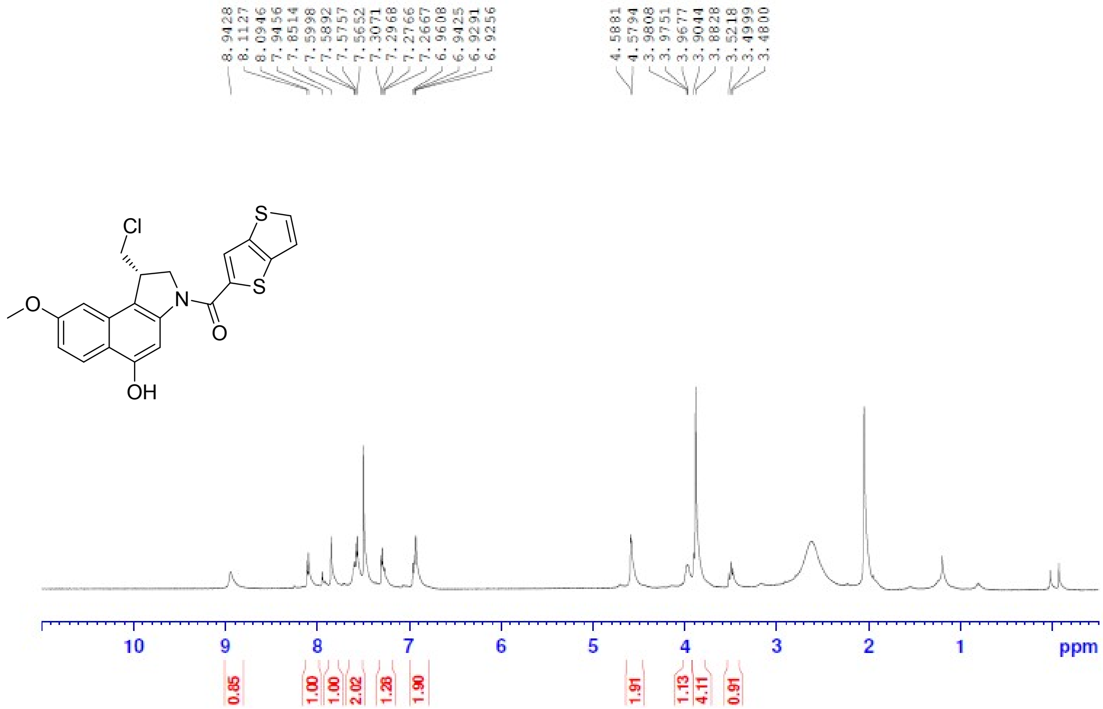

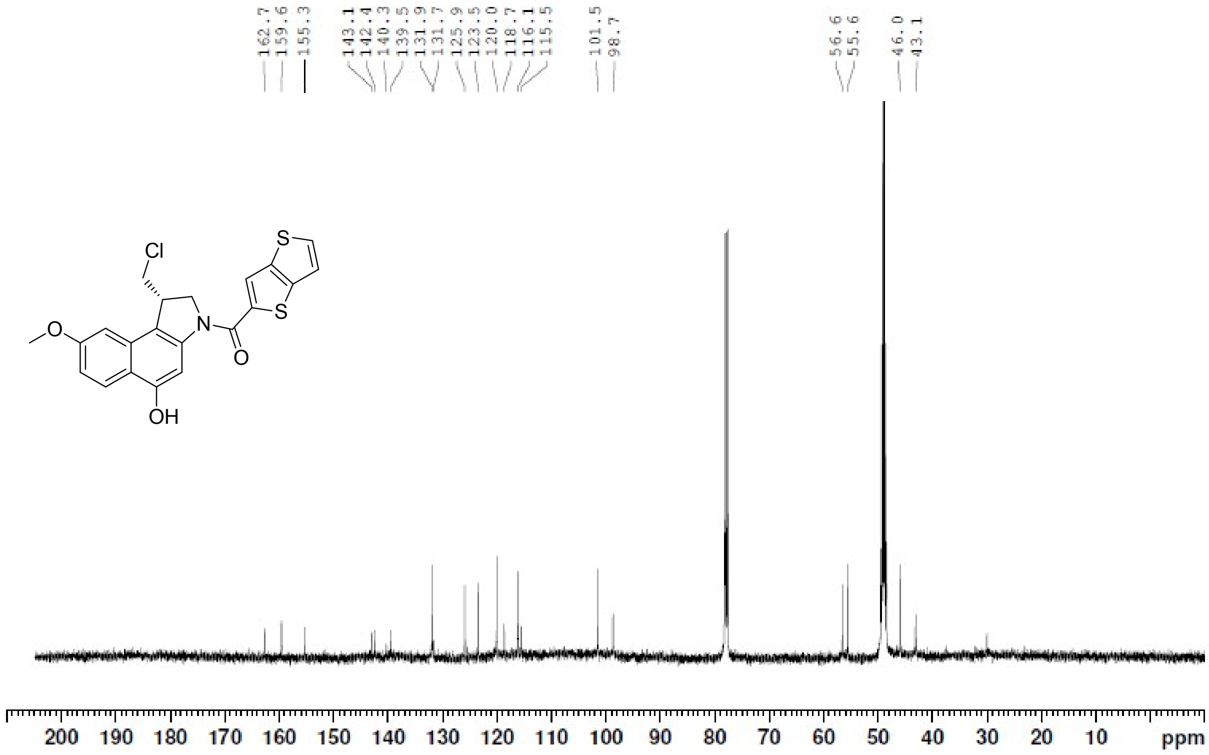

Compound **18d**

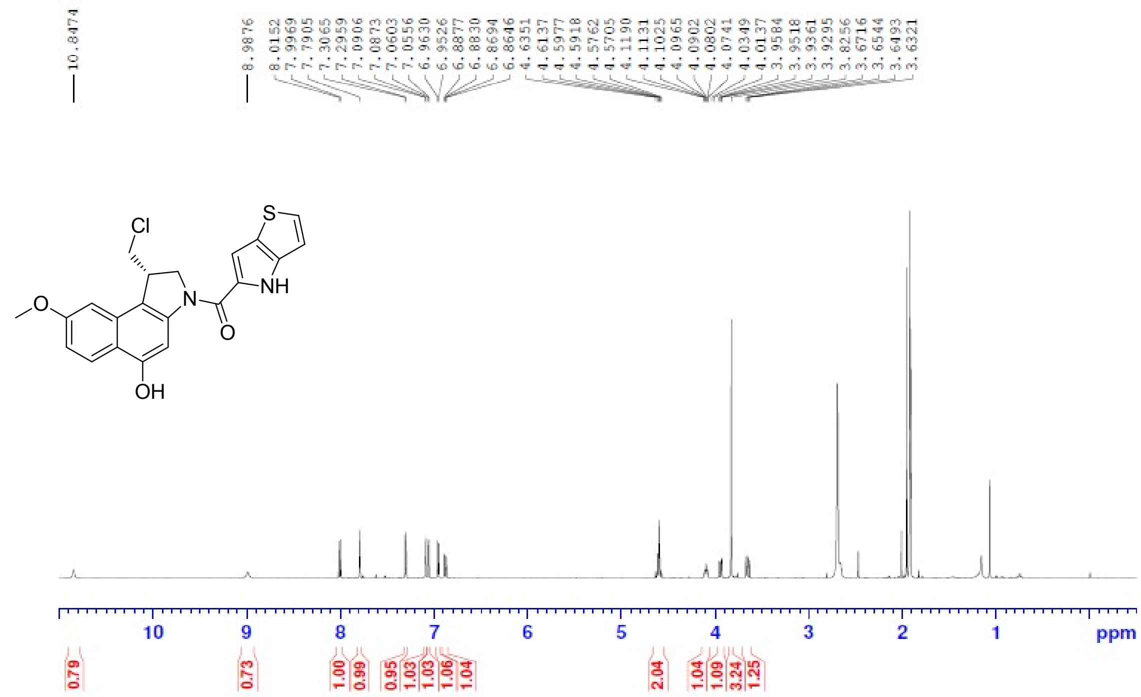

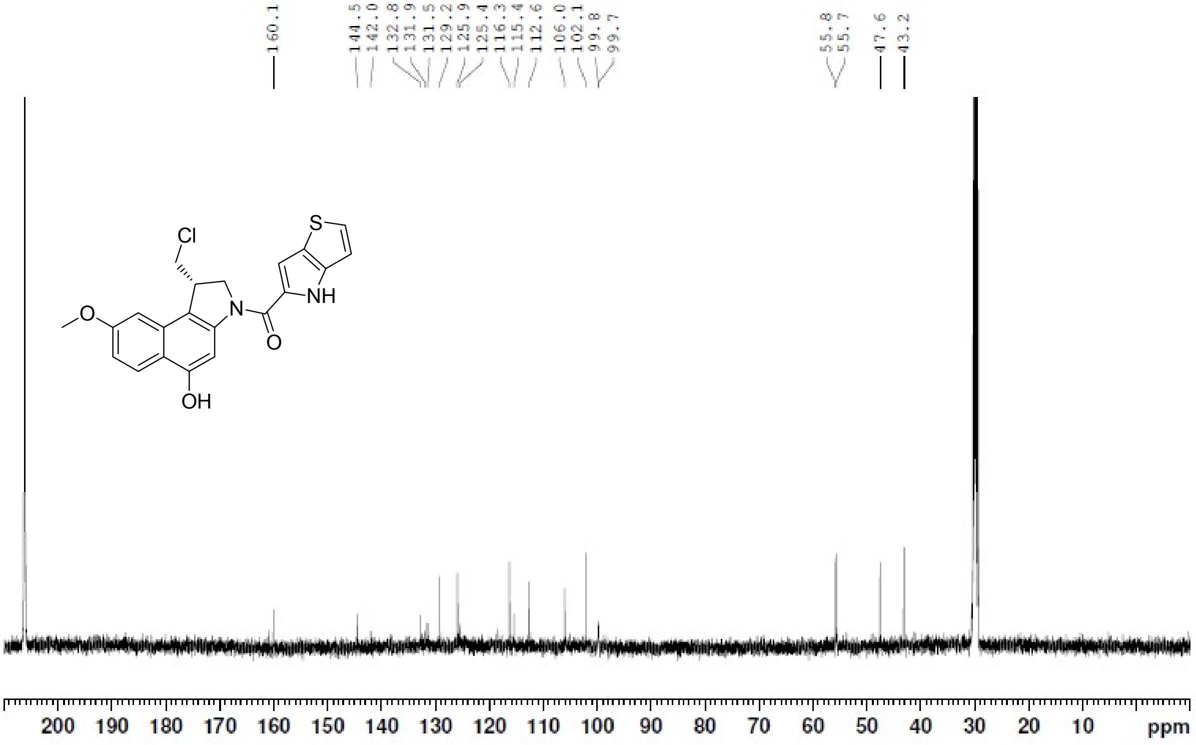

Compound **18e**

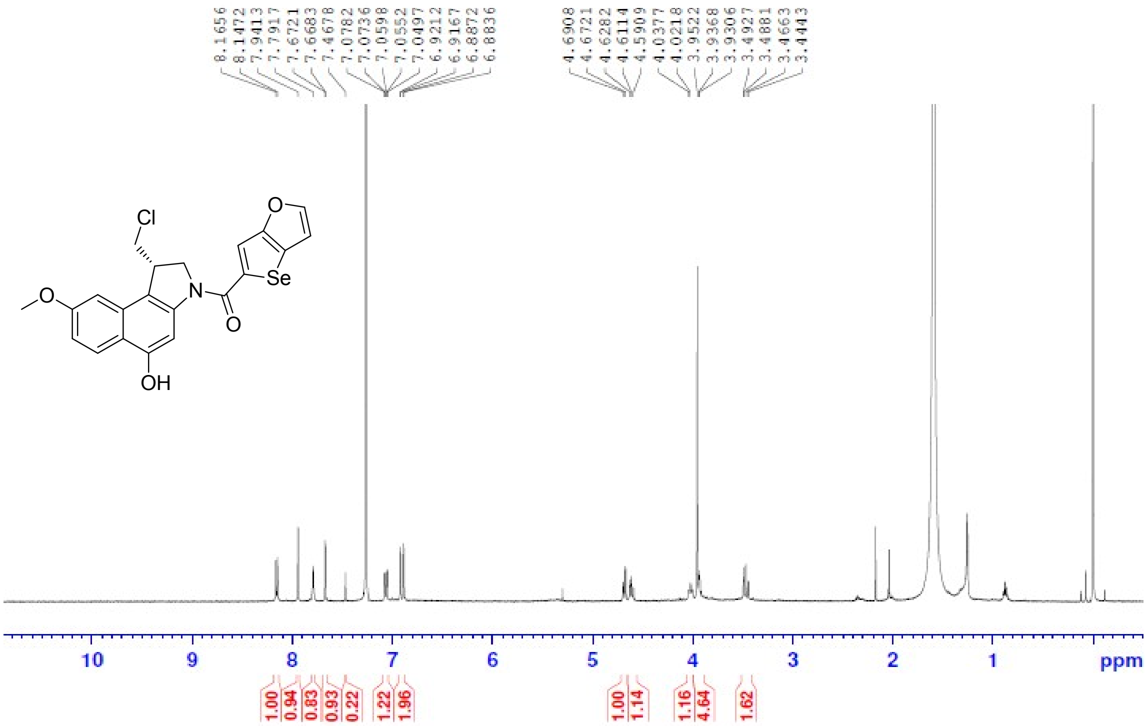

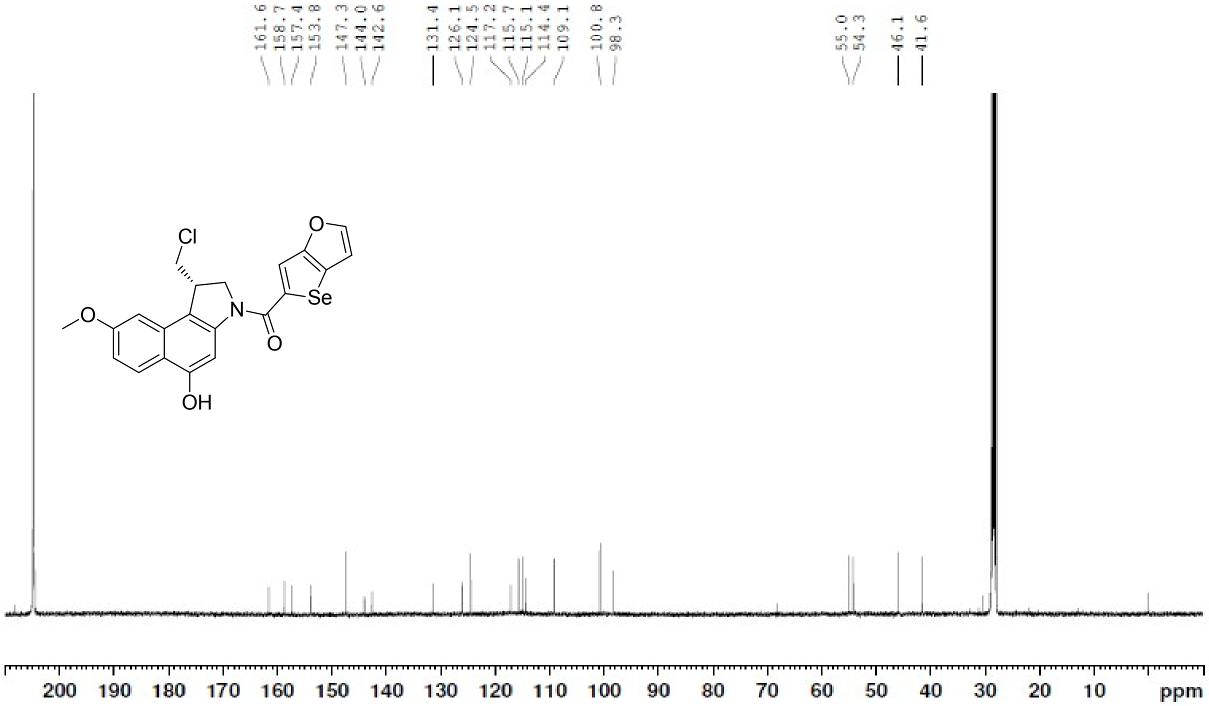

Compound **18f**

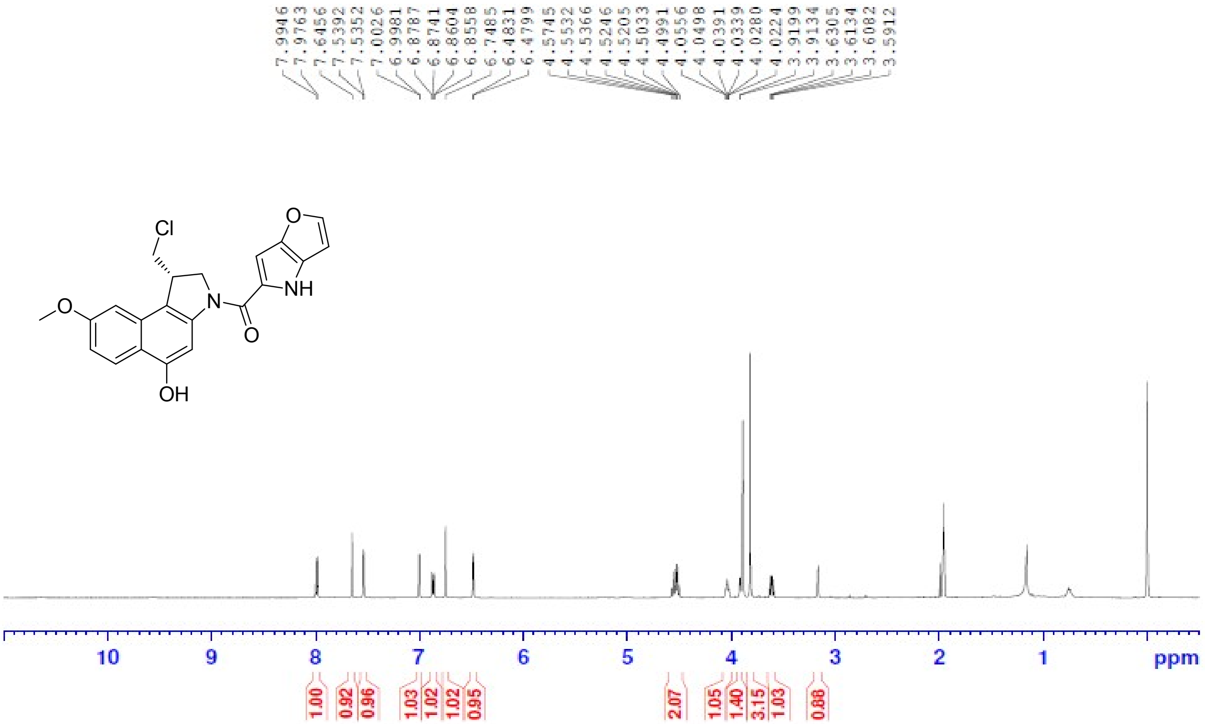

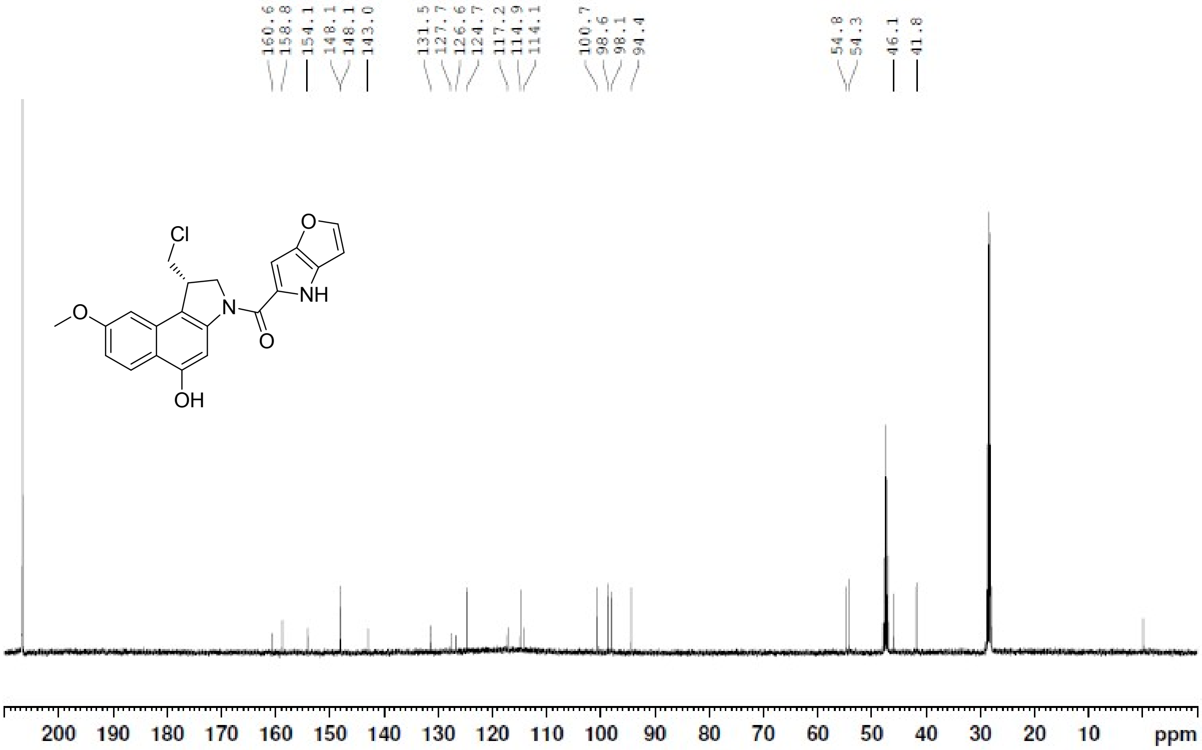

Compound **18g**

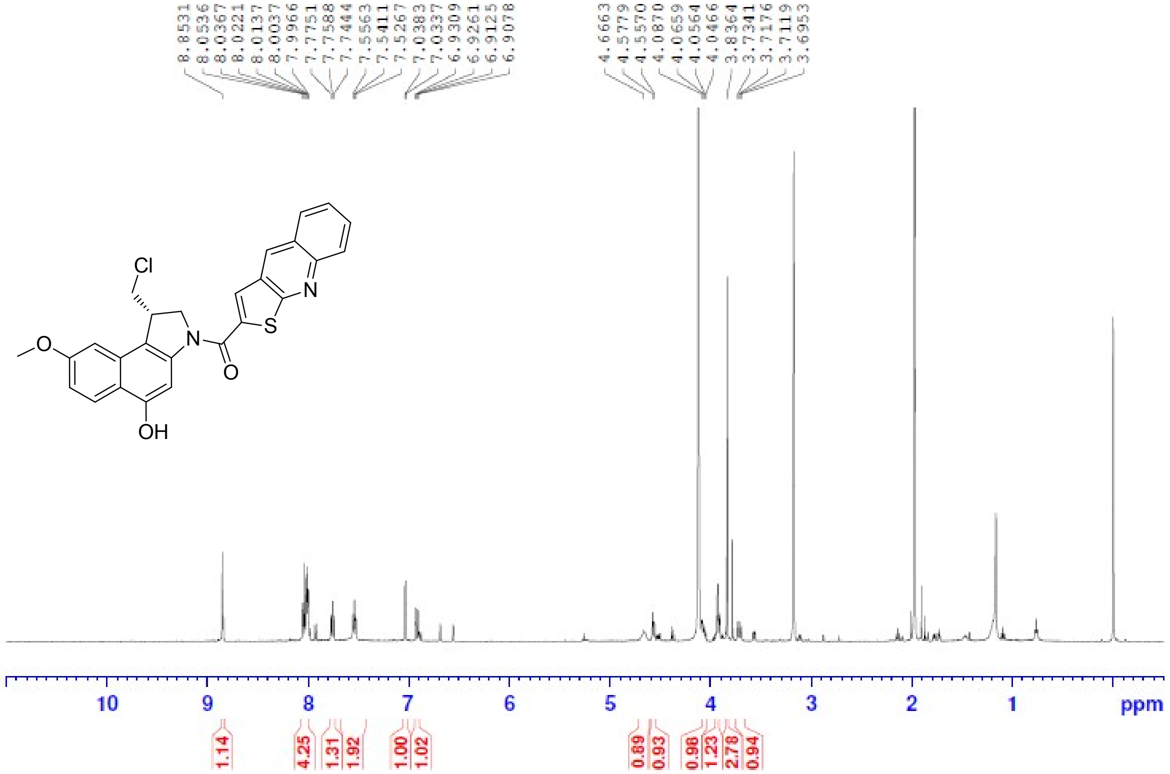

Compound **18h**

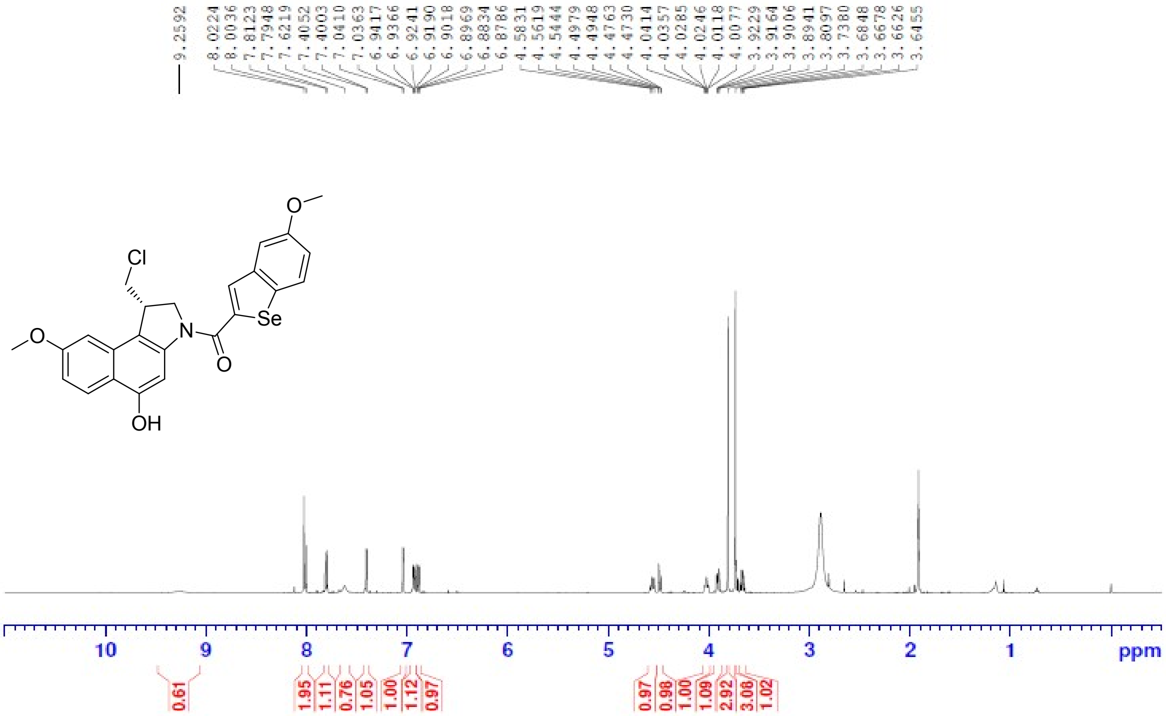

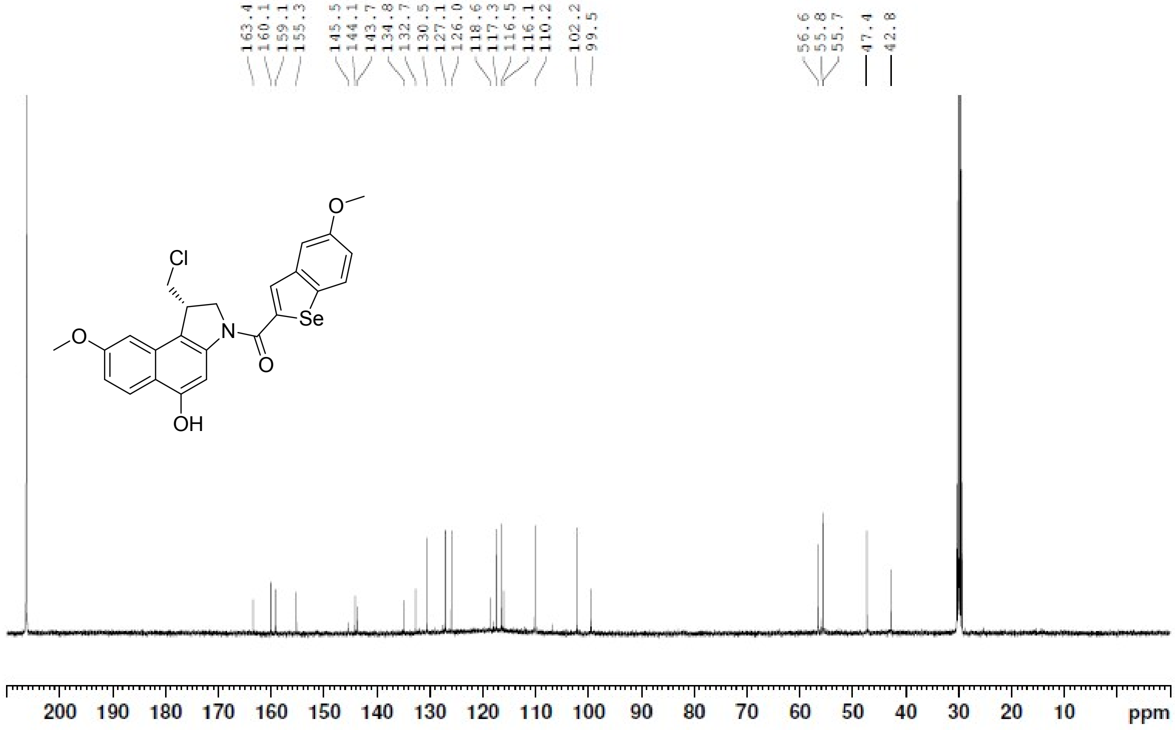

Compound **18i**

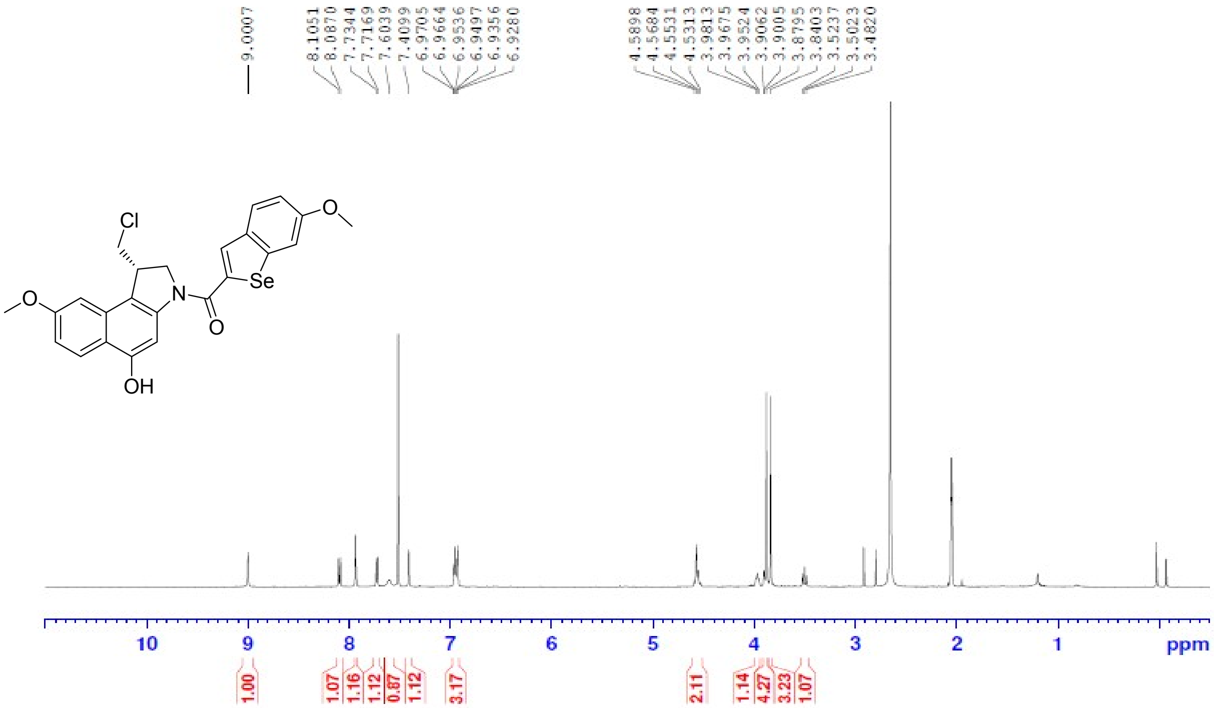

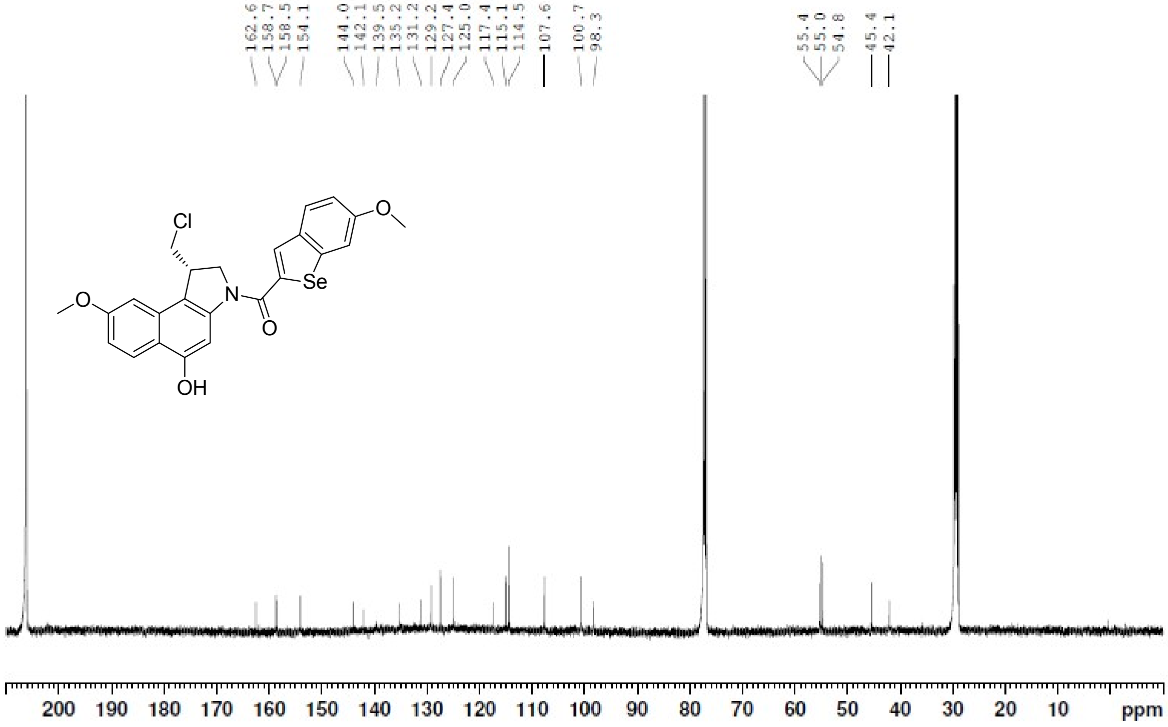

Compound **18j**

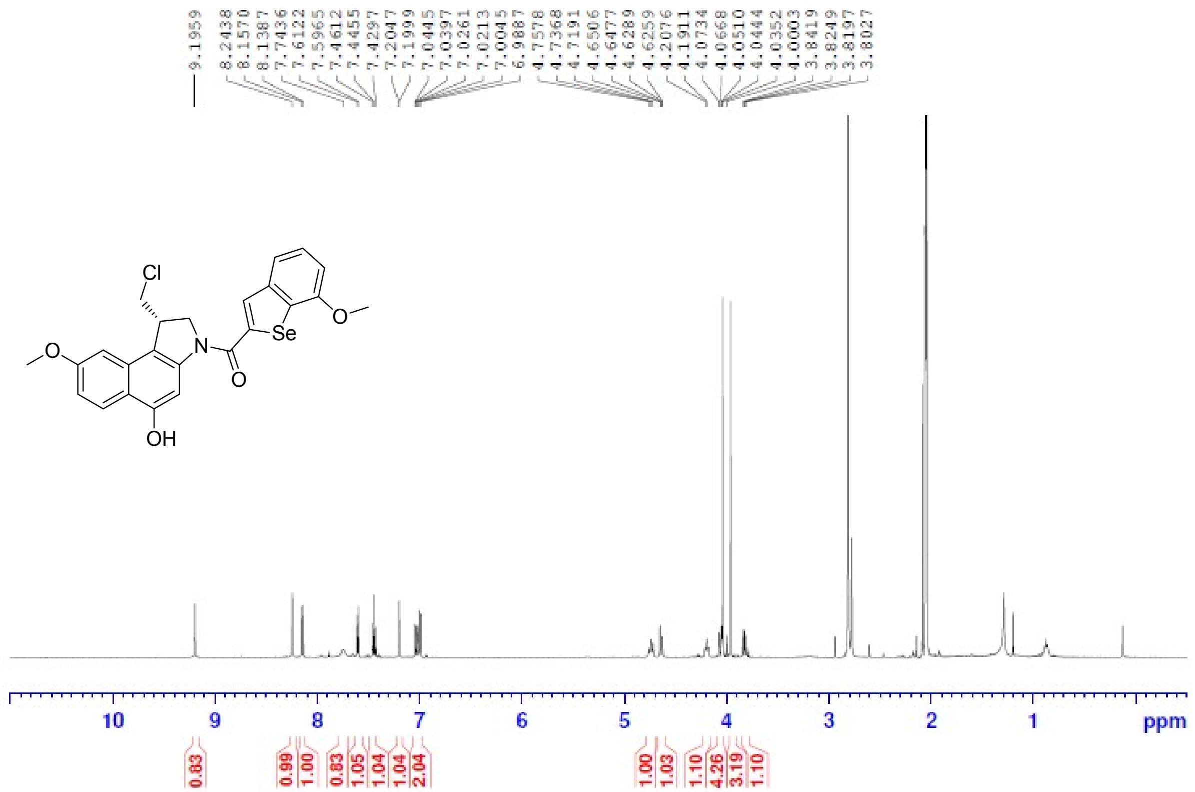

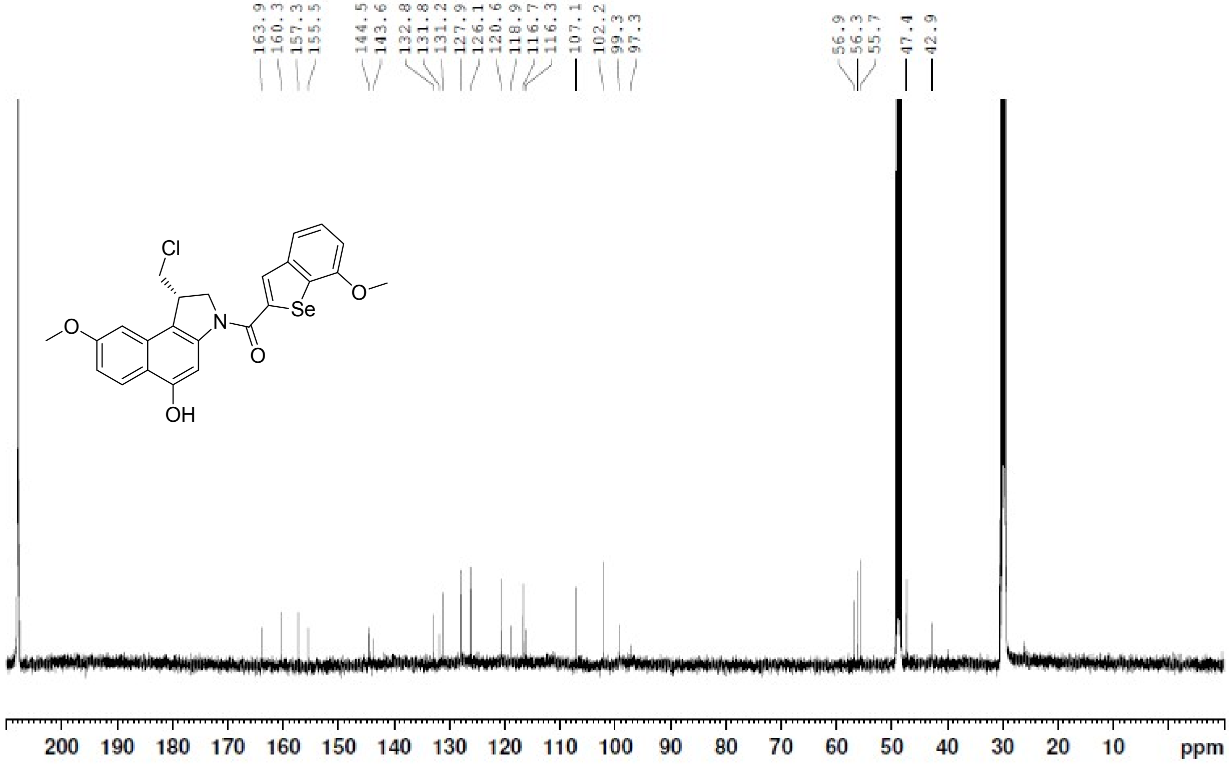

Compound **18k**

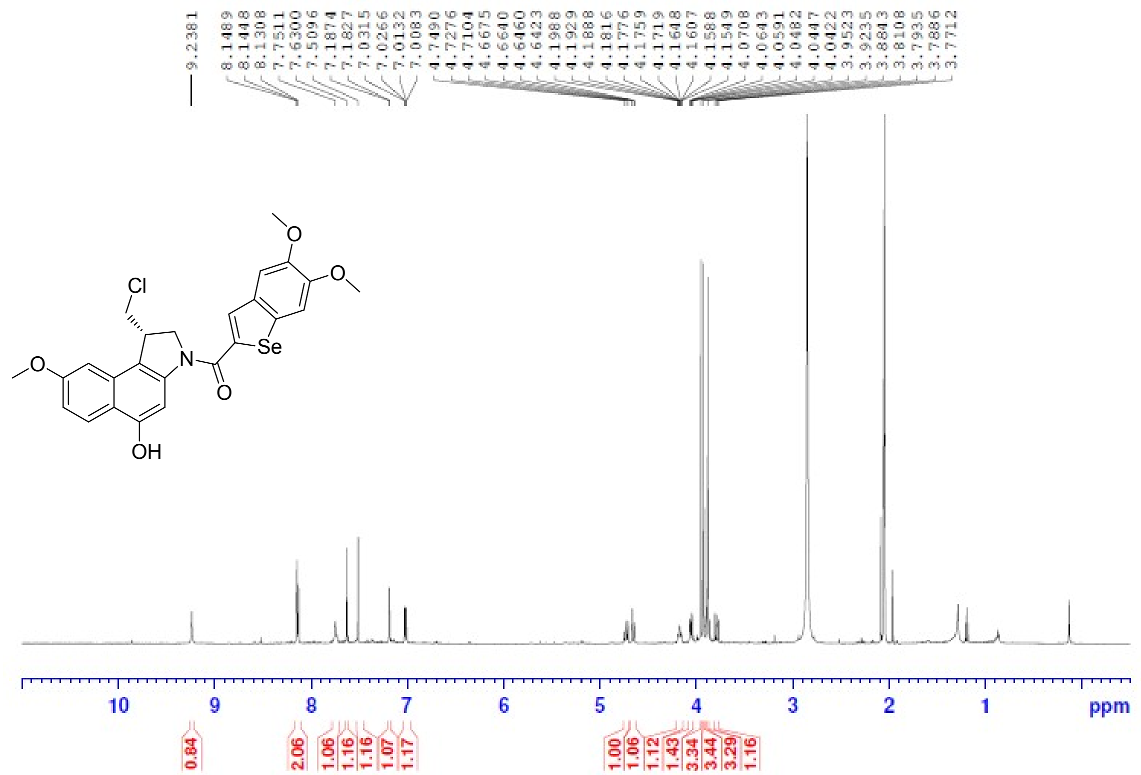

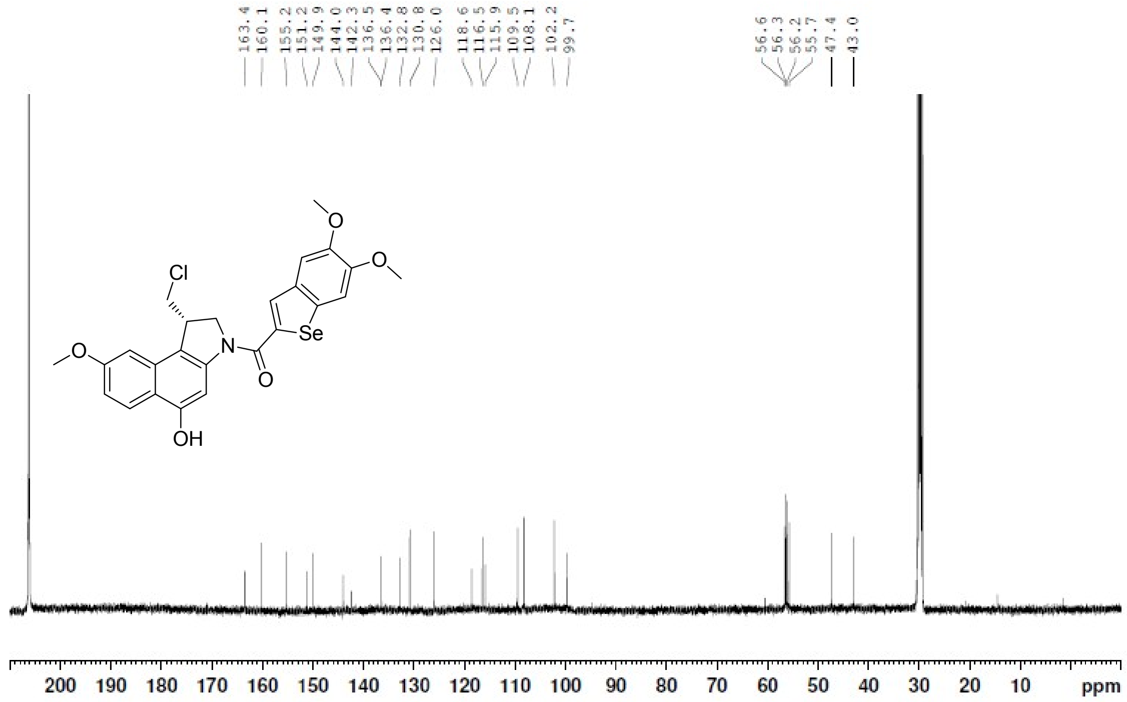

Compound **18l**

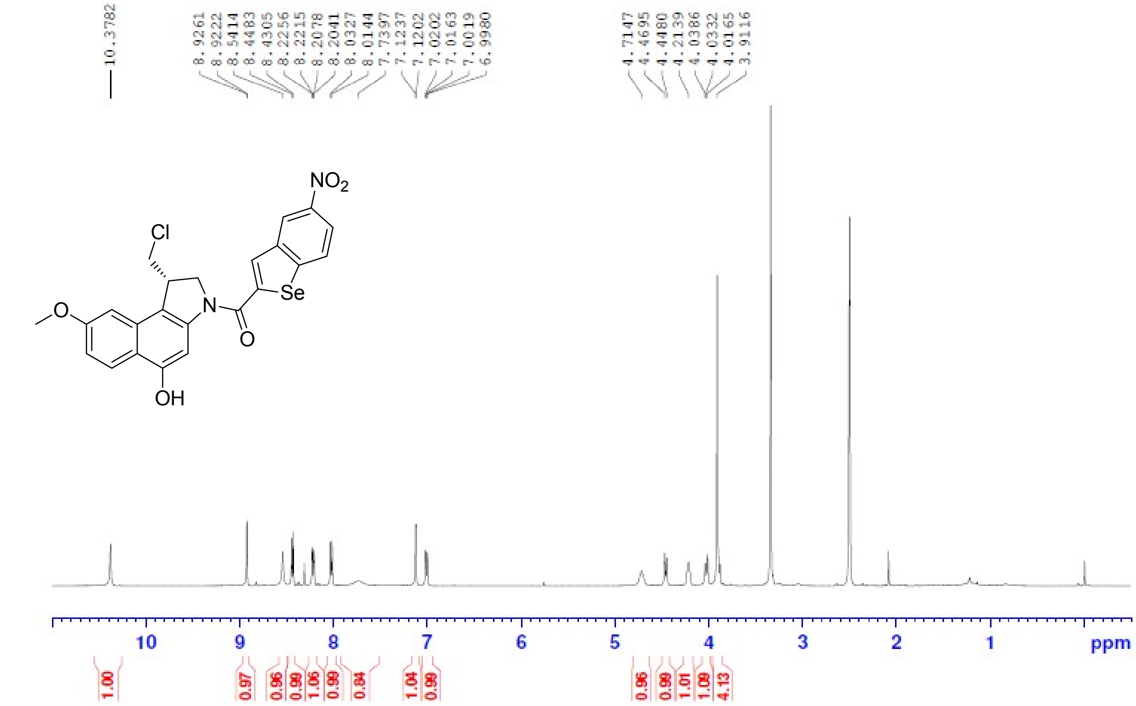

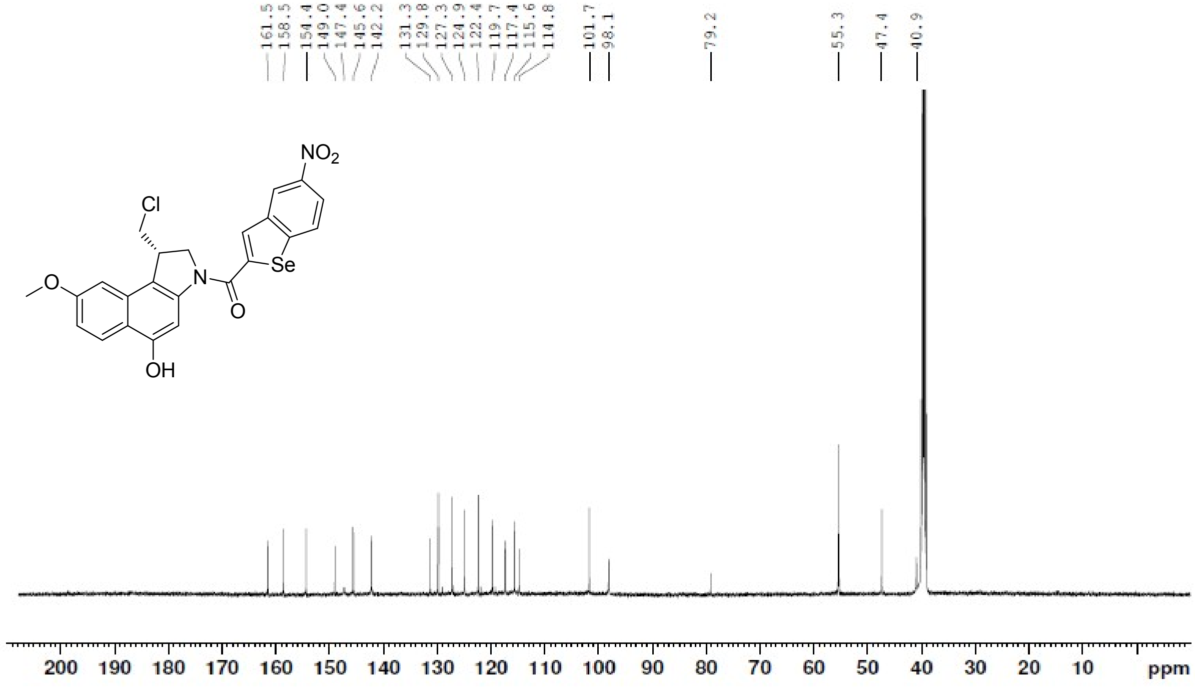

Compound **18m**

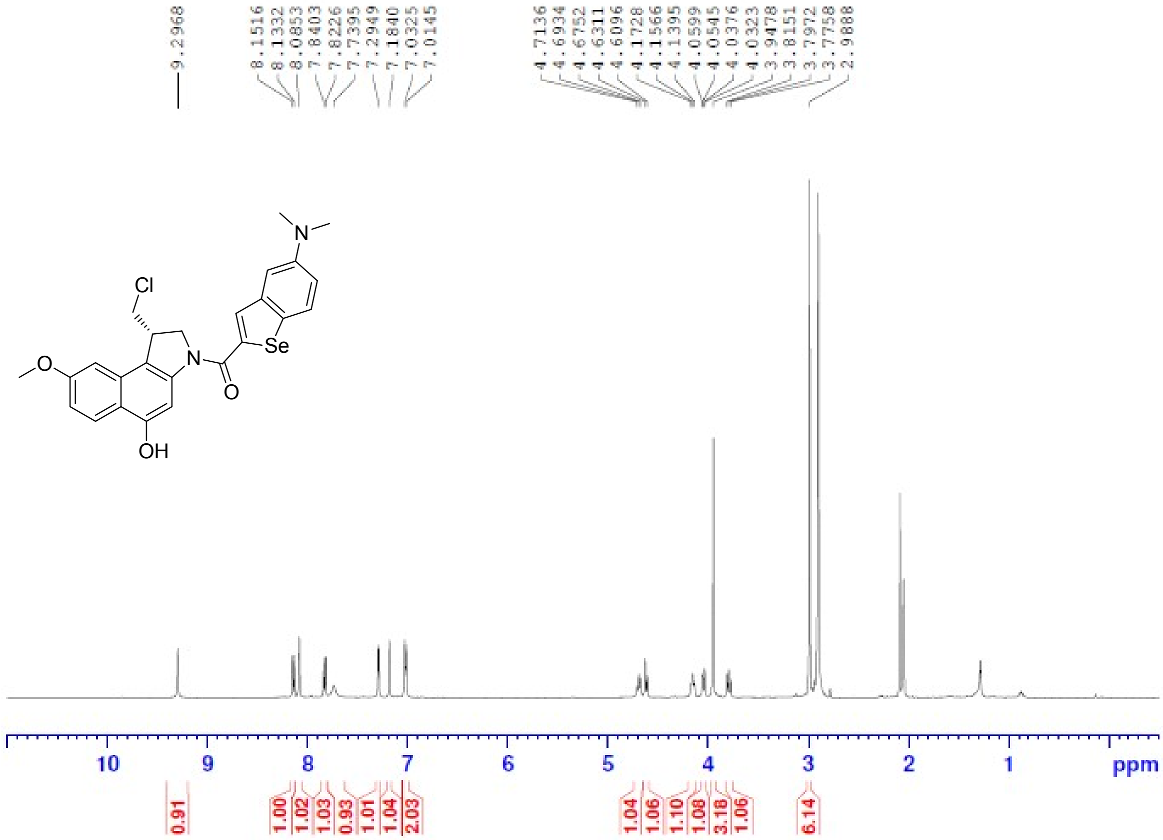

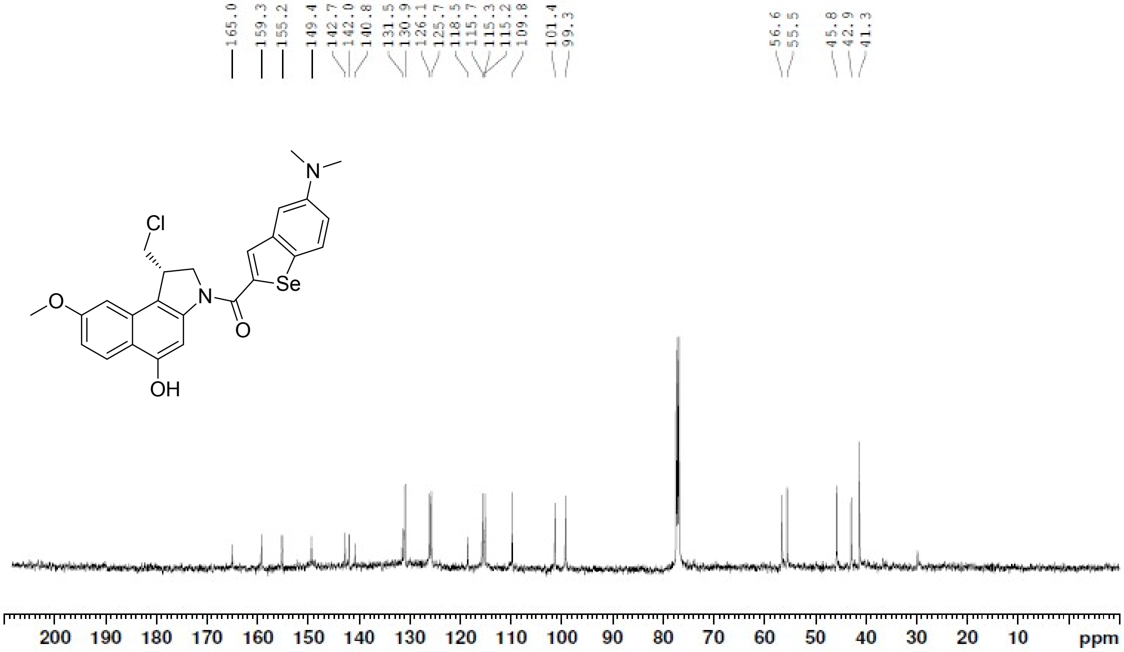

Compound **18n**

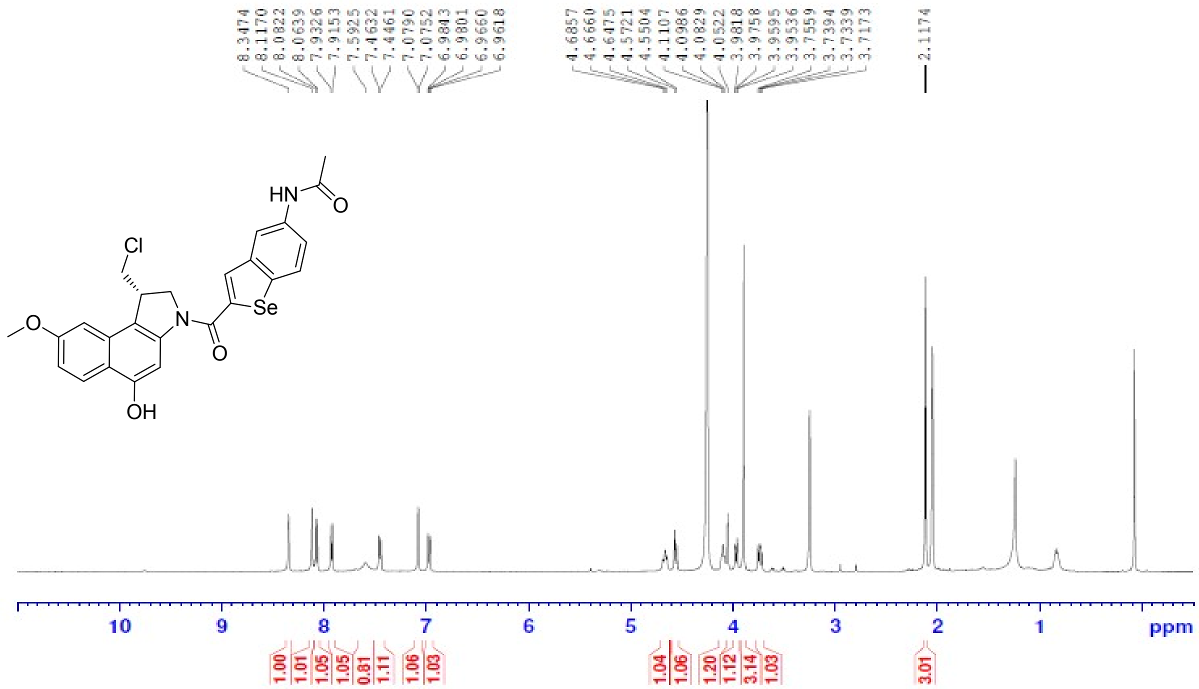

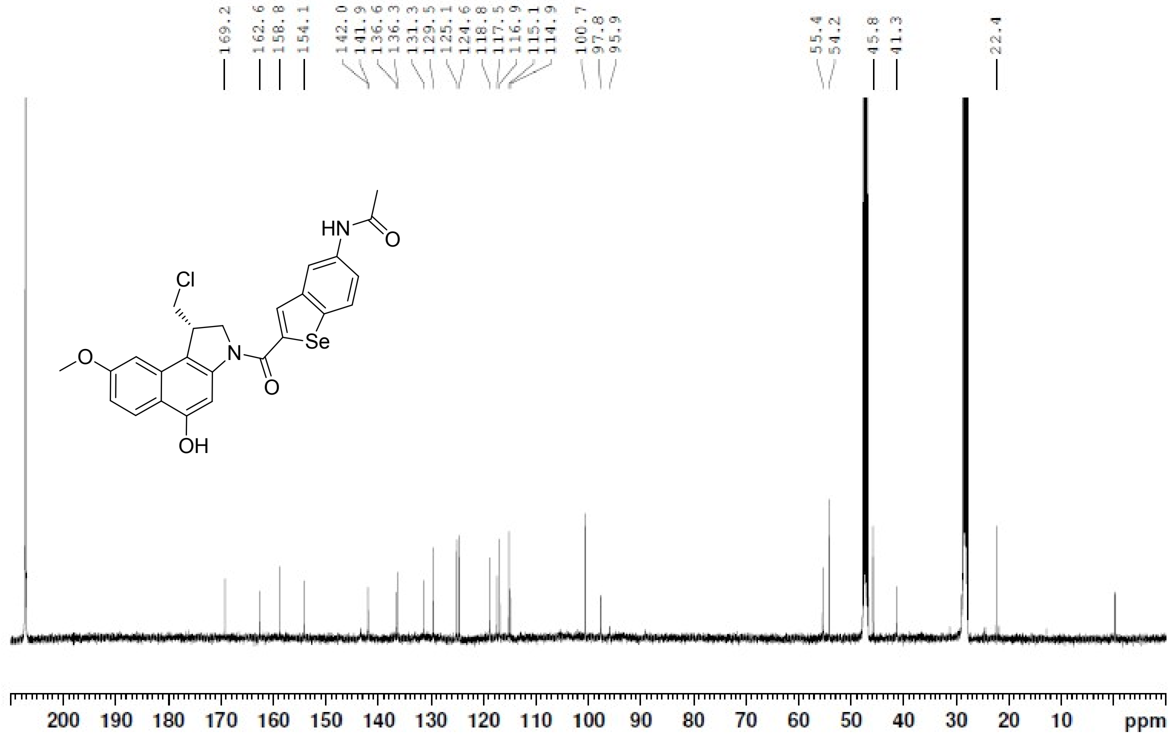

Compound **18o**

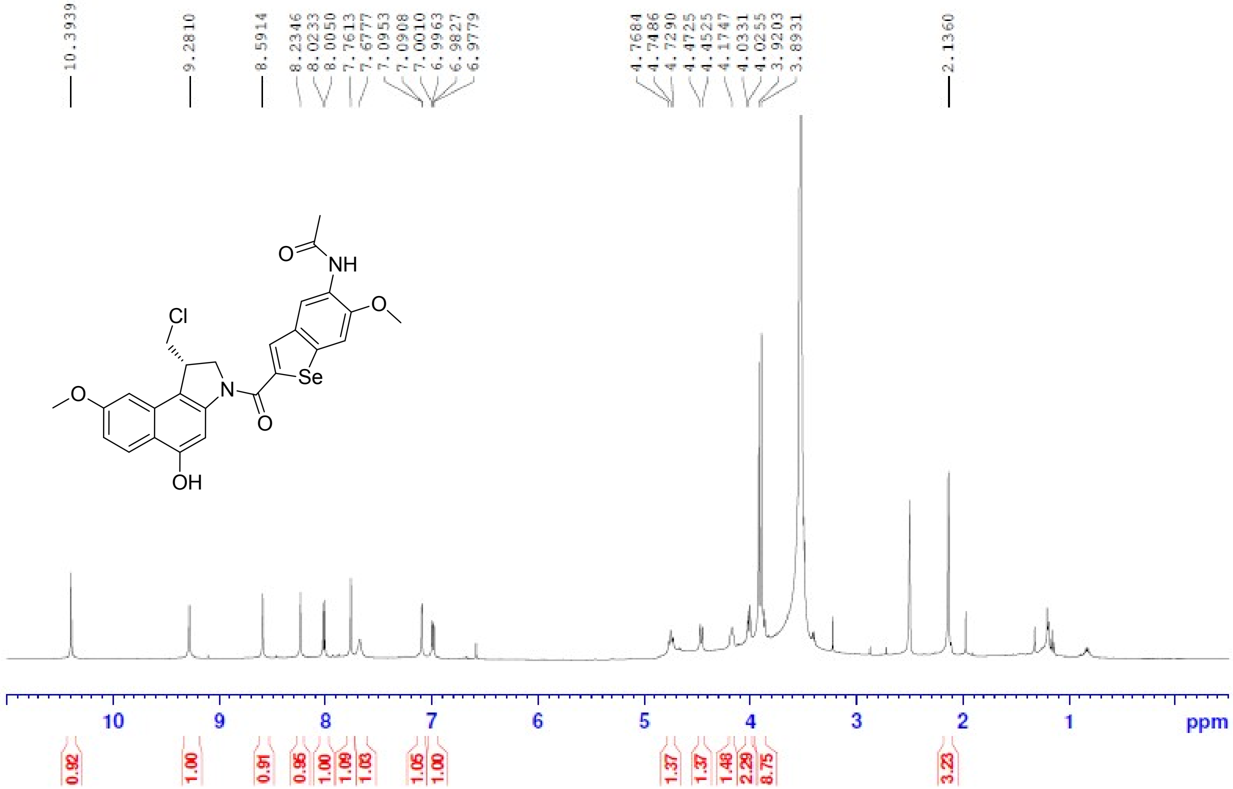

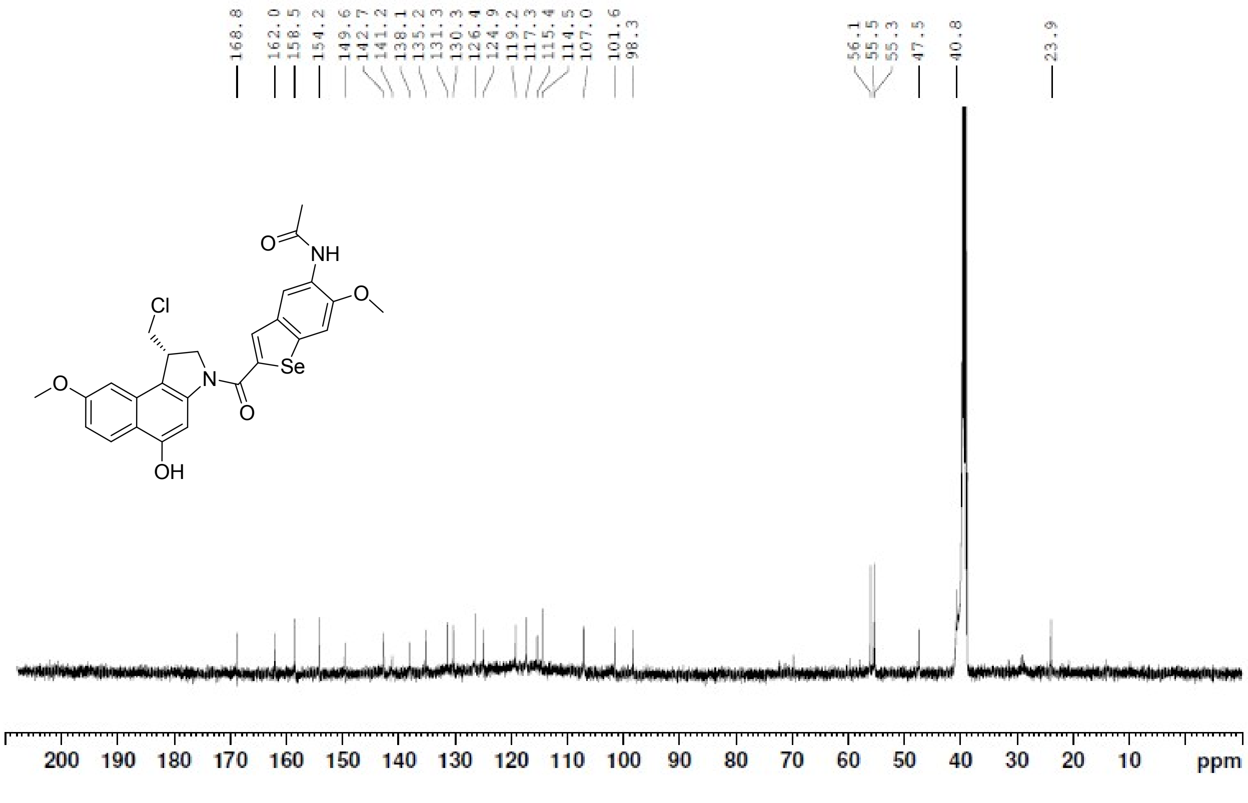

Compound **18p**

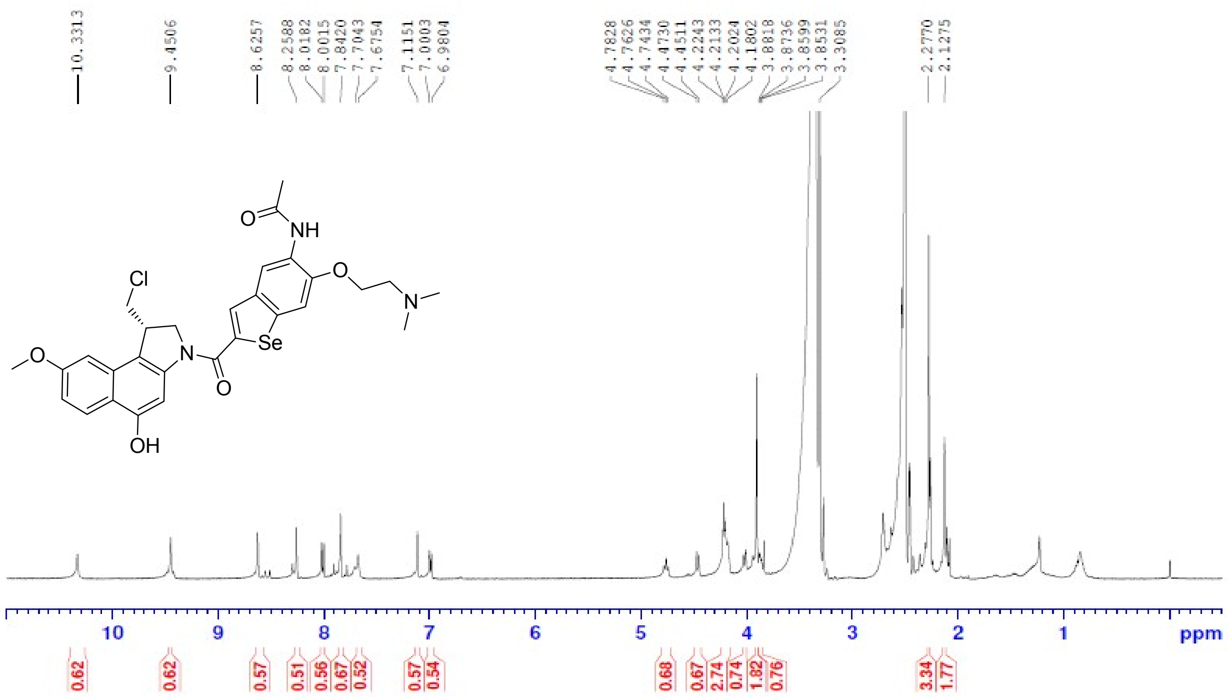

Compound **18q**

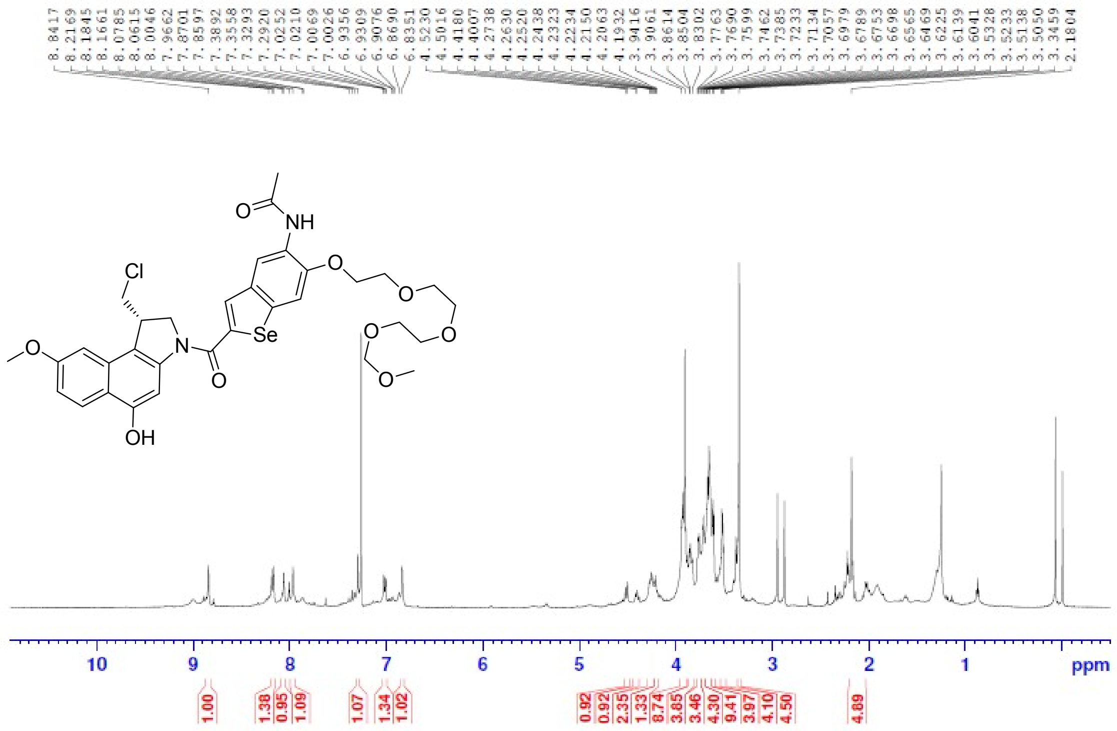

Compound **18r**

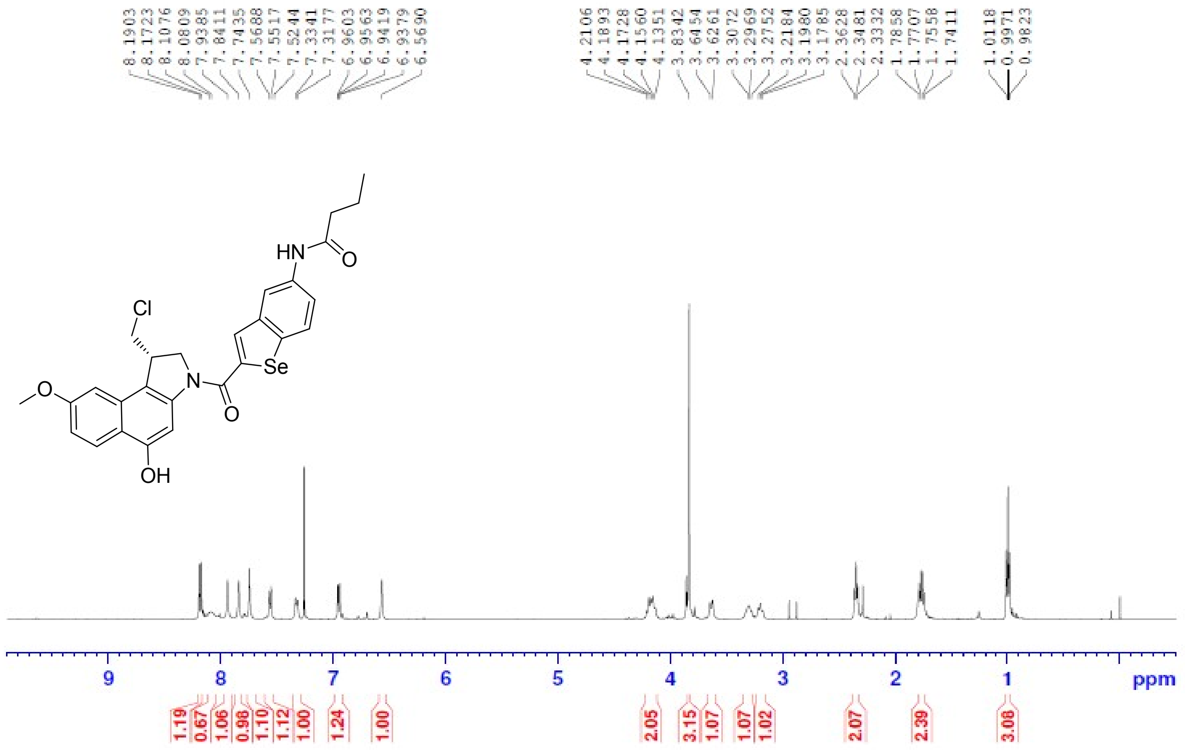

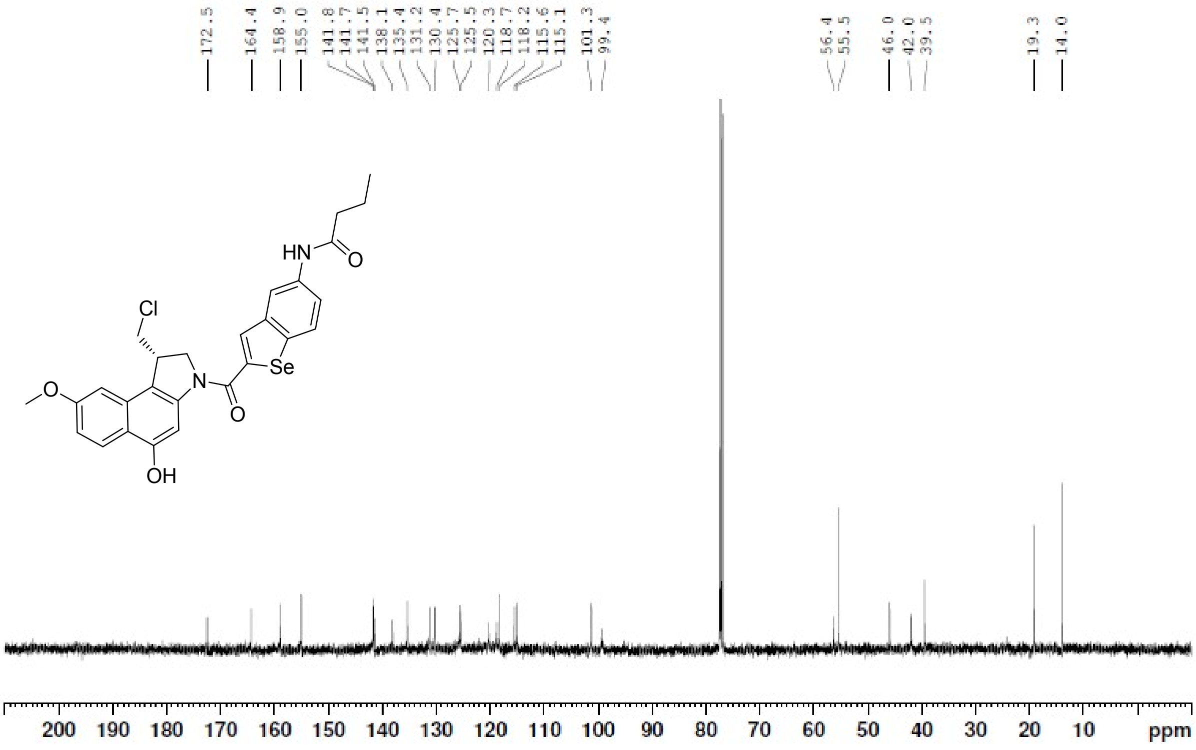

Compound **18s**

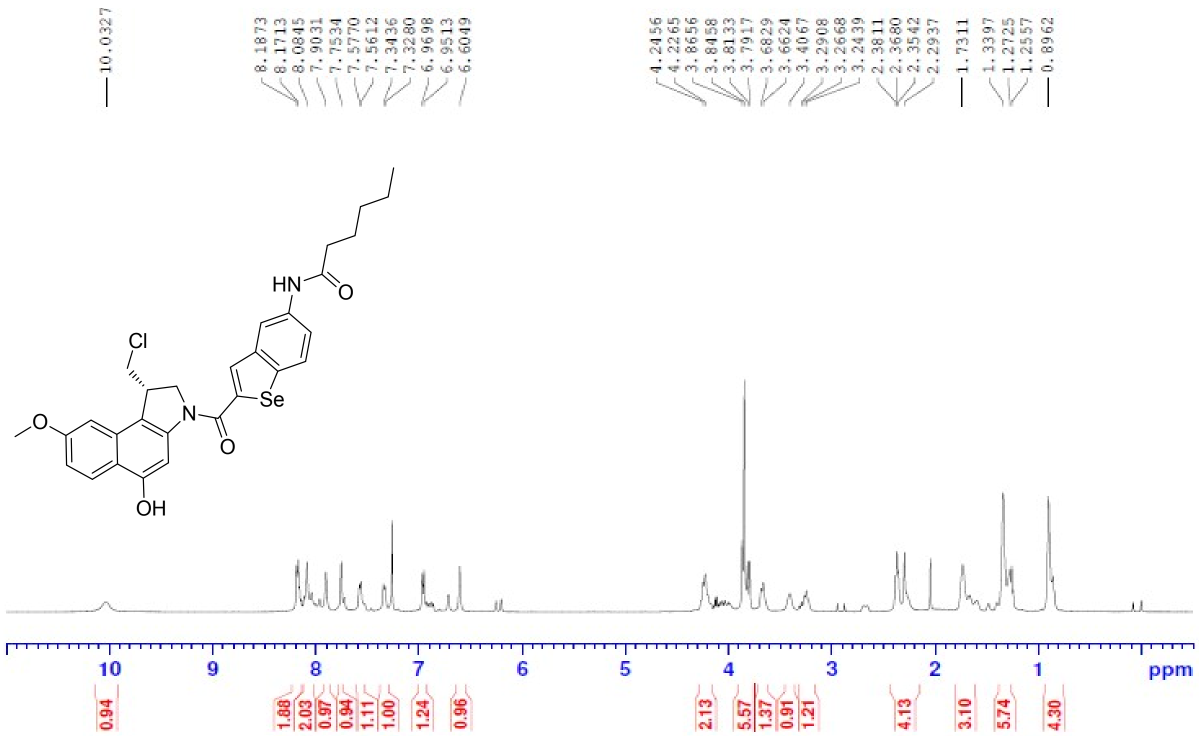

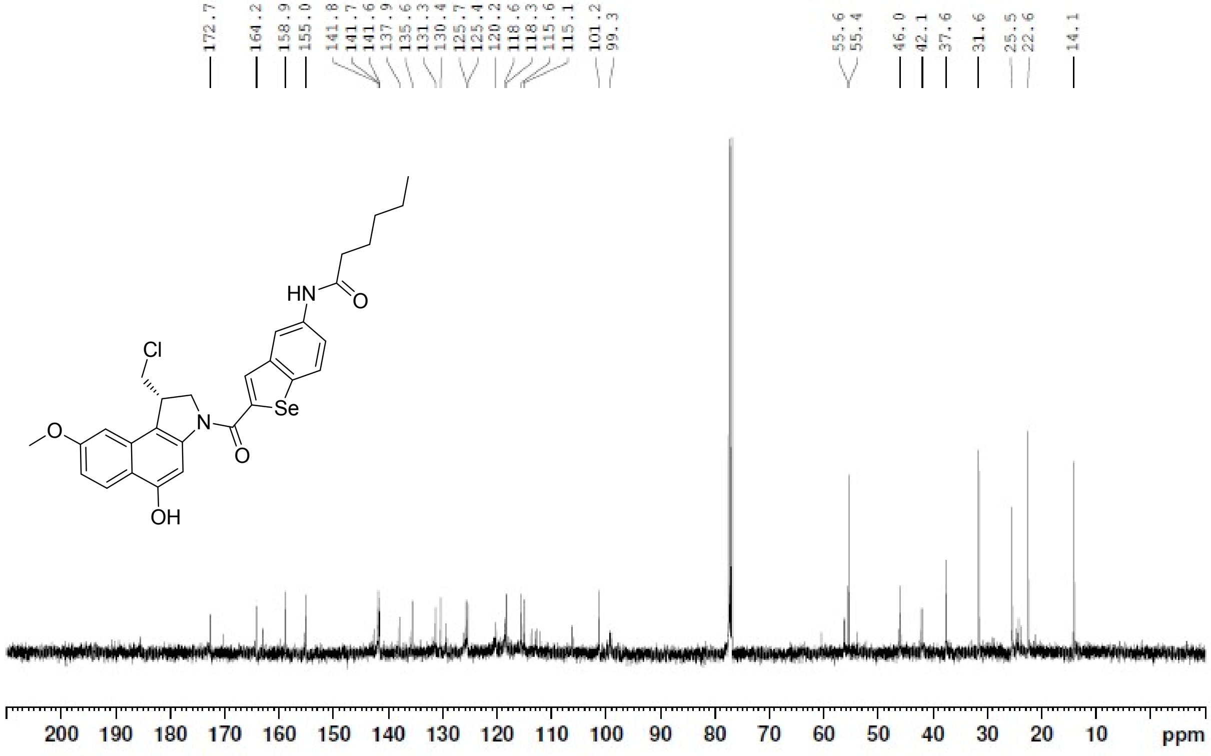

Compound **18t**

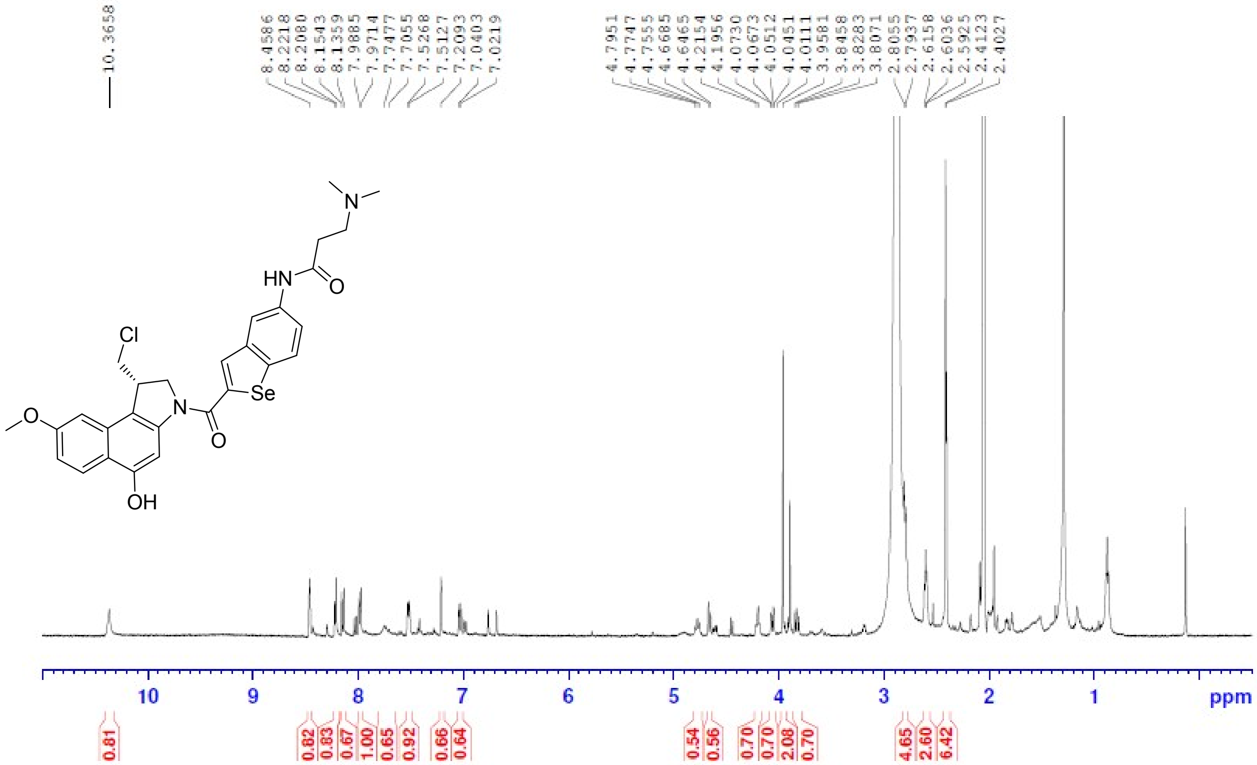

Compound **18u**

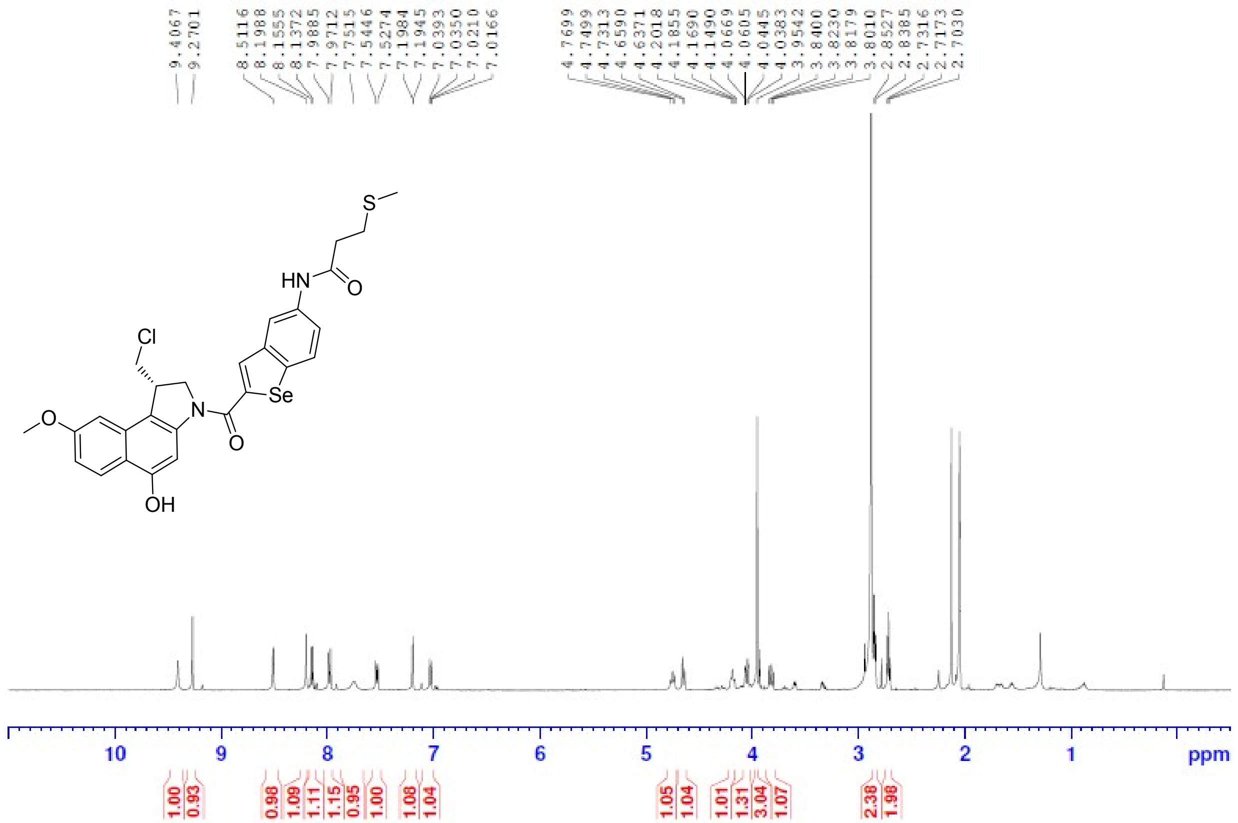

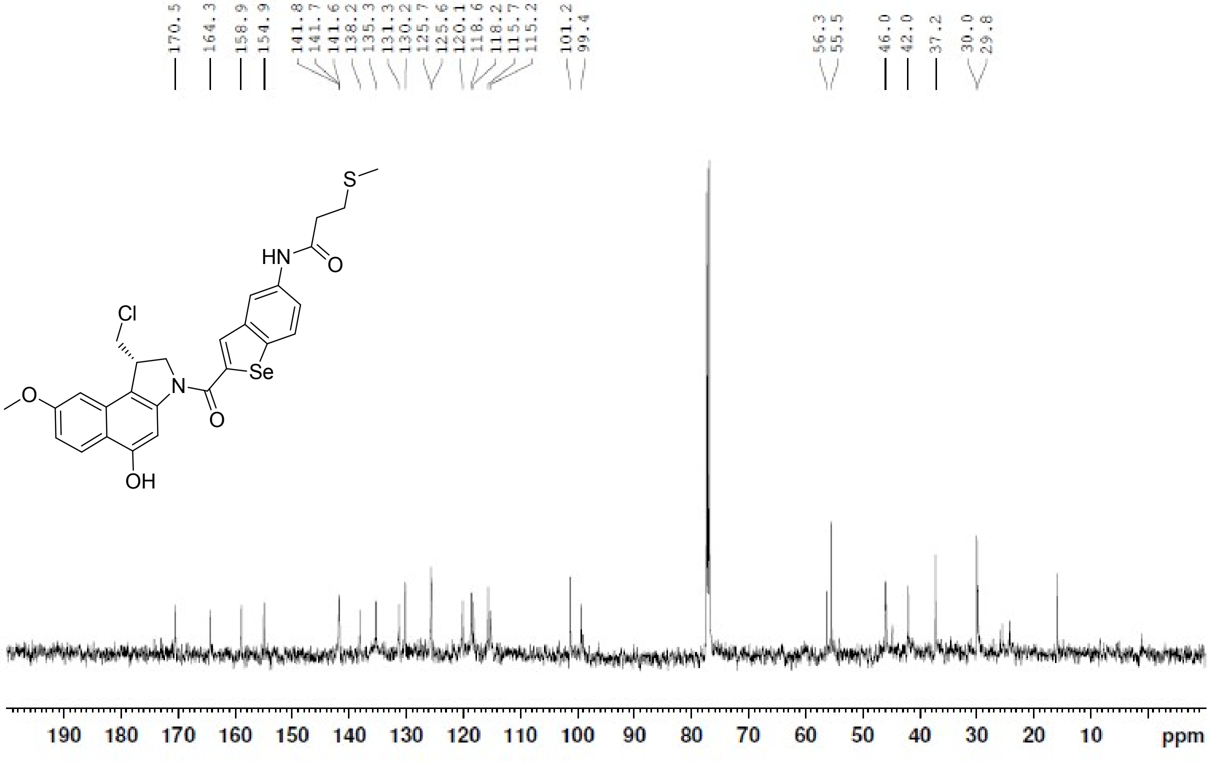

Compound **18v**

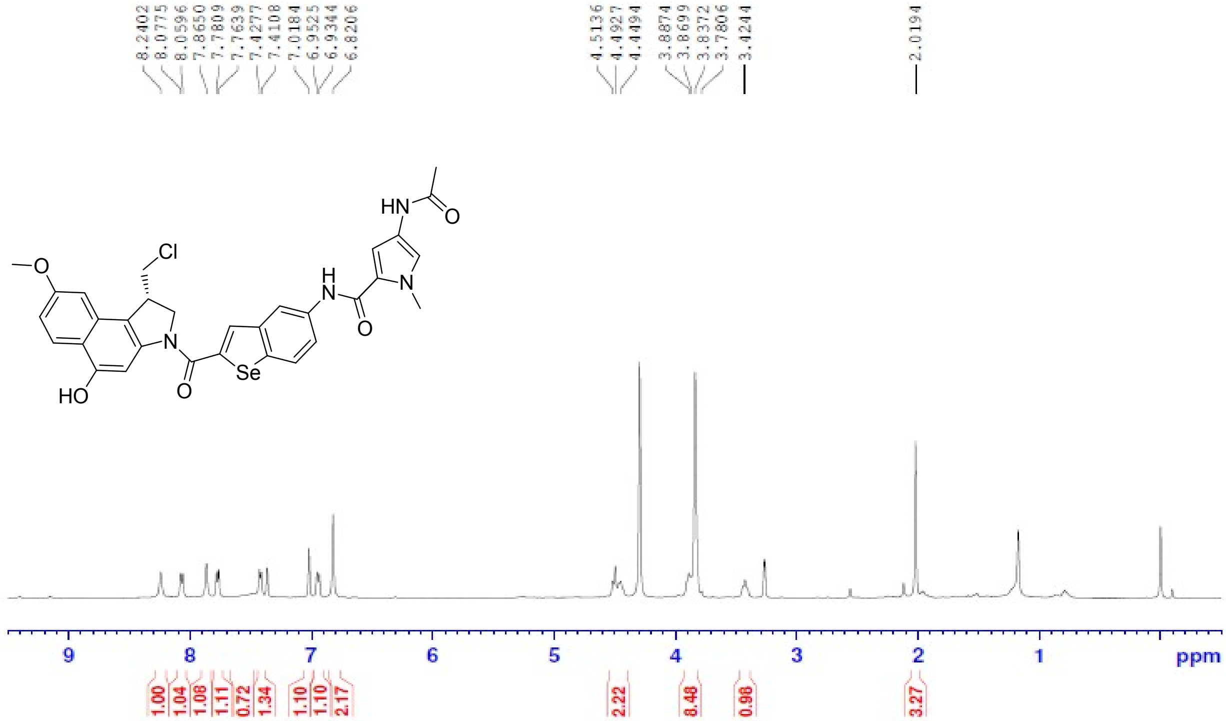

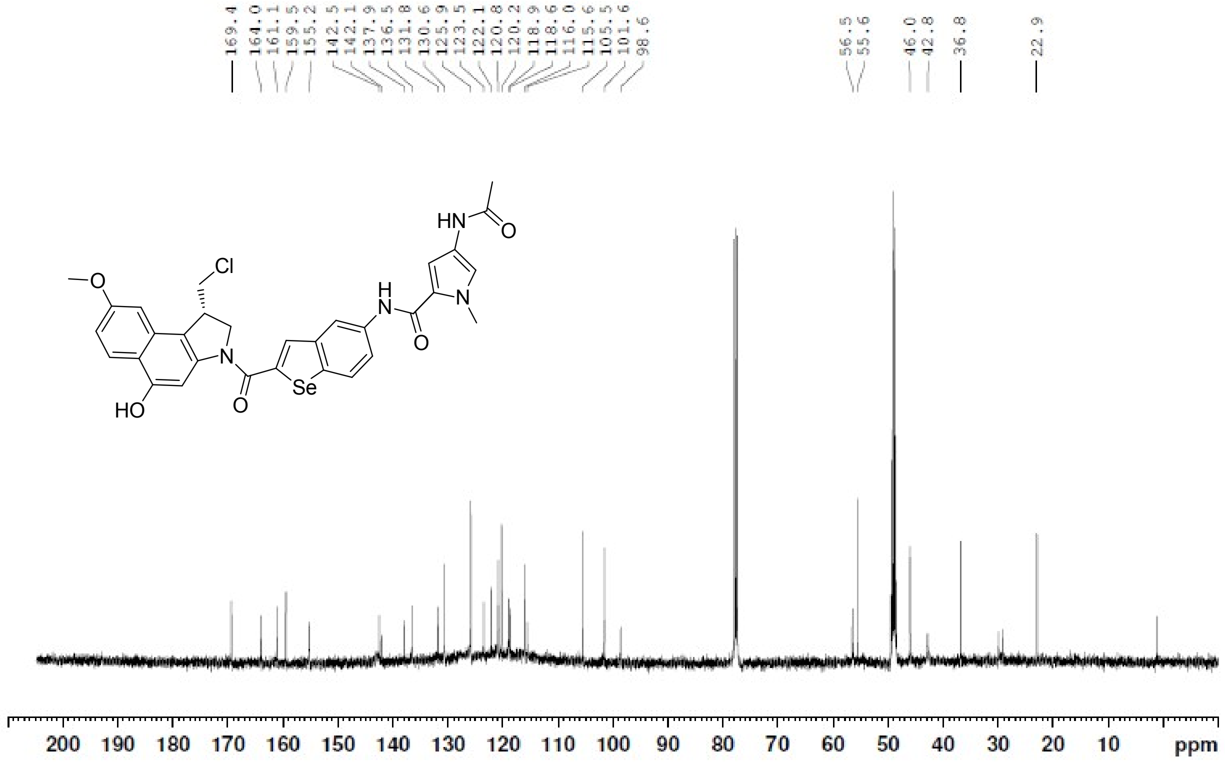

Compound **18w**

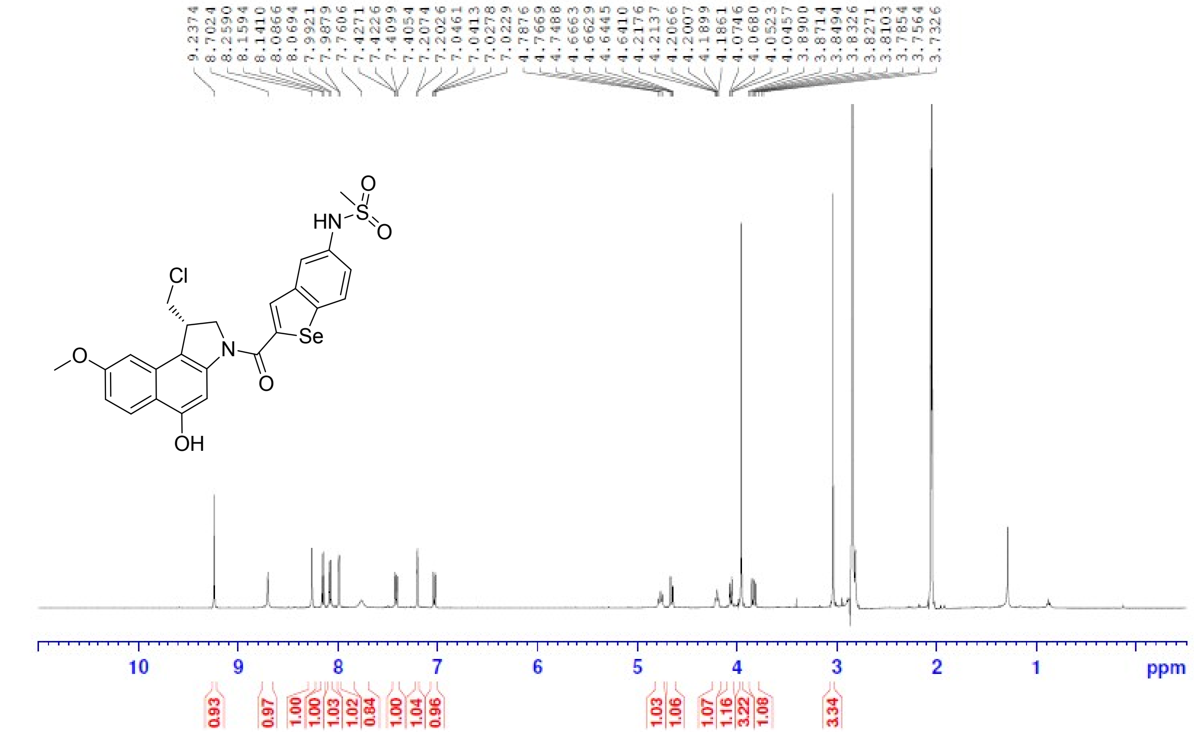

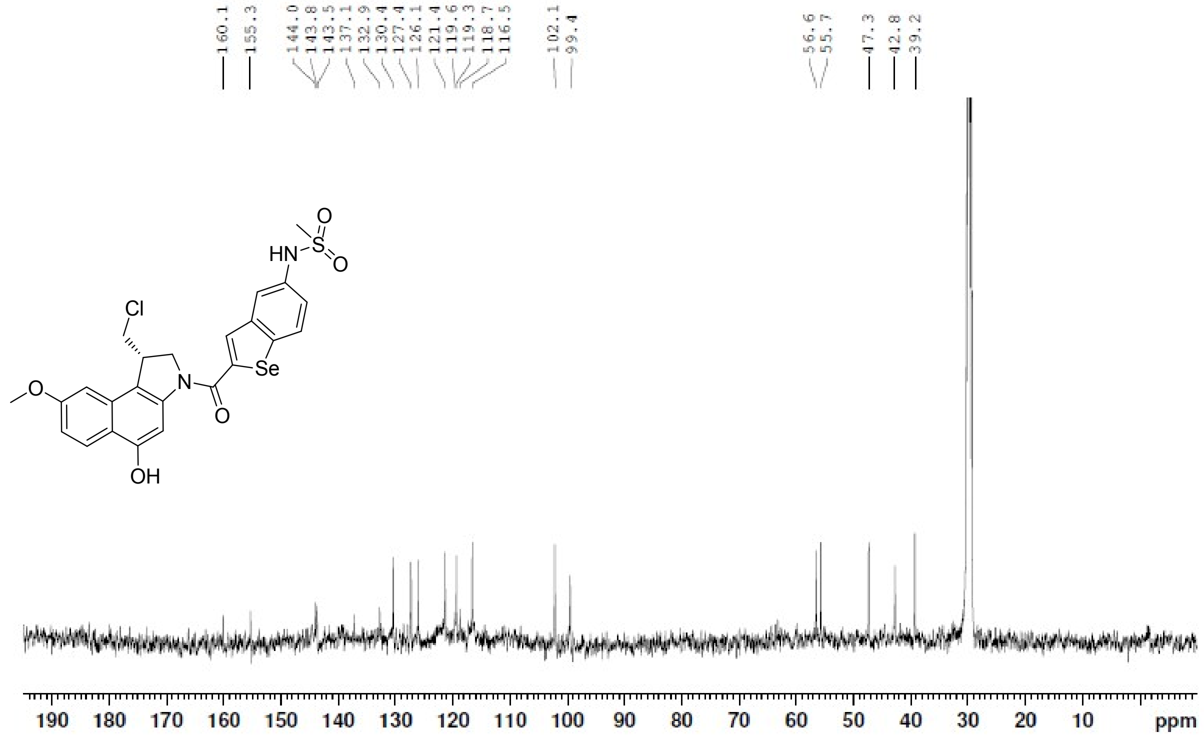

Compound **18x**

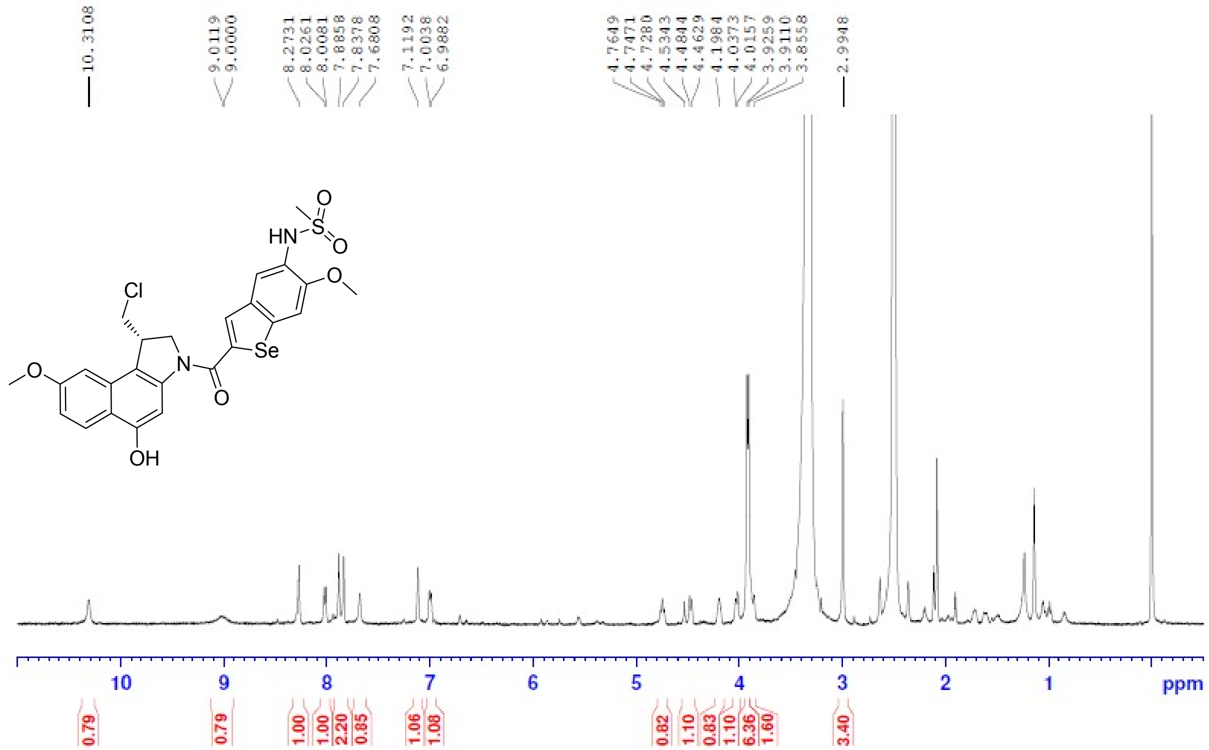

Supplement: RA-009-C9RA04749B-s001 [file RA-009-C9RA04749B-s001.pdf]
